# Synthesis of six-membered spirooxindoles *via* a chiral Brønsted acid-catalyzed asymmetric intramolecular Friedel–Crafts reaction†

**DOI:** 10.1039/c8ra06710d

**Published:** 2018-11-01

**Authors:** Hui-Xuan Chen, Yaqi Zhang, Yuyang Zhang, Xuefeng He, Zhen-Wei Zhang, Hao Liang, Wenhuan He, Xiaoding Jiang, Xiangmeng Chen, Liqin Qiu

**Affiliations:** School of Chemistry, The Key Laboratory of Low-Carbon Chemistry & Energy Conservation of Guangdong Province, South China Sea Bio-Resource Exploitation and Utilization Collaborative Innovation Center, Sun Yat-Sen University No. 135 Xingangxi Road Guangzhou 510275 People's Republic of China qiuliqin@mail.sysu.edu.cn +86-20-8411-0996; College of Pharmacy, Guangxi University of Chinese Medicine Nanning 530200 People's Republic of China

## Abstract

By means of the direct condensation of *N*-aminoethylpyrroles and isatins, followed by a chiral phosphoric acid-catalyzed asymmetric intramolecular Friedel-Crafts reaction, a new class of valuable chiral 3′,4′-dihydro-2′*H*-spiro[indoline-3,1′-pyrrolo[1,2-*a*]pyrazin]-2-ones bearing a quaternary carbon stereocenter were successfully synthesized in good to excellent yields and with moderate to good enantioselectivities under mild reaction conditions.

## Abstract 2

A chiral phosphoric acid-catalyzed asymmetric intramolecular Friedel–Crafts reaction for the synthesis of 3′,4′-dihydro-2′*H*-spiro[indoline-3,1′-pyrrolo[1,2-*a*]pyrazin]-2-ones.

## Graphical abstract

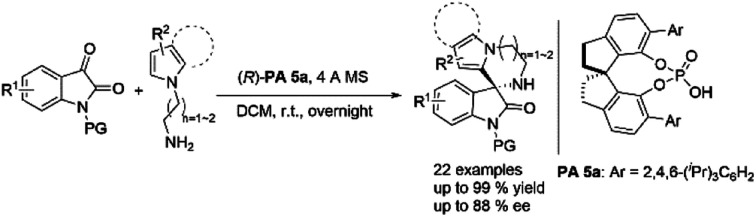

Spirooxindoles, containing a spirocyclic system, are unique structural motifs found in a wide range of natural products and bioactive compounds.^1^ For instance, Spirotryprostatin B (Fig. 1) was isolated from the fermentation broth of *Aspergillus fumigatus* and was shown to completely inhibit the G2/M progression of cell division in mammalian tsFT210 cells.^2^*Gelsemium* alkaloids (*e.g. gelsenicine*, *gelsedine* and *gelsedilam*) were isolated from the ancient medicine Yakatsu stored in the Shosoin Repository and exhibit a wide range of biological activities, including analgesic, anti-inflammatory, and antitumor effects.^3^ Besides, a considerable number of spirooxindoles display anticancer activity. For example, APG-115 and MI77301, which can effectively block the MDM2-p53 protein–protein interaction in cells as MDM2 inhibitors, are under clinical trials as promising anticancer drugs.^4^

**Fig. 1:**
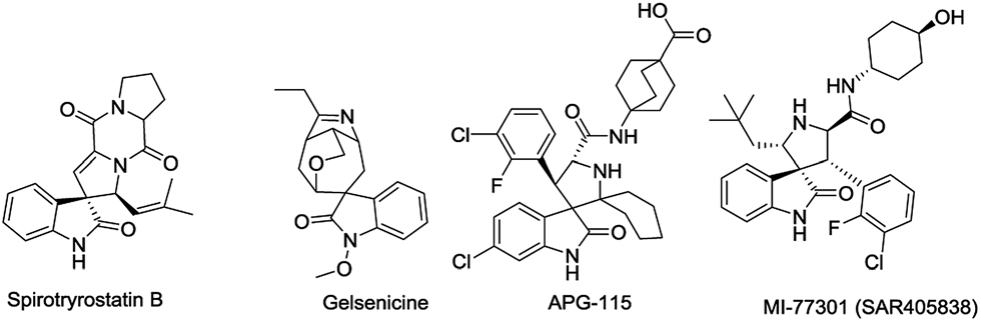
Selected examples of natural and artificial spirooxindoles.

Consequently, the unique structure of those compounds has attracted much attention from synthetic chemists.^5^ Some investigated strategies to access this skeleton include intramolecular reactions of imines *via* Pictet–Spengler reaction synthesized from tryptamines or tryptophans with isatin (Scheme 1a),^6^ oxidative rearrangement of tetrahydro-β-carbolines prepared *via* Pictet–Spengler reaction (Scheme 1b),^7^ metal or small-molecule catalyzed 1,3-dipolar cycloaddition of imino esters with methyleneindolinones (Scheme 1c),^8^ Rh-catalyzed [4 + 1] cycloaddition of azocompound with vinyl isocyanates (Scheme 1d),^9^ chiral iodoarene catalytic oxidative spirocyclization (Scheme 1e),^10^ Pd-catalyzed intramolecular addition and domino spirocyclization,^11^ intramolecular nucleophilic addition^12^ and so on.^13^ Despite of several methods developed for the construction of this skeleton, however, less work finished catalytic asymmetric synthesis and preparation of novel chiral spirooxindoles are warmly anticipated. Herein, we report an efficient protocol *via* an intermolecular condensation/intramolecular Friedel–Crafts reaction to synthesize a new class of 3′,4′-dihydro-2′*H*-spiro[indoline-3,1′-pyrrolo[1,2-*a*]pyrazin]-2-ones in an asymmetric way.

**Scheme 1:**
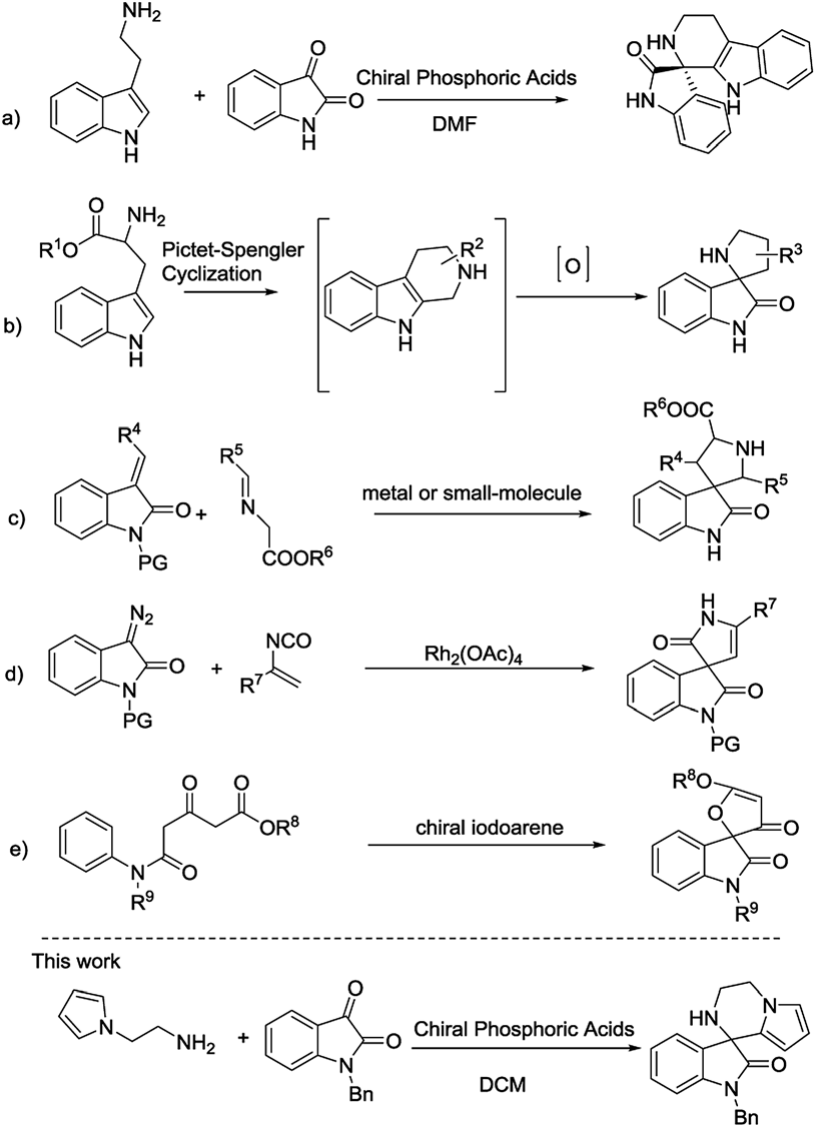
Catalytic synthesis of spirooxindoles.

At the outset of this study, we envisaged that reaction of *N*-aminoethylpyrroles with isatins catalyzed by chiral phosphoric acids might undergo direct condensation, followed by intramolecular Friedel–Crafts reactions under very mild reaction conditions.^14^ We first began our investigation with 1-benzylindole-2,3-dione (1.0 equiv.) and *N*-aminoethylpyrrole (1.2 equiv.) as the substrates, BINOL-derived chiral phosphoric acid 4a as the catalyst, dichloromethane (DCM) as the solvent, 4 Å MS as a desiccant. The reaction was performed at room temperature (20 °C). To our delight, the cascade reaction proceeded smoothly providing 3′,4′-dihydro-2′*H*-spiro[indoline-3,1′-pyrrolo[1,2-*a*]pyrazin]-2-one 3aa in 99% yield but with a poor enantioselectivity (5% ee, Table 1, entry 1). Encouraged by the excellent reactivity, we continued to screen several other chiral phosphoric acid catalysts derived from BINOL under similar reaction conditions (Table 1). When chiral phosphoric acid PA-4c having a substituent 3,5-(CH_3_)_2_C_6_H_3_ at the 3,3′-positions was used, the reaction just only obtained moderate yield and low enantioselectivity (72% yield and 5% ee). Employment of bulkier catalyst (*R*)-PA 4e furnished the product in an excellent yield along with a moderate improvement in the enantioselection (99% yield and 21% ee). Catalysis by 2,4,6-triisopropylphenyl substituted phosphoric acid (*R*)-PA 4i generated good yield and moderate ee (96% yield and 49% ee). Inspired by List's work,^15^ we turned to employ another kind of spiro catalysts^16^ and investigated their function. Delightingly, catalyst (*R*)-PA 5a was found to significantly improve the ee from 49% to 88% and product 3aa was given in 85% yield under mild reaction conditions in DCM. Then we evaluated the feasibility of the reaction in other solvents (Table 1, entries 12–17). It was observed that reaction in tetrahydrofuran had a high reactivity but only a moderate enantioselectivity. The ee values decreased significantly when polar solvents such as acetonitrile and *N*,*N*-dimethylformamide were used (Table 1, entries 13 and 14). Equivalent ee value was achieved using either ether or chloroform (81% and 90% yields; 79% and 78% ee's, respectively; Table 1, entries 15 and 16). Additionally, reaction in 1,4-dioxane acquired good yield and ee (Table 1, entry 17; 83% yield and 83% ee). 1,2-Dichloroethane (DCE) gave similar results as DCM too. Therefore, DCM was selected as the best solvent. Further studies on nitrogen-protecting groups revealed that all reactions proceeded smoothly for different substrates 1 of small or bulky group in this medium. However, negative influences on the enantioselection were showed for the substrates without protecting group or with a small protecting group (39% and 68% ee's, respectively, Table 1, entries 19 and 20). In spite of bulky *N*-protecting group being beneficial to get high yield, the enantioselectivity decreased a little (97%, 84% and 92% yields; 74%, 74% and 69% ee's, respectively; Table 1, entries 21–23). So, benzyl-protected substrate is the best choice for the reaction. Temperature effect was also explored. Conducting the reaction at 0 or 30 °C instead of at room temperature, a noticeable drop in enantioselectivity was observed (79% and 80% ee's respectively, entries 24 and 25). Overall, the model reaction generated the desired product in highest yield in DCM at 20 °C with benzyl-protected 1a as the substrate, PA-5a as the catalyst, 4 Å MS as the desiccant.

**Table 1:** Optimization of the reaction conditions^a^

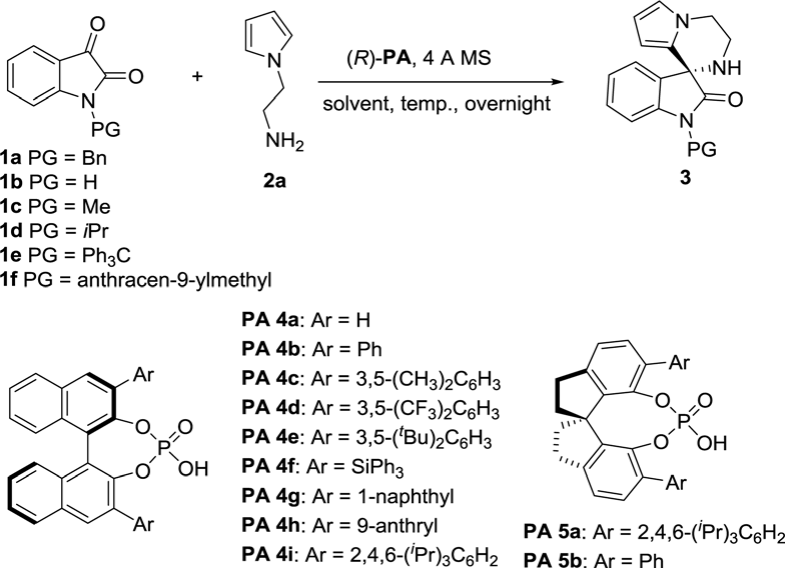						
Entry	Substrate	(*R*)-PA	Solvent	Temp. [°C]	Yield^b^ [%]	ee^c^ [%]
1	1a	4a	DCM	r.t.	99	5
2	1a	4b	DCM	r.t.	99	0
3	1a	4c	DCM	r.t.	72	5
4	1a	4d	DCM	r.t.	99	0
5	1a	4e	DCM	r.t.	99	21
6	1a	4f	DCM	r.t.	99	0
7	1a	4g	DCM	r.t.	99	0
8	1a	4h	DCM	r.t.	97	0
9	1a	4i	DCM	r.t.	96	49
**10**	1a	5a	**DCM**	**r.t.**	**85**	**88**
11	1a	5b	DCM	r.t.	70	19
12	1a	5a	THF	r.t.	99	80
13	1a	5a	MeCN	r.t.	68	62
14	1a	5a	DMF	r.t.	28	44
15	1a	5a	Et_2_O	r.t.	81	79
16	1a	5a	CHCl_3_	r.t.	90	78
17	1a	5a	1,4-Dioxane	r.t.	83	83
18	1a	5a	DCE	r.t.	84	87
19	1b	5a	DCM	r.t.	89	39
20	1c	5a	DCM	r.t.	68	68
21	1d	5a	DCM	r.t.	97	74
22	1e	5a	DCM	r.t.	84	74
23	1f	5a	DCM	r.t.	92	69
24	1a	5a	DCM	0	71	79
25	1a	5a	DCM	30	90	80

a Reaction conditions: 1a (0.2 mmol), 2a (1.2 equiv.), (*R*)-PA (10 mol%), 4 Å MS (50 mg), 2.0 mL of solvent, r.t. = 20 °C, overnight.

b Isolated yield.

c ee was determined by chiral HPLC.

With the set of optimized reaction conditions in hand, we focused our attention on the substrate scope of this catalytic asymmetric intramolecular Friedel–Crafts reaction. At first, substituted isatin derivatives were screened as the substrates in combination with *N*-aminoethyl pyrrole 2a, affording chiral products 3′,4′-dihydro-2′*H*-spiro[indoline-3,1′-pyrrolo[1,2-*a*]pyrazin]-2-ones in moderate to excellent yields (Table 2). Halogen substitutions at C5 positions of the isatins were tolerated but lower ee values were given for 3ga, 3ha and 3ia (79%, 90% and 92% yields; 61%, 37% and 32% ee's respectively). Reaction of isatins bearing an electron-donating group (5-methyl, 3ja and 5-methoxy, 3ka) or a strong electron-withdrawing group (5-nitro, 3la) achieved relatively lower ee values, which was possibly attributed to two reasons: (1) hydrogen-bonding interactions between the isatin substituents and the catalyst; (2) steric hindrance between the isatin substituents and the catalyst. Obviously, strong hydrogen-bonding interaction led to decrease of the ee value for 3ma (15% ee, 4-fluoro). On the contrary, the ee values for 3na and 3oa increased (61% and 61% ee's respectively) when the fluorine group was far from the reactive position. In addition, much bulkier 7-isopropyl substituent proved viable and the corresponding product 3pa was obtained with a good ee value (79% ee).

**Table 2:** Scope of isatin substrates^a^^,^^b^^,^^c^

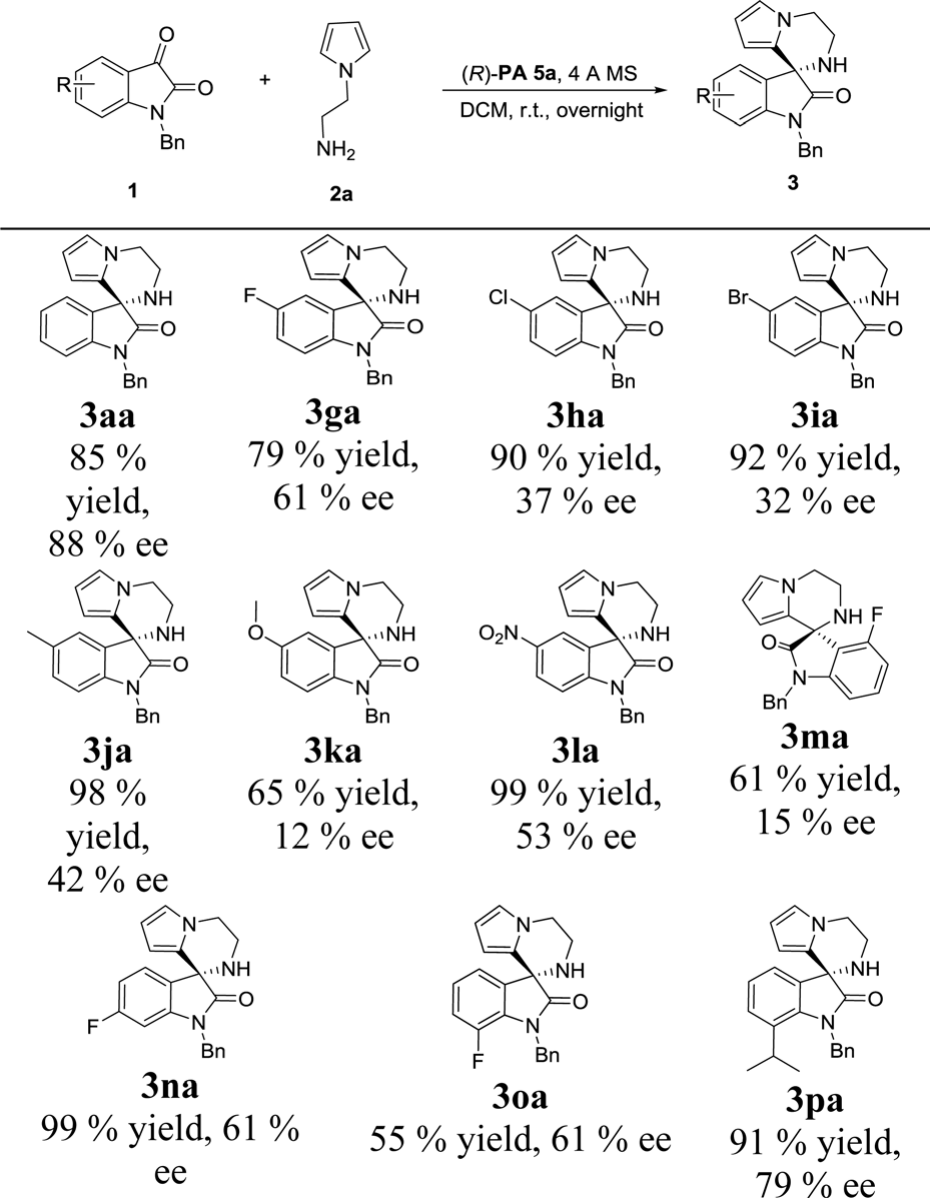

a Reaction conditions: 1a (0.2 mmol), 2a (1.2 equiv.), (*R*)-PA 5a (10 mol%), 4 Å MS (50 mg), 2.0 mL of solvent, overnight.

b Isolated yield.

c ee was determined by chiral HPLC.

Next, the substrate scope of *N*-aminoalkylpyrroles was also investigated. As shown in Table 3, 2-methyl substituted pyrrole maintained good reactivity and provided product 3ab in 79% yield with a lower ee of 49%. Introduction of another methyl group at C3 position of the pyrrole ring resulted in lower reactivity and enantioselectivity in comparison to those of the monosubstituted product 3ab (3ac, Table 3). In the presence of a Brønsted acid (*p*-toluenesulfonic acid), racemic 3ad′ was formed with good regioselectivity (3ad/3ad′ = 5 : 1 determined by ^1^H NMR), while chiral catalyst (*R*)-PA 5a gave nearly equal amounts of 3ad and 3ad′ (3ad/3ad′ = 1 : 1.12). Moderate to good enantioselectivities were obtained (46% and 84% ee's respectively for 3ad and 3ad′, Table 3), albeit with poor regioselectivity. We presumed that the poor regioselectivity might come from the steric hindrance between chiral phosphoric acid PA 5a and the C3-ethyl group of the pyrrole. The cyclization of *N*-aminopropylpyrrole with isatin 1a could also furnish the corresponding product 3ae, but both the reactivity and enantioselectivity were poor (9% yield, 12% ee, Table 3). Employing *N*-aminoethylindole 2f stead of *N*-aminoalkylpyrroles, the obtained result for product 3f was still satisfactory (73% yield and 80% ee). Similar to di-substituted pyrrole, reaction of 3-methylindole also gained a low ee value (3ac and 3ag, Table 3), which could also be attributed to the increased steric hindrance on chiral phosphoric acid PA5a introduced in proximity to the reactive position.

**Table 3:** Scope of pyrrole substrates^a^^,^^b^^,^^c^

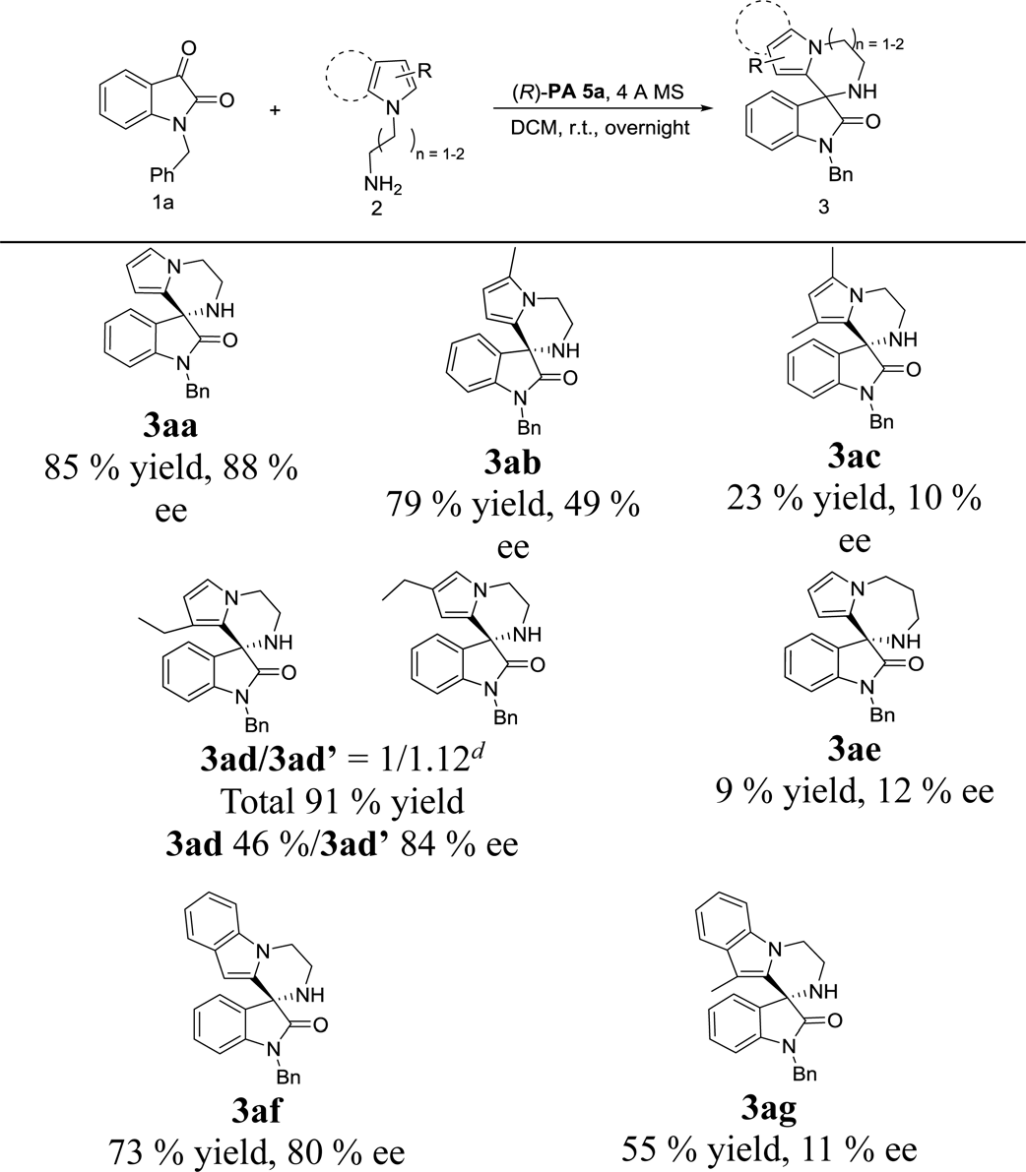

a Reaction conditions: 1a (0.2 mmol), 2a (1.2 equiv.), (*R*)-PA 5a (10 mol%), 4 Å MS (50 mg), 2.0 mL of solvent, overnight.

b Isolated yield.

c ee was determined by chiral HPLC.

Furthermore, a scale up reaction of 1a and 2a was performed, generating product 3aa in 89% yield and 82% ee (Fig. 2).

**Fig. 2:**
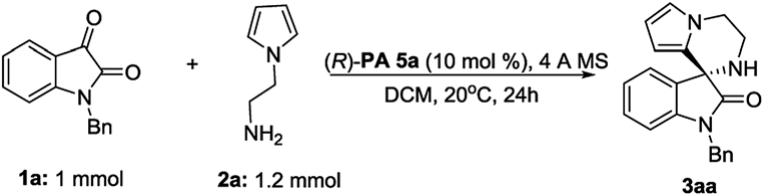
Scale-up reaction.

Finally, based on the experimental results, together with related studies on CPA-catalyzed reactions,^5*f*–5*i*^ we proposed a possible reaction pathway to explain the stereochemistry of the formation of spirooxindoles 3 (Scheme 2). Isatins 1 initially participated in a Mannich reaction with *N-*aminoethylpyrroles 2, affording transient intermediates 6 under the catalysis of Brønsted acid. Through the dual-hydrogen-bond, (*R*)-CPA 5a interacted with intermediates 6 to realize their catalysis and stereocontrol. The enantioenriched spirooxindoles 3 were subsequently yielded *via* the intramolecular Friedel–Crafts reaction of intermediates 6.

**Scheme 2:**
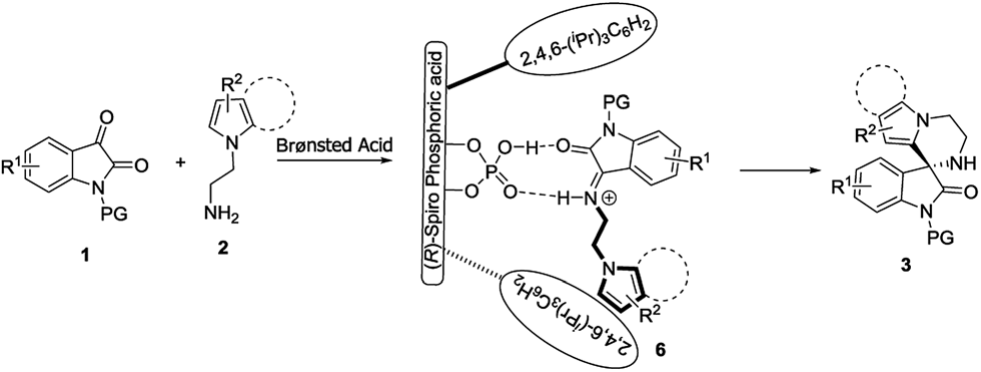
Proposed reaction pathway and activation mode.

## Conclusions

In conclusion, we have developed a direct catalytic asymmetric intramolecular Friedel–Crafts reaction of *N*-aminoethylpyrrole derivatives with isatin derivatives catalyzed by chiral phosphoric acids. This one-pot sequence provides a simple and efficient approach to preparing the new class of 3′,4′-dihydro-2′*H*-spiro[indoline-3,1′-pyrrolo[1,2-*a*]pyrazin]-2-ones in good to excellent yields with moderate to good enantioselectivities under mild reaction conditions. Further work with respect to the extension and applications of this methodology is ongoing in our laboratory.

## Conflicts of interest

There are no conflicts to declare.

## Supplementary Material

RA-008-C8RA06710D-s001
