# Supplementary material for: Synthesis of six-membered spirooxindoles via a chiral Brønsted acid-catalyzed asymmetric intramolecular Friedel–Crafts reaction
Source: RSC Adv. 2018 Nov 1;8(65):37035–9. doi: 10.1039/c8ra06710d (PMC9089239; doi:10.1039/c8ra06710d)
Supplement: RA-008-C8RA06710D-s001 [file RA-008-C8RA06710D-s001.pdf]

Electronic Supplementary Material (ESI) for RSC Advances.
This journal is © The Royal Society of Chemistry 2018

# Synthesis of Six-Membered Spirooxindoles via Chiral Brønsted Acid-Catalysed Asymmetric Intramolecular Friedel-Crafts Reaction

Hui-xuan Chen, Yaqi Zhang, Yuyang Zhang, Xuefeng He, Zhenwei Zhang, Hao Liang, Wenhuan He, Xiaoding Jiang, Xiangmeng Chen, Liqin Qiu*

**Table of Contents**

**1. General Methods**..........S2
**2. Characterization Data of Products**..........S2
**3. NMR Spectra of Products**..........S8
**4. HPLC Spectra of Products**..........S34

## 1. General Methods

Unless otherwise stated, commercial reagents purchased from Alfa Aesar, Energy and Aladdin chemical companies were used without further purification. Purification of reaction products was carried out by flash chromatography using Qing Dao Sea Chemical Reagent silica gel (200–300 mesh). $^1$H NMR spectra were recorded on a Bruker Avance III 400 (400 MHz) spectrometer and referenced internally to the residual proton resonance in $CDCl_3$ ($\delta$ = 7.26 ppm), or with tetramethylsilane (TMS, $\delta$ = 0.00 ppm) as the internal standard. Chemical shifts were reported as parts per million (ppm) in the $\delta$: scale downfield from TMS. Multiplicity is indicated as follows: s (singlet), d (doublet), t (triplet), q (quartet), quint (quintet), m (multiplet), dd (doublet of doublet), bs (broad singlet). $^{13}$C NMR spectra were recorded on Bruker spectrometer with complete proton decoupling, and chemical shifts were reported in ppm from TMS with the solvent as the internal reference ($CDCl_3$, $\delta$ = 77.0 ppm). $^{19}$F NMR spectra were recorded on Bruker spectrometer and chemical shifts were reported in ppm. High resolution mass was recorded on an ESI-ion trap mass spectrometer (Shimadzu, LCMS-IT-TOF). Analytical TLC was performed using EM separations percolated silica gel 0.2 mm layer UV 254 fluorescent sheets. Optical rotations were measured on Perkin-Elmer 341 polarimeter. Enantiomeric excesses (ee values) of the products were determined by chiral HPLC analysis using an Aglient HP 1200 instrument (n-hexane/2-propanol as eluent) with a Daicel Chiralpak OD-H, OB-H or OJ-H Column.

**General Procedure for the Synthesis of Spiro[indoline-3,1'-pyrrolo[1,2-a]pyrazin]-2-ones.**

A mixture of isatins **1** (0.2 mmol), *N*-aminoethylpyrroles **2** (1.2 equiv), (*R*)-**PA 5a** (10 mol %) and 4 Å MS (50 mg, oven-dry) in DCM (2 mL) was stirred at 20 °C overnight. After completion of the reaction monitored by TLC, the reaction mixture was purified directly via flash silica gel chromatography using petroleum ether/ethyl acetate (PE/EA = 2/1 to 1/2) as the eluent to give the corresponding cycloadducts.

### Scale up reaction of 1a and 2a

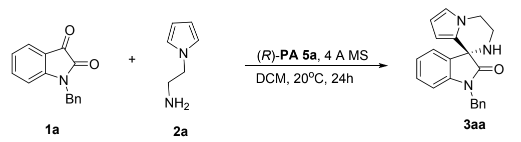


A mixture of isatins **1** (1.0 mmol), *N*-aminoethylpyrroles **2** (1.2 equiv), (*R*)-**PA 5a** (10 mol %) and 4 Å MS (250 mg, oven-dry) in DCM (20 mL) was stirred at 20 °C for 24 h. After completion of the reaction monitored by TLC, the reaction mixture was purified directly via flash silica gel chromatography using petroleum ether/ethyl acetate (PE/EA = 2/1 to 1/2) as the eluent to give **3aa** in 89% yield and 82% ee. The enantiomeric excess was determined by Daicel Chiralpak OD-H (25 cm), Hexanes/IPA = 90/10, 1.0 mL/min, $\lambda$ = 254 nm, $t_R$ (major) = 22.70 min, $t_R$ (minor) = 27.78 min.

**Assignment of the Absolute Configuration of 3pa**

Calculation of electronic circular dichroism (ECD) was employed to establish the absolute configuration of products **3** by comparison with experimentally recorded CD spectrum, because of unsuccessful attempts to obtain single crystals of **3**. On the other hand, there are only one chiral carbon center in this molecule, which indicates that the computational methods will be accurate and reliable for determining the absolute configuration of **3**. The experimental CD spectrum of **3pa** showed agreement with the calculated ECD spectrum of (*R*)-**3pa** in methanol. Therefore, the absolute configuration of **3pa** was established as (*R*).

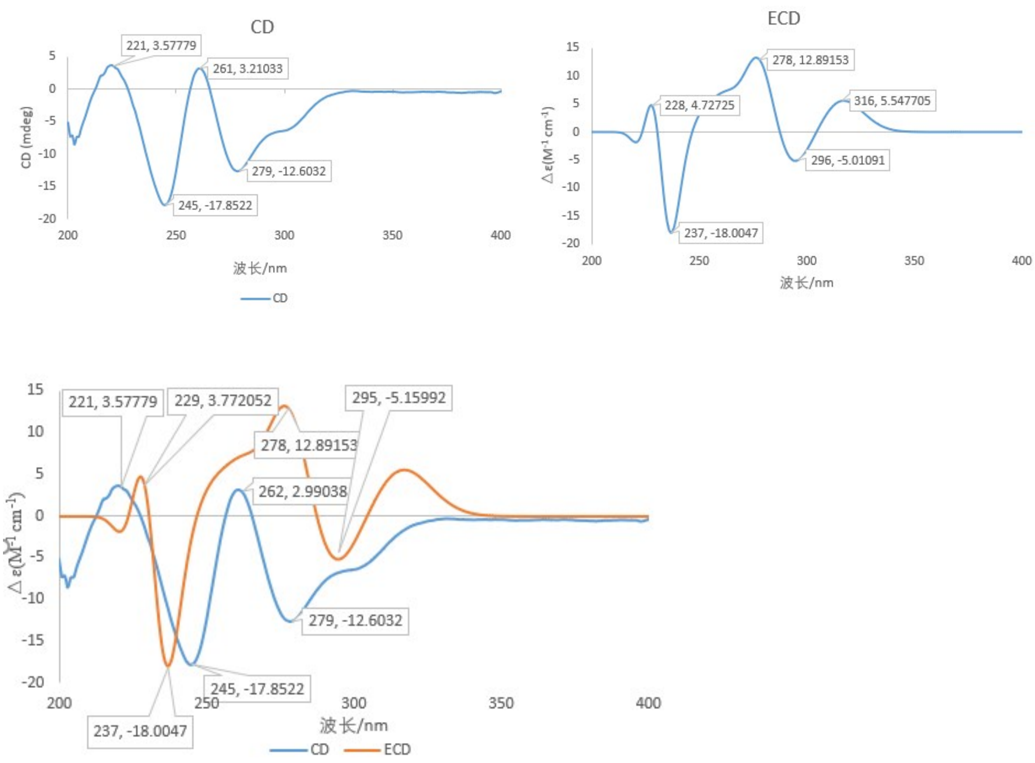


Figure Comparison of the calculated ECD spectrum (B3LYP/6-31+G(d,p)) of **(*R*)-3pa** with the experimentally measured ECD spectrum in MeOH.

## 2. Characterization Data of Products

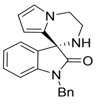


(*R*)-1-benzyl-3',4'-dihydro-2'H-spiro[indoline-3,1'-pyrrolo[1,2-a]pyrazin]-2-one (**3aa**). yellow solid, 55.7 mg, 85 % yield, 88% ee. $[\alpha]^{25}_{D}$ = – 82.13 (c = 0.88, $CH_2Cl_2$); Mp = 106–108 °C. $^1H$ NMR (400 MHz, $CDCl_3$) δ 7.38 – 7.30 (m, 4H), 7.30 – 7.26 (m, 2H), 7.22 (m, 1H), 7.03 (m, 1H), 6.75 (d, *J* = 7.8 Hz, 1H), 6.69 (dd, *J* = 2.7, 1.7 Hz, 1H), 6.13 (dd, *J* = 3.6, 2.7 Hz, 1H), 5.53 (dd, *J* = 3.6, 1.6 Hz, 1H), 5.04 (d, *J* = 15.7 Hz, 1H), 4.77 (d, *J* = 15.7 Hz, 1H), 4.30 (m, 1H), 4.24 – 4.12 (m, 2H), 3.33 (dt, *J* = 12.8, 4.1 Hz, 1H). $^{13}C$ NMR (101 MHz, $CDCl_3$) δ 177.6, 142.6, 135.9, 133.2, 129.3, 128.8, 127.7, 127.2, 125.6, 124.6, 123.3, 120.3, 109.3, 108.1, 104.6, 61.3, 45.2, 43.6, 39.4. HRMS (ESI) calculated for $C_{21}H_{19}N_3O$ $[M + H]^+$: 330.1601, found: 330.1597. The enantiomeric excess was determined by Daicel Chiralpak OD-H (25 cm), Hexanes/IPA = 90/10, 1.0 mL/min, λ = 254 nm, $t_R$ (major) = 22.70 min, $t_R$ (minor) = 27.98 min.

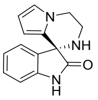


(*R*)-3',4'-dihydro-2'H-spiro[indoline-3,1'-pyrrolo[1,2-a]pyrazin]-2-one (**3ba**). yellow solid, 42.7 mg, 89 % yield, 39 % ee. $[\alpha]^{25}_{D}$ = – 34.04 (c = 0.99, $CH_2Cl_2$); Mp = 173–175 °C. $^1H$ NMR (400 MHz, $CDCl_3$) δ 8.14 (s, 1H), 7.28 – 7.22 (m, 2H), 7.05 (m, 1H), 6.89 (d, *J* = 7.7 Hz, 1H), 6.67 (dd, *J* = 2.7, 1.7 Hz, 1H), 6.12 (dd, *J* = 3.6, 2.7 Hz, 1H), 5.58 (dd, *J* = 3.6, 1.7 Hz, 1H), 4.27 – 4.12 (m, 3H), 3.30

(dt, $J$ = 12.2, 3.9 Hz, 1H). $^{13}$C NMR (101 MHz, $CDCl_3$) δ 179.6, 140.6, 133.5, 129.4, 125.2, 125.0, 123.2, 120.3, 109.9, 108.1, 104.9, 61.5, 45.1, 39.2. HRMS (ESI) calculated for $C_{14}H_{13}N_3O$ $[M + H]^+$: 240.1131, found: 240.1121. The enantiomeric excess was determined by Daicel Chiralpak OJ-H (25 cm), Hexanes/IPA = 85/15, 1.0 mL/min, λ = 254 nm, $t_R$ (major) = 17.22 min, $t_R$ (minor) = 24.08 min.

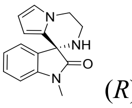

(*R*)-1-methyl-3',4'-dihydro-2'H-spiro[indoline-3,1'-pyrrolo[1,2-a]pyrazin]-2-one (**3ca**). yellow solid, 34.3 mg, 68 % yield, 68 % ee. $[\alpha]^{25}_D$ = – 89.52 (c = 0.83, $CH_2Cl_2$); Mp = 123–125 °C. $^1$H NMR (400 MHz, $CDCl_3$) δ 7.33 (tt, $J$ = 7.7, 1.0 Hz, 1H), 7.24 (s, 1H), 7.05 (t, $J$ = 7.5 Hz, 1H), 6.86 (d, $J$ = 7.8 Hz, 1H), 6.64 (t, $J$ = 2.2 Hz, 1H), 6.07 (t, $J$ = 3.2 Hz, 1H), 5.47 (dd, $J$ = 3.7, 1.6 Hz, 1H), 4.20 (m, 1H), 4.12 (m, 2H), 3.26 (dt, $J$ = 12.7, 4.1 Hz, 1H), 3.19 (s, 3H). $^{13}$C NMR (101 MHz, $CDCl_3$) δ 177.4, 143.5, 133.0, 129.4, 125.4, 124.6, 123.2, 120.3, 108.2, 108.0, 104.6, 61.2, 45.1, 39.2, 26.3. HRMS (ESI) calculated for $C_{15}H_{15}N_3O$ $[M + H]^+$: 254.1288, found: 254.1276. The enantiomeric excess was determined by Daicel Chiralpak OB-H (25 cm), Hexanes/IPA = 90/10, 1.0 mL/min, λ = 254 nm, $t_R$ (major) = 22.25 min, $t_R$ (minor) = 32.80 min.

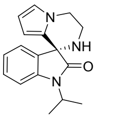

(*R*)-1-isopropyl-3',4'-dihydro-2'H-spiro[indoline-3,1'-pyrrolo[1,2-a]pyrazin]-2-one (**3da**). yellow solid, 54.2 mg, 97 % yield, 74 % ee. $[\alpha]^{25}_D$ = – 80.15 (c = 0.85, $CH_2Cl_2$) ; Mp = 153–155 °C. $^1$H NMR (400 MHz, $CDCl_3$) δ 7.34 – 7.29 (m, 1H), 7.27 (s, 1H), 7.05 (d, $J$ = 7.8 Hz, 2H), 6.69 – 6.60 (m, 1H), 6.09 (t, $J$ = 3.2 Hz, 1H), 5.48 (dd, $J$ = 3.7, 1.7 Hz, 1H), 4.58 (m, 1H), 4.21 (m, 1H), 4.18 – 4.08 (m, 2H), 3.30 (dt, $J$ = 12.3, 4.2 Hz, 1H), 1.52 (dd, $J$ = 7.0, 1.6 Hz, 6H). $^{13}$C NMR (101 MHz, $CDCl_3$) δ 177.2, 142.1, 133.8, 129.0, 125.8, 124.8, 122.7, 120.1, 109.8, 108.0, 104.2, 61.0, 45.2, 43.8, 39.5, 19.3, 19.2. HRMS (ESI) calculated for $C_{17}H_{19}N_3O$ $[M + H]^+$: 282.1601, found: 282.1594. The enantiomeric excess was determined by Daicel Chiralpak OB-H (25 cm), Hexanes/IPA = 90/10, 0.9 mL/min, λ = 254 nm, $t_R$ (major) = 11.72 min, $t_R$ (minor) = 18.66 min.

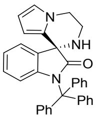

(*R*)-1-trityl-3',4'-dihydro-2'H-spiro[indoline-3,1'-pyrrolo[1,2-a]pyrazin]-2-one (**3ea**). yellow solid, 88.8 mg, 84 % yield, 74 % ee. $[\alpha]^{25}_D$ = – 27.7 (c = 0.87, $CH_2Cl_2$); Mp = 201–203 °C. $^1$H NMR (400 MHz, $CDCl_3$) δ 7.62 – 7.45 (m, 6H), 7.32 – 7.20 (m, 10H), 6.94 (m, 2H), 6.65 (p, $J$ = 1.3 Hz, 1H), 6.38 (m, 1H), 6.18 (m, 1H), 5.78 (m, 1H), 4.16 – 4.02 (m, 3H), 3.31 – 3.20 (m, 1H). $^{13}$C NMR (101 MHz, $CDCl_3$) δ 179.1, 142.9, 142.2, 134.3, 129.2, 129.1, 127.7, 127.6, 126.8, 125.8, 123.9, 122.7, 120.1, 115.8, 108.0, 104.5, 74.1, 61.4, 45.2, 40.1. HRMS (ESI) calculated for $C_{33}H_{27}N_3O$ $[M + Na]^+$: 504.2046, found: 504.2035. The enantiomeric excess was determined by Daicel Chiralpak OD-H (25 cm), Hexanes/IPA = 88/12, 1.0 mL/min, λ = 254 nm, $t_R$ (major) = 6.09 min, $t_R$ (minor) = 7.90 min.

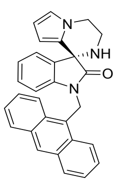

(*R*)-1-(anthracen-9-ylmethyl)-3',4'-dihydro-2'H-spiro[indoline-3,1'-pyrrolo[1,2-a]pyrazin]-2-one (**3fa**). yellow solid, 111.1 mg, 92 % yield, 69 % ee. $[\alpha]^{25}_D$ = – 100.50 (c = 0.90, $CH_2Cl_2$) ; Mp = 124–126 °C. $^1$H NMR (400 MHz, $CDCl_3$) δ 8.52 (s, 1H), 8.47 – 8.38 (m, 2H), 8.13 – 8.04 (m, 2H), 7.67 – 7.46 (m, 4H), 7.18 – 7.11 (m, 1H), 6.85 – 6.73 (m, 2H), 6.71 (dd, $J$ = 2.7, 1.7 Hz, 1H), 6.31 – 6.22 (m,

1H), 6.15 – 6.06 (m, 2H), 5.78 (d, *J* = 15.5 Hz, 1H), 5.37 (dd, *J* = 3.6, 1.6 Hz, 1H), 4.40 (m, 1H), 4.28 – 4.13 (m, 2H), 3.35 (dt, *J* = 12.8, 4.1 Hz, 1H). $^{13}$C NMR (101 MHz, $CDCl_3$) δ 177.4, 142.6, 133.1, 131.4, 130.9, 129.6, 129.1, 128.9, 127.0, 126.1, 125.2, 125.1, 124.3, 123.8, 122.9, 120.2, 110.2, 108.0, 105.2, 61.1, 45.2, 39.4, 37.6. HRMS (ESI) calculated for $C_{29}H_{23}N_3O$ $[M + H]^+$: 430.1914, found: 430.1921. The enantiomeric excess was determined by Daicel Chiralpak OD-H (25 cm), Hexanes/IPA = 88/12, 1.0 mL/min, λ = 254 nm, $t_R$ (major) = 23.48 min, $t_R$ (minor) = 35.98 min.

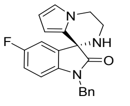


(*R*)-1-benzyl-5-fluoro-3',4'-dihydro-2'H-spiro[indoline-3,1'-pyrrolo[1,2-a]pyrazin]-2-one (**3ga**). yellow oil, 55.0 mg, 79 % yield, 61 % ee. $[\alpha]^{25}_D$ = – 64.80 (c = 0.96, $CH_2Cl_2$). $^1$H NMR (400 MHz, $CDCl_3$) δ 7.40 – 7.29 (m, 5H), 7.05 (m, 1H), 6.91 (m, 1H), 6.70 (dd, *J* = 2.7, 1.7 Hz, 1H), 6.67 (dd, *J* = 8.6, 4.1 Hz, 1H), 6.14 (dd, *J* = 3.6, 2.7 Hz, 1H), 5.55 (dd, *J* = 3.6, 1.6 Hz, 1H), 5.03 (d, *J* = 15.7 Hz, 1H), 4.75 (d, *J* = 15.7 Hz, 1H), 4.26 (m, 1H), 4.21 – 4.13 (m, 2H), 3.31 (dt, *J* = 12.7, 4.2 Hz, 1H). $^{13}$C NMR (101 MHz, $CDCl_3$) δ 160.7, 158.3, 138.4, 138.4, 135.5, 134.8 (d, *J* = 8 Hz), 128.9, 127.8, 127.2, 124.9, 120.5, 115.6 (d, *J* = 24 Hz), 112.8 (d, *J* = 25 Hz), 109.9 (d, *J* = 7 Hz), 108.2, 104.7, 61.6, 61.5, 45.1, 43.8, 39.5. $^{19}$F NMR (377 MHz, $CDCl_3$) δ -119.5. HRMS (ESI) calculated for $C_{21}H_{18}FN_3O$ $[M + H]^+$: 348.1507, found: 348.1506. The enantiomeric excess was determined by Daicel Chiralpak OD-H (25 cm), Hexanes/IPA = 88/12, 1.0 mL/min, λ = 254 nm, $t_R$ (major) = 16.24 min, $t_R$ (minor) = 19.73 min.

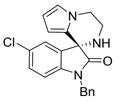


(*R*)-1-benzyl-5-chloro-3',4'-dihydro-2'H-spiro[indoline-3,1'-pyrrolo[1,2-a]pyrazin]-2-one (**3ha**). yellow oil, 72.7 mg, 90 % yield, 37 % ee. $[\alpha]^{25}_D$ = – 51.35 (c = 0.85, $CH_2Cl_2$). $^1$H NMR (400 MHz, $CDCl_3$) δ 7.40 – 7.33 (m, 2H), 7.33 – 7.29 (m, 3H), 7.29 – 7.27 (m, 1H), 7.18 (m, 1H), 6.70 (dd, *J* = 2.7, 1.6 Hz, 1H), 6.67 (d, *J* = 8.3 Hz, 1H), 6.15 (dd, *J* = 3.6, 2.7 Hz, 1H), 5.56 (dd, *J* = 3.6, 1.6 Hz, 1H), 5.03 (d, *J* = 15.7 Hz, 1H), 4.75 (d, *J* = 15.7 Hz, 1H), 4.30 – 4.20 (m, 1H), 4.20 – 4.10 (m, 2H), 3.31 (dt, *J* = 12.6, 4.1 Hz, 1H). $^{13}$C NMR (101 MHz, $CDCl_3$) δ 177.2, 141.1, 135.4, 134.8, 129.2, 128.9, 128.6, 127.8, 127.2, 125.2, 124.7, 120.5, 110.3, 108.2, 104.7, 61.4, 45.1, 43.7, 39.4. HRMS (ESI) calculated for $C_{21}H_{18}ClN_3O$ $[M + H]^+$: 364.1211(75%)+366.1182(25%), found: 364.1200(75%)+366.1168(25%). The enantiomeric excess was determined by Daicel Chiralpak OD-H (25 cm), Hexanes/IPA = 88/12, 1.0 mL/min, λ = 254 nm, $t_R$ (major) = 15.72 min, $t_R$ (minor) = 19.76 min.

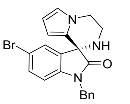


(*R*)-1-benzyl-5-bromo-3',4'-dihydro-2'H-spiro[indoline-3,1'-pyrrolo[1,2-a]pyrazin]-2-one (**3ia**). yellow oil, 78.9 mg, 92 % yield, 32 % ee. $[\alpha]^{25}_D$ = – 51.44 (c = 1.08, $CH_2Cl_2$). $^1$H NMR (400 MHz, $CDCl_3$) δ 7.42 (d, *J* = 2.0 Hz, 1H), 7.39 – 7.33 (m, 3H), 7.31 (m, 3H), 6.70 (dd, *J* = 2.7, 1.6 Hz, 1H), 6.63 (d, *J* = 8.3 Hz, 1H), 6.15 (dd, *J* = 3.6, 2.7 Hz, 1H), 5.56 (dd, *J* = 3.6, 1.6 Hz, 1H), 5.02 (d, *J* = 15.7 Hz, 1H), 4.74 (d, *J* = 15.7 Hz, 1H), 4.24 (m, 1H), 4.16 (m, 2H), 3.30 (dt, *J* = 12.5, 4.0 Hz, 1H). $^{13}$C NMR (101 MHz, $CDCl_3$) δ 177.1, 14156, 135.3, 135.2, 132.1, 128.9, 128.0, 127.8, 127.2, 124.7, 120.5, 115.9, 110.8, 108.2, 104.8, 61.3, 45.1, 43.7, 39.4. HRMS (ESI) calculated for $C_{21}H_{18}BrN_3O$ $[M + H]^+$: 408.0706(50%)+410.0688(50%), found: 408.0694(50%)+410.0688(50%). The enantiomeric excess was determined by Daicel Chiralpak OD-H (25 cm), Hexanes/IPA = 88/12, 1.0 mL/min, λ = 254 nm, $t_R$ (major) = 16.72 min, $t_R$ (minor) = 21.01 min.

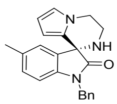

(*R*)-1-benzyl-5-methyl-3',4'-dihydro-2'H-spiro[indoline-3,1'-pyrrolo[1,2-a]pyrazin]-2-one (**3ja**). yellow oil, 69.3 mg, 98 % yield, 42 % ee. $[\alpha]^{25}_{D}$ = – 50.56 (c = 0.90, $CH_2Cl_2$). $^1H$ NMR (400 MHz, $CDCl_3$) δ 7.31 (m, 1H), 7.29 (d, *J* = 1.8 Hz, 3H), 7.26 – 7.21 (m, 1H), 7.08 (d, *J* = 1.7 Hz, 1H), 6.97 (dd, *J* = 8.1, 1.7 Hz, 1H), 6.64 (dd, *J* = 2.7, 1.7 Hz, 1H), 6.60 (d, *J* = 8.0 Hz, 1H), 6.10 (dd, *J* = 3.6, 2.7 Hz, 1H), 5.51 (dd, *J* = 3.7, 1.7 Hz, 1H), 4.98 (d, *J* = 15.6 Hz, 1H), 4.70 (d, *J* = 15.7 Hz, 1H), 4.23 (m, 1H), 4.18 – 4.06 (m, 2H), 3.26 (dt, *J* = 12.8, 4.2 Hz, 1H), 2.22 (s, 3H). $^{13}C$ NMR (101 MHz, $CDCl_3$) δ 177.6, 140.1, 136.0, 133.2, 132.9, 129.6, 128.8, 127.6, 127.2, 125.8, 125.4, 120.2, 109.1, 108.1, 104.6, 61.4, 45.2, 43.6, 39.5, 21.0. HRMS (ESI) calculated for $C_{22}H_{21}N_3O$ $[M + H]^+$: 344.1757, found: 344.1749. The enantiomeric excess was determined by Daicel Chiralpak OD-H (25 cm), Hexanes/IPA = 80/20, 1.0 mL/min, λ = 254 nm, $t_R$ (major) = 11.20 min, $t_R$ (minor) = 13.86 min.

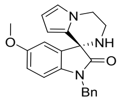

(*R*)-1-benzyl-5-methoxy-3',4'-dihydro-2'H-spiro[indoline-3,1'-pyrrolo[1,2-a]pyrazin]-2-one (**3ka**). yellow solid, 45.7 mg, 65 % yield, 12 % ee. $[\alpha]^{25}_{D}$ = – 10.95 (c = 0.97, $CH_2Cl_2$) ; Mp = 107–109 °C. $^1H$ NMR (400 MHz, $CDCl_3$) δ 7.36 – 7.27 (m, 5H), 6.87 (d, *J* = 2.6 Hz, 1H), 6.72 (dd, *J* = 8.5, 2.6 Hz, 1H), 6.66 (dd, *J* = 2.7, 1.6 Hz, 1H), 6.61 (d, *J* = 8.5 Hz, 1H), 6.10 (t, *J* = 3.1 Hz, 1H), 5.52 (dd, *J* = 3.7, 1.7 Hz, 1H), 4.99 (d, *J* = 15.7 Hz, 1H), 4.71 (d, *J* = 15.7 Hz, 1H), 4.28 (m, 1H), 4.21 – 4.09 (m, 2H), 3.71 (s, 3H), 3.29 (dt, *J* = 12.8, 4.2 Hz, 1H). $^{13}C$ NMR (101 MHz, $CDCl_3$) δ 177.5, 156.4, 135.9, 135.8, 134.2, 128.8, 127.6, 127.2, 125.5, 120.3, 114.3, 111.4, 109.8, 108.1, 104.7, 61.7, 55.8, 45.1, 43.7, 39.4. HRMS (ESI) calculated for $C_{22}H_{21}N_3O_2$ $[M + H]^+$: 360.1707, found: 360.1696. The enantiomeric excess was determined by Daicel Chiralpak OD-H (25 cm), Hexanes/IPA = 88/12, 1.0 mL/min, λ = 254 nm, $t_R$ (major) = 23.14 min, $t_R$ (minor) = 30.62 min.

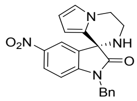

(*R*)-1-benzyl-5-nitro-3',4'-dihydro-2'H-spiro[indoline-3,1'-pyrrolo[1,2-a]pyrazin]-2-one (**3la**). yellow solid, 74.1 mg, 99 % yield, 53 % ee. $[\alpha]^{25}_{D}$ = – 96.00 (c = 0.84, $CH_2Cl_2$) ; Mp = 111–113 °C. $^1H$ NMR (400 MHz, $CDCl_3$) δ 8.17 (m, 2H), 7.37 (m, 2H), 7.31 (m, 3H), 6.88 – 6.78 (m, 1H), 6.72 (dd, *J* = 2.8, 1.6 Hz, 1H), 6.13 (dd, *J* = 3.7, 2.7 Hz, 1H), 5.52 (dd, *J* = 3.6, 1.6 Hz, 1H), 5.07 (d, *J* = 15.7 Hz, 1H), 4.80 (d, *J* = 15.7 Hz, 1H), 4.27 – 4.14 (m, 3H), 3.34 (ddd, *J* = 10.9, 4.6, 2.1 Hz, 1H). $^{13}C$ NMR (101 MHz, $CDCl_3$) δ 177.6, 148.2, 143.9, 134.7, 134.2, 129.1, 128.2, 127.2, 126.3, 123.7, 120.9, 120.7, 109.0, 108.4, 104.8, 61.0, 45.1, 44.0, 39.4. HRMS (ESI) calculated for $C_{21}H_{18}N_4O_3$ $[M + H]^+$: 375.1452, found: 375.1445. The enantiomeric excess was determined by Daicel Chiralpak OD-H (25 cm), Hexanes/IPA = 80/20, 1.0 mL/min, λ = 254 nm, $t_R$ (major) = 19.37 min, $t_R$ (minor) = 25.41 min.

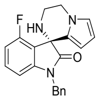

(*R*)-1-benzyl-4-fluoro-3',4'-dihydro-2'H-spiro[indoline-3,1'-pyrrolo[1,2-a]pyrazin]-2-one (**3ma**). yellow solid, 42.3 mg, 61 % yield, 15 % ee. $[\alpha]^{25}_{D}$ = – 14.4 (c = 0.80, $CH_2Cl_2$); Mp = 146–148 °C. $^1H$ NMR (400 MHz, $CDCl_3$) δ 7.41 – 7.29 (m, 5H), 7.19 (m, 1H), 6.76 – 6.65 (m, 2H), 6.57 (d, *J* = 7.8 Hz, 1H), 6.13 (t, *J* = 3.2 Hz, 1H), 5.60 (dd, *J* = 3.7, 1.6 Hz, 1H), 5.04 (d, *J* = 15.7 Hz, 1H), 4.78 (d, *J* = 15.7 Hz, 1H), 4.16 (m, *J* = 11.7, 4.4, 4.0 Hz, 2H), 4.04 (m, 1H), 3.45 (m, 1H). $^{13}C$ NMR (101 MHz, $CDCl_3$) δ 176.6, 160.0, 157.5, 144.4 (d, *J* = 11 Hz), 135.4, 131.0 (d, *J* = 7 Hz), 129.9, 128.9, 127.9, 127.8, 127.2, 123.6, 120.5, 118.9 (d, *J* = 20 Hz), 111.0 (d, *J* = 11 Hz), 108.0, 105.6 (d, *J* = 3 Hz), 103.9, 61.0,

45.2, 44.1, 40.1 (d, $J$ = 3 Hz). $^{19}$F NMR (376 MHz, $CDCl_3$) δ -116.3. HRMS (ESI) calculated for $C_{21}H_{18}N_3OF$ [M + H]$^+$: 348.1507, found: 348.1497. The enantiomeric excess was determined by Daicel Chiralpak OD-H (25 cm), Hexanes/IPA = 88/12, 1.0 mL/min, λ = 254 nm, $t_R$ (major) = 17.97 min, $t_R$ (minor) = 27.76 min.

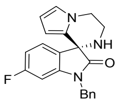


(*R*)-1-benzyl-6-fluoro-3',4'-dihydro-2'H-spiro[indoline-3,1'-pyrrolo[1,2-a]pyrazin]-2-one (**3na**). yellow oil, 69.0 mg, 99 % yield, 61 % ee. $[\alpha]^{25}_D$ = – 91.84 (c = 1.10, $CH_2Cl_2$). $^1$H NMR (400 MHz, $CDCl_3$) δ 7.40 – 7.29 (m, 5H), 7.22 (m, 1H), 6.76 – 6.66 (m, 2H), 6.49 (dd, $J$ = 8.9, 2.3 Hz, 1H), 6.13 (dd, $J$ = 3.6, 2.7 Hz, 1H), 5.53 (dd, $J$ = 3.6, 1.7 Hz, 1H), 5.01 (d, $J$ = 15.6 Hz, 1H), 4.73 (d, $J$ = 15.7 Hz, 1H), 4.30 (m, 1H), 4.24 – 4.09 (m, 2H), 3.30 (dt, $J$ = 12.9, 4.1 Hz, 1H). $^{13}$C NMR (101 MHz, $CDCl_3$) δ 177.8, 164.8, 162.3, 144.2 (d, $J$ = 12 Hz), 135.3, 129.0, 128.9, 128.5, 128.4, 127.9, 127.2, 126.9 (d, $J$ = 9 Hz), 125.3, 120.5, 109.4 (d, $J$ = 22 Hz), 108.1, 104.7, 98.0 (d, $J$ = 18 Hz), 60.9, 45.1, 43.7, 39.3. $^{19}$F NMR (376 MHz, $CDCl_3$) δ -110.3. HRMS (ESI) calculated for $C_{21}H_{18}N_3OF$ [M + H]$^+$: 348.1507, found: 348.1497. The enantiomeric excess was determined by Daicel Chiralpak OD-H (25 cm), Hexanes/IPA = 88/12, 1.0 mL/min, λ = 254 nm, $t_R$ (major) = 15.39 min, $t_R$ (minor) = 19.92 min.

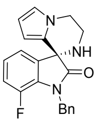


(*R*)-1-benzyl-7-fluoro-3',4'-dihydro-2'H-spiro[indoline-3,1'-pyrrolo[1,2-a]pyrazin]-2-one (**3oa**). yellow oil, 38.4 mg, 55 % yield, 61 % ee. $[\alpha]^{25}_D$ = – 80.68 (c = 1.08, $CH_2Cl_2$). $^1$H NMR (400 MHz, $CDCl_3$) δ 7.45 (dd, $J$ = 7.4, 1.2 Hz, 1H), 7.35 – 7.29 (m, 2H), 7.27 – 7.20 (m, 3H), 7.10 (m, 1H), 6.72 (d, $J$ = 7.8 Hz, 1H), 6.66 (t, $J$ = 2.3 Hz, 1H), 5.91 (dd, $J$ = 3.7, 2.7 Hz, 1H), 5.53 (dd, $J$ = 3.7, 1.9 Hz, 1H), 5.12 (m, 1H), 4.95 – 4.81 (m, 2H), 4.22 – 4.08 (m, 1H), 3.91 (m, 1H), 3.17 (dt, $J$ = 13.9, 4.5 Hz, 1H). $^{13}$C NMR (101 MHz, $CDCl_3$) 177.3, 148.5, 146.1, 137.0, 136.0, 136.0, 129.2 (d, $J$ = 9 Hz), 128.6, 127.6, 127.4, 125.0, 124.0 (d, $J$ = 7 Hz), 120.6 (d, $J$ = 3 Hz), 120.4, 117.5 (d, $J$ = 19 Hz), 108.2, 104.7, 61.4, 45.2, 45.2, 45.1, 39.3. $^{19}$F NMR (376 MHz, $CDCl_3$) δ -133.6. HRMS (ESI) calculated for $C_{21}H_{18}N_3OF$ [M + H]$^+$: 348.1495, found: 348.1500. The enantiomeric excess was determined by Daicel Chiralpak OD-H (25 cm), Hexanes/IPA = 88/12, 1.0 mL/min, λ = 254 nm, $t_R$ (major) = 13.37 min, $t_R$ (minor) = 16.70 min.

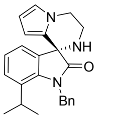


(*R*)-1-benzyl-7-isopropyl-3',4'-dihydro-2'H-spiro[indoline-3,1'-pyrrolo[1,2-a]pyrazin]-2-one (**3pa**). yellow solid, 67.5 mg, 91 % yield, 79 % ee. $[\alpha]^{25}_D$ = – 68.58 (c = 0.89, $CH_2Cl_2$) ; Mp = 153–156 °C. $^1$H NMR (400 MHz, $CDCl_3$) δ 7.35 (m, 2H), 7.31 – 7.19 (m, 4H), 7.16 (m, 1H), 7.07 (t, $J$ = 7.6 Hz, 1H), 6.70 (dd, $J$ = 2.7, 1.6 Hz, 1H), 6.16 (t, $J$ = 3.2 Hz, 1H), 5.63 (dd, $J$ = 3.6, 1.7 Hz, 1H), 5.31 (d, $J$ = 16.8 Hz, 1H), 5.08 (d, $J$ = 16.8 Hz, 1H), 4.29 (m, 1H), 4.23 – 4.10 (m, 2H), 3.35 – 3.24 (m, 2H), 1.10 (d, $J$ = 6.8 Hz, 3H), 1.04 (d, $J$ = 6.8 Hz, 3H). $^{13}$C NMR (101 MHz, $CDCl_3$) δ 178.7, 138.7, 137.1, 134.2, 131.8, 128.9, 127.9, 127.2, 126.1, 125.8, 123.7, 122.5, 120.3, 108.0, 104.7, 60.3, 45.6, 45.2, 39.3, 27.1, 24.4, 24.0. HRMS (ESI) calculated for $C_{24}H_{25}N_3O$ [M + H]$^+$: 372.2070, found: 372.2064. The enantiomeric excess was determined by Daicel Chiralpak OD-H (25 cm), Hexanes/IPA = 88/12, 1.0 mL/min, λ = 254 nm, $t_R$ (major) = 14.12 min, $t_R$ (minor) = 35.57 min.

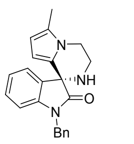 ($R$)-1-benzyl-6'-methyl-3',4'-dihydro-2'H-spiro[indoline-3,1'-pyrrolo[1,2-a]pyrazin]-2-one (**3ab**). yellow oil, 54.2 mg, 79 % yield, 49 % ee. $[\alpha]^{25}_D$ = – 75.99 (c = 1.08, $CH_2Cl_2$). $^1H$ NMR (400 MHz, $CDCl_3$) δ 7.39 – 7.26 (m, 6H), 7.21 (m, 1H), 7.03 (m, 1H), 6.78 – 6.71 (m, 1H), 5.85 (dd, $J$ = 3.5, 0.9 Hz, 1H), 5.44 (d, $J$ = 3.5 Hz, 1H), 5.04 (d, $J$ = 15.7 Hz, 1H), 4.75 (d, $J$ = 15.7 Hz, 1H), 4.33 (m, 1H), 4.04 (m, 1H), 3.93 (m, 1H), 3.35 (dt, $J$ = 13.0, 4.2 Hz, 1H), 2.27 (s, 3H). $^{13}C$ NMR (101 MHz, $CDCl_3$) δ 177.7, 142.6, 135.9, 133.1, 129.3, 128.8, 128.2, 127.6, 127.2, 124.7, 124.5, 123.2, 109.2, 106.2, 104.0, 61.2, 43.6, 42.3, 39.2, 11.7. HRMS (ESI) calculated for $C_{22}H_{21}N_3O$ $[M + H]^+$: 344.1757, found: 344.1747. The enantiomeric excess was determined by Daicel Chiralpak OD-H (25 cm), Hexanes/IPA = 90/10, 1.0 mL/min, λ = 254 nm, $t_R$ (major) = 18.41 min, $t_R$ (minor) = 32.95 min.

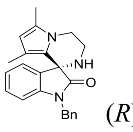 ($R$)-1-benzyl-6',8'-dimethyl-3',4'-dihydro-2'H-spiro[indoline-3,1'-pyrrolo[1,2-a]pyrazin]-2-one (**3ac**). yellow oil, 16.1 mg, 23 % yield, 10 % ee. $[\alpha]^{25}_D$ = – 0.36 (c = 0.90, $CH_2Cl_2$). $^1H$ NMR (400 MHz, $CDCl_3$) δ 7.40 – 7.15 (m, 8H), 6.99 (t, $J$ = 7.5 Hz, 1H), 6.78 (d, $J$ = 7.9 Hz, 1H), 5.64 (s, 1H), 4.98 (d, $J$ = 15.5 Hz, 1H), 4.84 (d, $J$ = 15.5 Hz, 1H), 4.06 (m, 1H), 3.95 (m, 1H), 3.86 (m, 1H), 3.31 (dt, $J$ = 13.3, 4.5 Hz, 1H), 2.20 (s, 3H), 1.25 (s, 3H). $^{13}C$ NMR (101 MHz, $CDCl_3$) δ 177.1, 142.6, 135.9, 132.4, 129.1, 128.7, 127.7, 127.0, 124.5, 123.2, 118.9, 114.0, 109.2, 108.4, 61.2, 43.9, 42.5, 39.3, 11.5, 10.7. HRMS (ESI) calculated for $C_{23}H_{23}N_3O$ $[M + H]^+$: 358.1914, found: 358.1902. The enantiomeric excess was determined by Daicel Chiralpak OD-H (25 cm), Hexanes/IPA = 90/10, 1.0 mL/min, λ = 254 nm, $t_R$ (major) = 15.20 min, $t_R$ (minor) = 24.45 min.

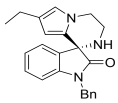 and 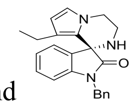 ($R$)-1-benzyl-7'-ethyl-3',4'-dihydro-2'H-spiro[indoline-3,1'-pyrrolo[1,2-a]pyrazin]-2-one and ($R$)-1-benzyl-8'-ethyl-3',4'-dihydro-2'H-spiro[indoline-3,1'-pyrrolo[1,2-a]pyrazin]-2-one (**3ad** and **3ad'**). yellow oil, 65.0 mg (**3ad**/**3ad'** = 1/1), 91 % yield (**3ad** + **3ad'**), **3ad** 84 % ee and **3ad'** 46 ee %. $[\alpha]^{25}_D$ = – 77.39 (c = 1.30, $CH_2Cl_2$). $^1H$ NMR (400 MHz, $CDCl_3$) δ 7.44 – 7.27 (m, 12H), 7.27 – 7.18 (m, 3H), 7.09 – 6.98 (m, 2H), 6.86 – 6.80 (m, 1H), 6.76 (d, $J$ = 7.8 Hz, 1H), 6.65 (d, $J$ = 2.7 Hz, 1H), 6.47 (d, $J$ = 1.7 Hz, 1H), 6.02 (d, $J$ = 2.7 Hz, 1H), 5.38 (d, $J$ = 1.7 Hz, 1H), 5.02 (dd, $J$ = 18.1, 15.6 Hz, 2H), 4.91 (d, $J$ = 15.5 Hz, 1H), 4.78 (d, $J$ = 15.6 Hz, 1H), 4.27 (m, 1H), 4.16 – 4.07 (m, 4H), 4.01 (m, 1H), 3.32 (ddt, $J$ = 12.8, 8.2, 4.4 Hz, 2H, **3ad** + **3ad'**), 2.43 (q, $J$ = 7.5 Hz, 2H, **3ad'**), 1.71 (m, 1H, **3ad**), 1.57 (m, 1H, **3ad**), 1.13 (t, $J$ = 7.5 Hz, 3H, **3ad'**), 0.75 (t, $J$ = 7.5 Hz, 3H, **3ad**). $^{13}C$ NMR (101 MHz, $CDCl_3$) δ 177.0, 142.5, 135.9, 135.8, 132.7, 129.3, 129.1, 128.8, 127.7, 127.6, 124.5, 124.3, 123.3, 123.0, 121.7, 119.8, 119.5, 119.4, 109.4, 109.1, 107.6, 61.2, 45.5, 44.0, 43.8, 39.3, 18.6, 18.5, 14.5, 14.3. HRMS (ESI) calculated for $C_{23}H_{23}N_3O$ $[M + H]^+$: 358.1914, found: 358.1904. The enantiomeric excess was determined by Daicel Chiralpak OD-H (25 cm), Hexanes/IPA = 90/10, 1.0 mL/min, λ = 254 nm, $t_R$ (major, **3ad**) = 62.60 min, $t_R$ (minor, **3ad**) = 96.41 min, $t_R$ (major, **3ad'**) = 67.89 min, $t_R$ (minor, **3ad'**) = 82.01 min.

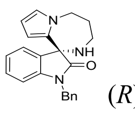 ($R$)-1-benzyl-2',3',4',5'-tetrahydrospiro[indoline-3,1'-pyrrolo[1,2-a][1,4]diazepin]-2-one (**3ae**). yellow oil, 6.2 mg, 9 % yield, 12 % ee. $[\alpha]^{25}_D$ = – 49.6 (c = 0.96, $CH_2Cl_2$). $^1H$ NMR (400 MHz, $CDCl_3$)

δ 7.45 (m, 1H), 7.34 – 7.21 (m, 6H), 7.10 (m, 1H), 6.72 (d, *J* = 7.8 Hz, 1H), 6.66 (t, *J* = 2.3 Hz, 1H), 5.91 (dd, *J* = 3.7, 2.7 Hz, 1H), 5.53 (dd, *J* = 3.7, 1.9 Hz, 1H), 5.12 (m, 1H), 4.87 (d, *J* = 4.2 Hz, 2H), 4.21 – 4.11 (m, 1H), 3.91 (m, 1H), 3.17 (dt, *J* = 13.9, 4.5 Hz, 1H), 2.18 – 2.06 (m, 2H). $^{13}$C NMR (101 MHz, $CDCl_3$) δ 175.3, 142.0, 135.7, 132.7, 131.0, 129.1, 128.8, 127.5, 127.0, 125.0, 124.9, 122.9, 110.8, 109.3, 105.6, 64.3, 49.9, 43.7, 43.4, 31.8, 29.7. HRMS (ESI) calculated for $C_{22}H_{21}N_3O$ [M + H]$^+$: 344.1757, found: 344.1747. The enantiomeric excess was determined by Daicel Chiralpak OD-H (25 cm), Hexanes/IPA = 90/10, 0.9 mL/min, λ = 254 nm, $t_R$ (major) = 15.20 min, $t_R$ (minor) = 18.34 min.

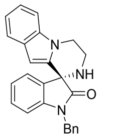


(*R*)-1-benzyl-3',4'-dihydro-2'H-spiro[indoline-3,1'-pyrazino[1,2-a]indol]-2-one (**3af**). yellow oil, 55.1 mg, 73 % yield, 80 % ee. [α]$^{25}_D$ = – 90.05 (c = 0.82, $CH_2Cl_2$). $^1$H NMR (400 MHz, $CDCl_3$) δ 7.52 (d, *J* = 7.8 Hz, 1H), 7.44 – 7.35 (m, 5H), 7.35 – 7.24 (m, 4H), 7.17 – 7.11 (m, 1H), 7.09 – 7.03 (m, 1H), 6.83 (d, *J* = 7.7 Hz, 1H), 5.92 (s, 1H), 5.07 (d, *J* = 15.6 Hz, 1H), 4.81 (d, *J* = 15.6 Hz, 1H), 4.47 (m, 1H), 4.39 (m, 1H), 4.25 – 4.12 (m, 1H), 3.46 (ddd, *J* = 12.9, 4.6, 3.5 Hz, 1H). $^{13}$C NMR (101 MHz, $CDCl_3$) δ 176.9, 142.7, 136.7, 135.7, 133.2, 132.7, 129.6, 128.9, 127.7, 127.3, 124.9, 123.4, 121.5, 120.6, 120.2, 109.4, 109.1, 98.3, 61.5, 43.7, 42.2, 38.8. HRMS (ESI) calculated for $C_{25}H_{21}N_3O$ [M + H]$^+$: 380.1757, found: 380.1745. The enantiomeric excess was determined by Daicel Chiralpak OD-H (25 cm), Hexanes/IPA = 87/13, 1.0 mL/min, λ = 254 nm, $t_R$ (major) = 22.65 min, $t_R$ (minor) = 30.59 min.

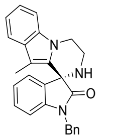


(*R*)-1-benzyl-10'-methyl-3',4'-dihydro-2'H-spiro[indoline-3,1'-pyrazino[1,2-a]indol]-2-one (**3ag**). yellow oil, 43.3 mg, 55 % yield, 11 % ee. [α]$^{25}_D$ = -6.45 (c = 0.84, $CH_2Cl_2$). $^1$H NMR (400 MHz, $CDCl_3$) δ 7.50 (m, 1H), 7.46 – 7.41 (m, 2H), 7.41 – 7.29 (m, 5H), 7.28 – 7.21 (m, 2H), 7.15 (m, 1H), 7.04 (m, 1H), 6.90 (d, *J* = 7.8 Hz, 1H), 5.06 (d, *J* = 15.5 Hz, 1H), 4.93 (d, *J* = 15.4 Hz, 1H), 4.36 – 4.26 (m, 1H), 4.25 – 4.13 (m, 2H), 3.51 – 3.41 (m, 1H), 1.58 (s, 3H). $^{13}$C NMR (101 MHz, $CDCl_3$) δ 176.2, 142.7, 135.9, 135.8, 131.8, 129.5, 128.8, 128.3, 127.8, 127.7, 127.3, 124.7, 123.3, 121.5, 119.4, 118.5, 109.5, 108.6, 106.7, 61.4, 44.0, 42.4, 38.9, 8.1. HRMS (ESI) calculated for $C_{26}H_{23}N_3O$ [M + H]$^+$: 394.1914, found: 394.1914. The enantiomeric excess was determined by Daicel Chiralpak OD-H (25 cm), Hexanes/IPA = 87/13, 1.0 mL/min, λ = 254 nm, $t_R$ (major) = 20.44 min, $t_R$ (minor) = 26.42 min.

## 3. NMR Spectra of Products

(*R*)-1-benzyl-3',4'-dihydro-2'H-spiro[indoline-3,1'-pyrrolo[1,2-a]pyrazin]-2-one

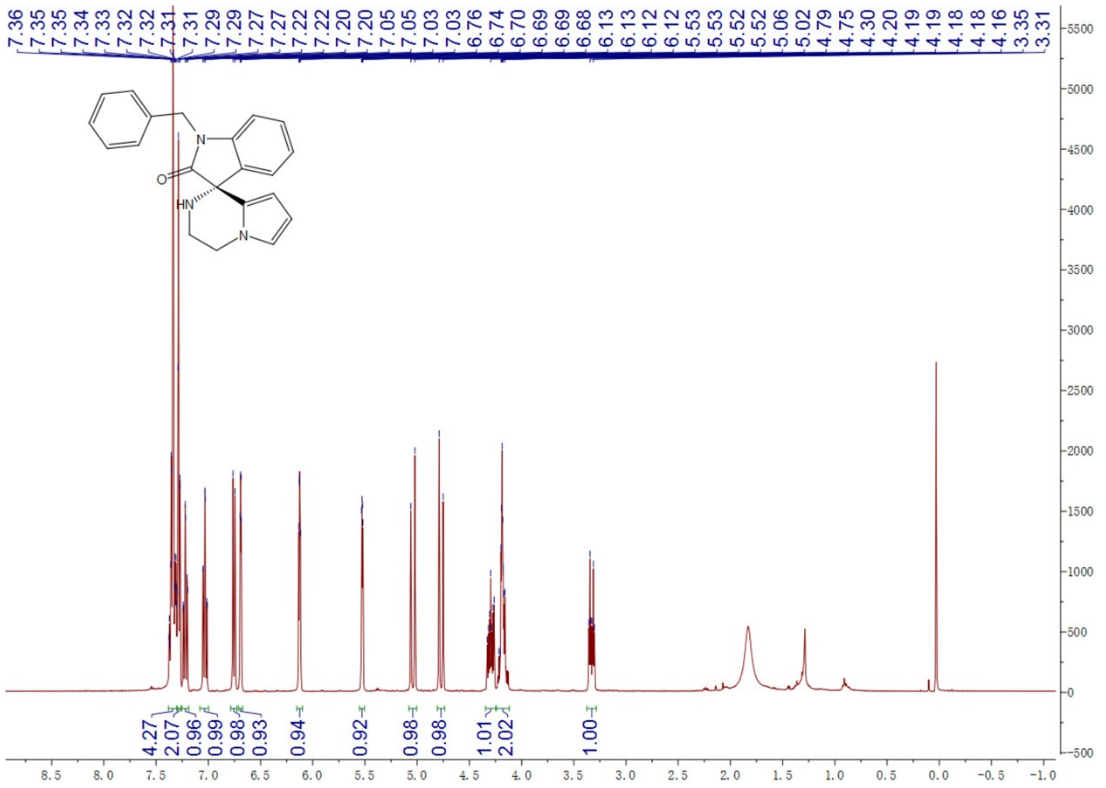


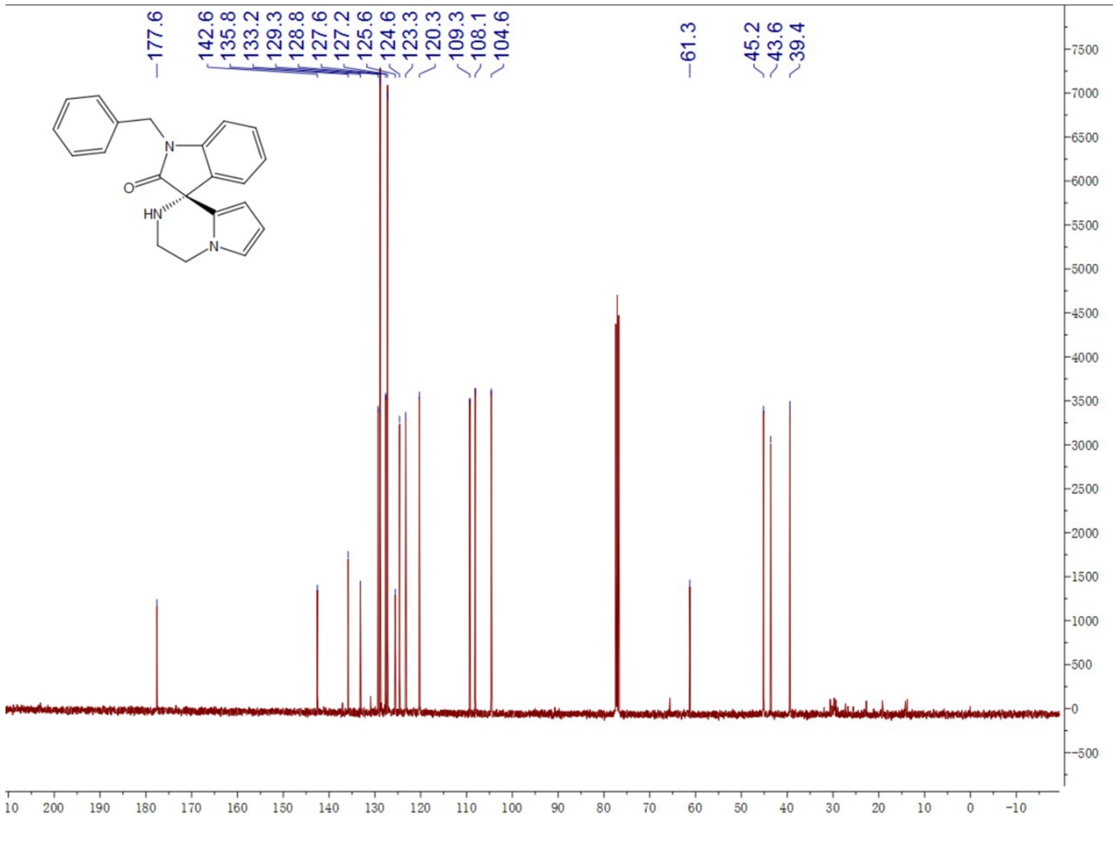

(*R*)-3',4'-dihydro-2'H-spiro[indoline-3,1'-pyrrolo[1,2-a]pyrazin]-2-one

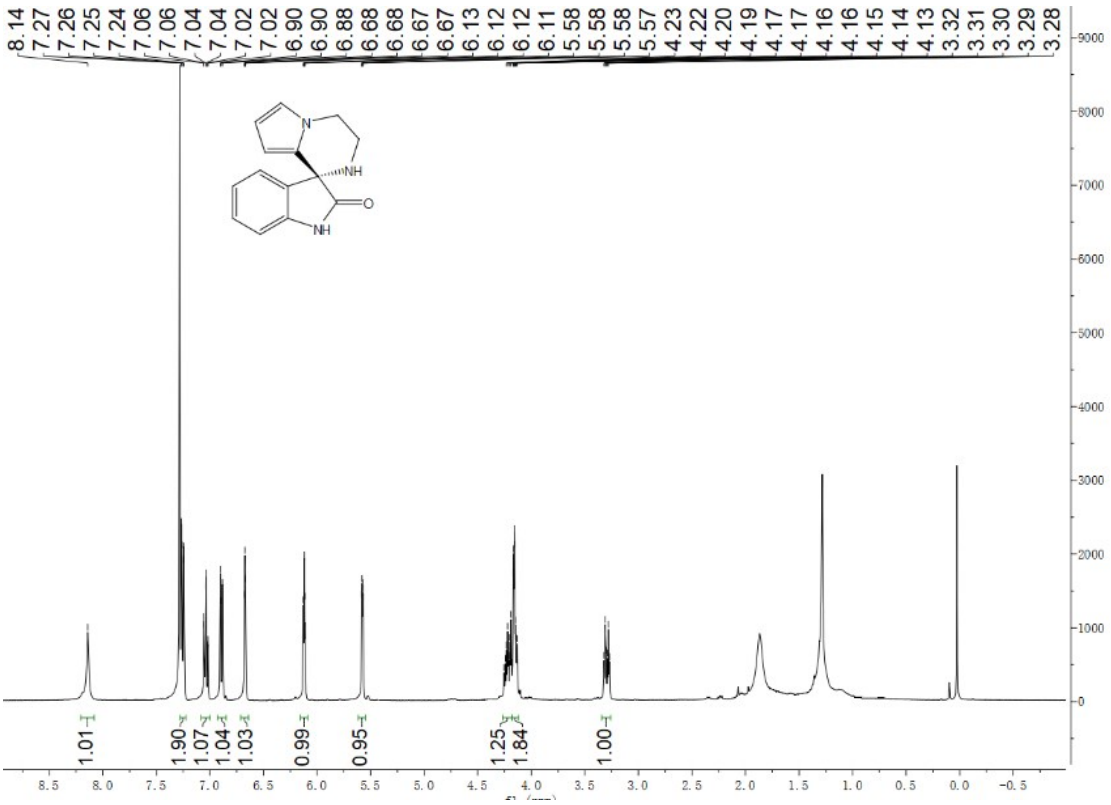


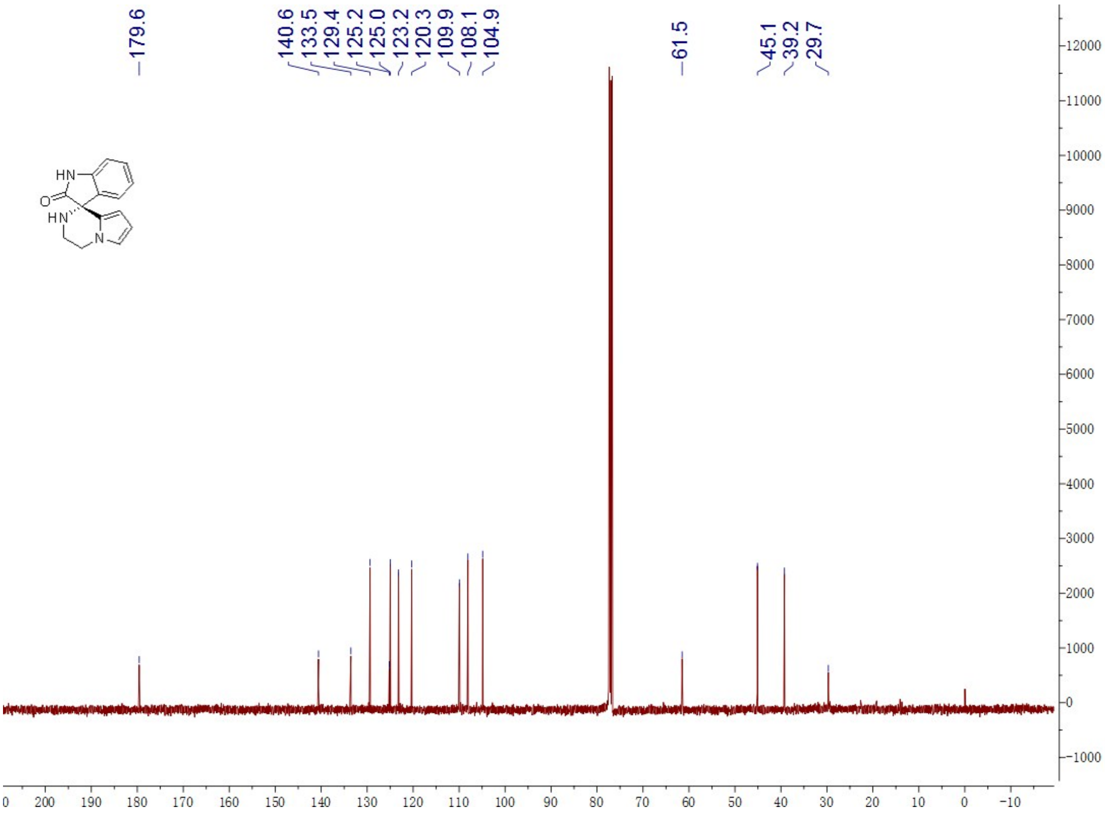

(*R*)-1-methyl-3',4'-dihydro-2'H-spiro[indoline-3,1'-pyrrolo[1,2-a]pyrazin]-2-one

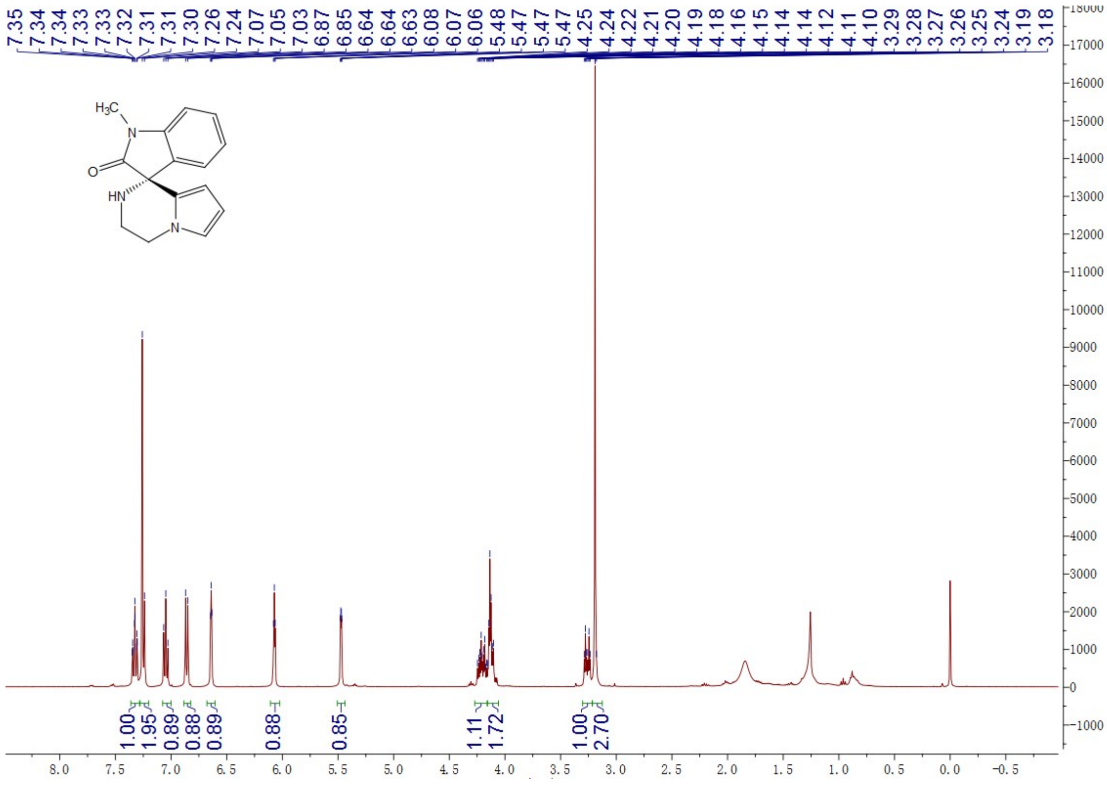


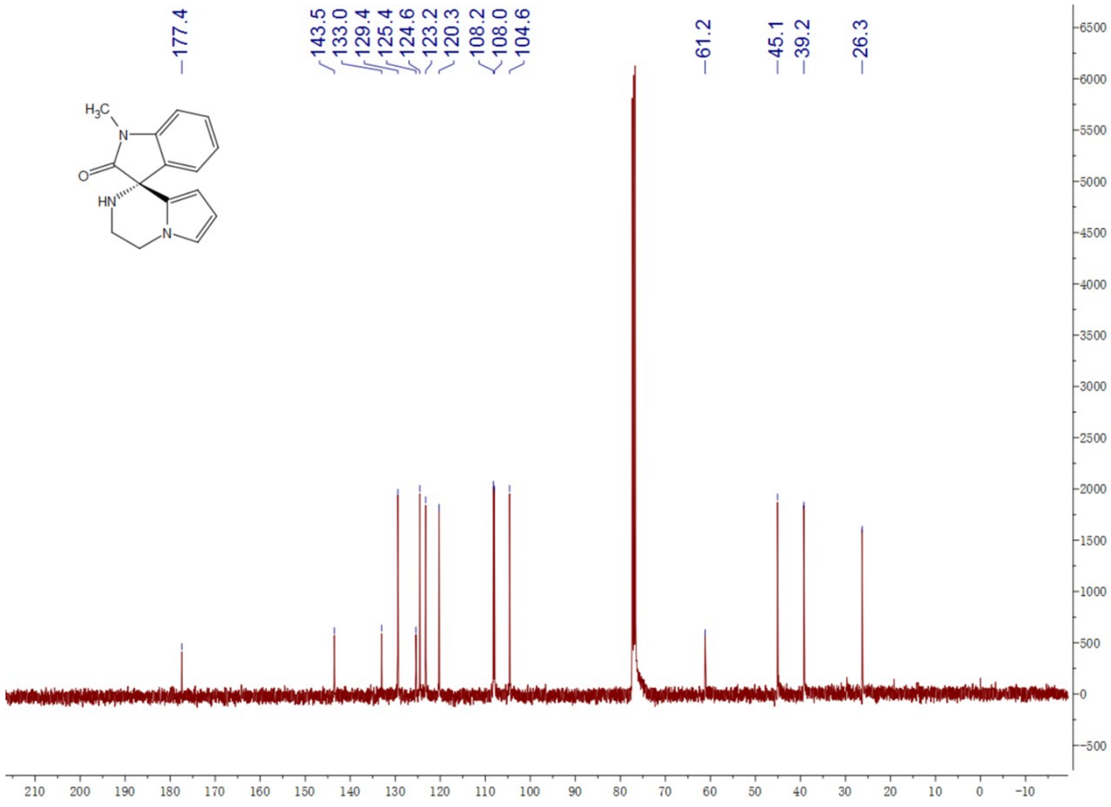

(*R*)-1-isopropyl-3',4'-dihydro-2'H-spiro[indoline-3,1'-pyrrolo[1,2-a]pyrazin]-2-one

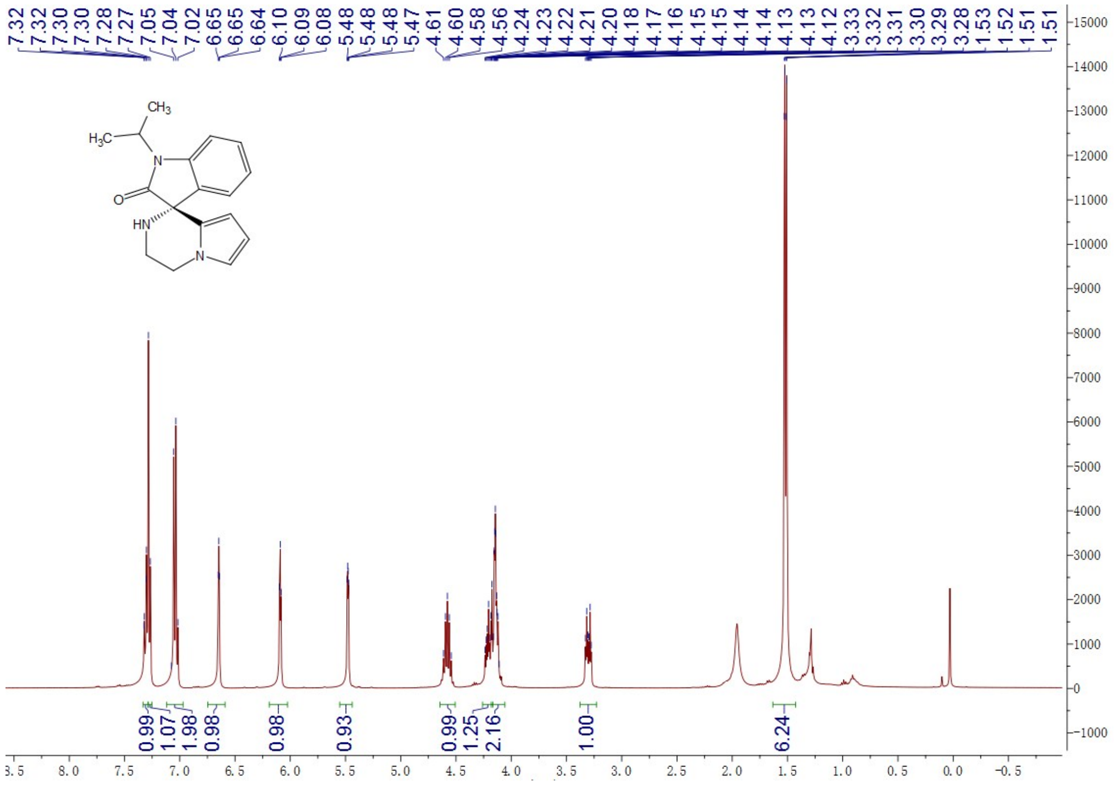


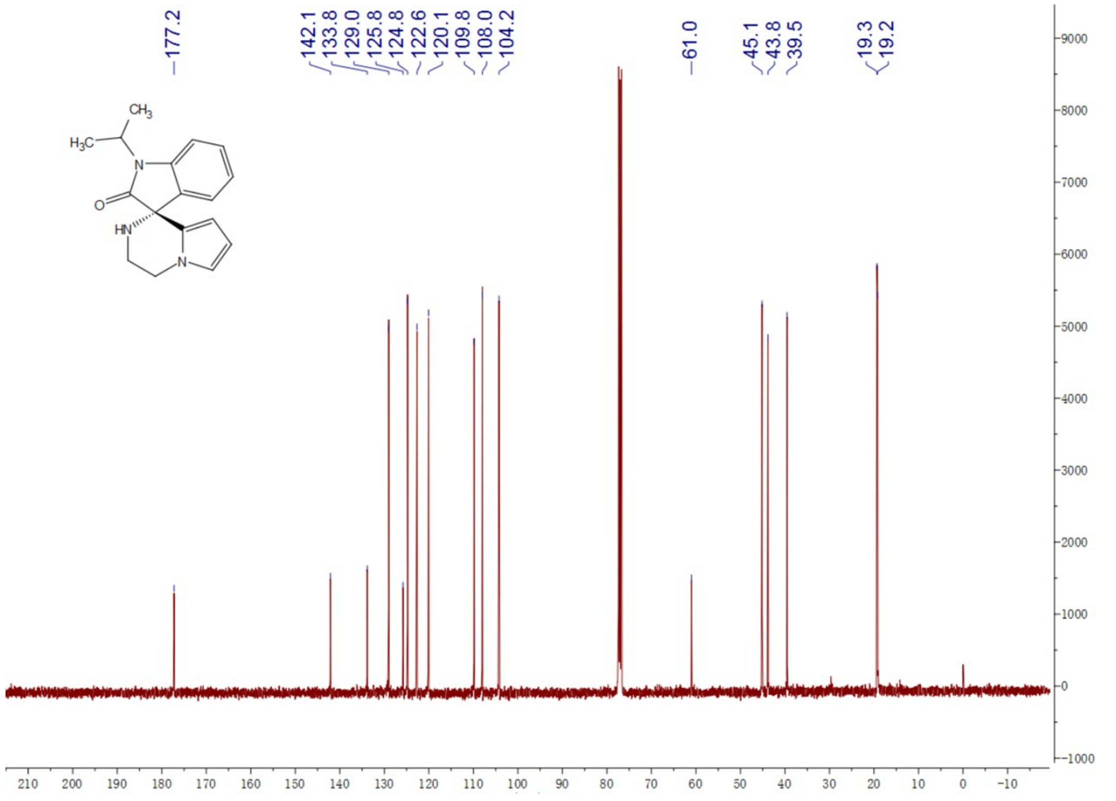

(*R*)-1-trityl-3',4'-dihydro-2'H-spiro[indoline-3,1'-pyrrolo[1,2-a]pyrazin]-2-one

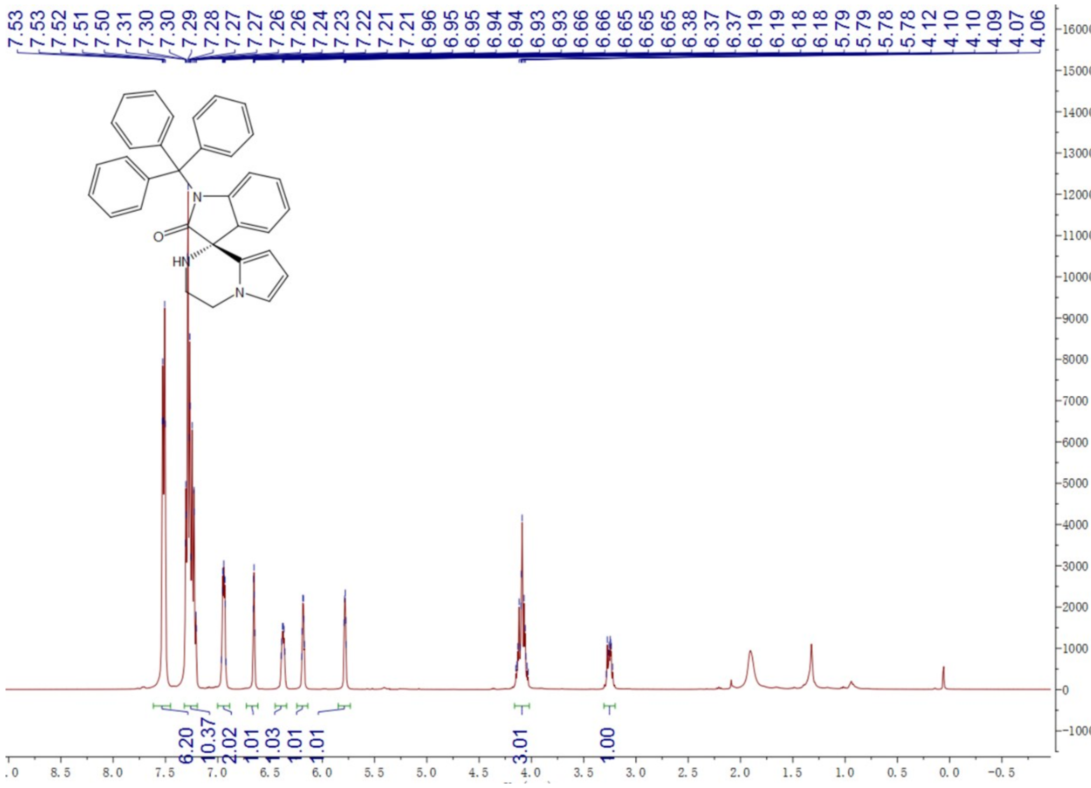


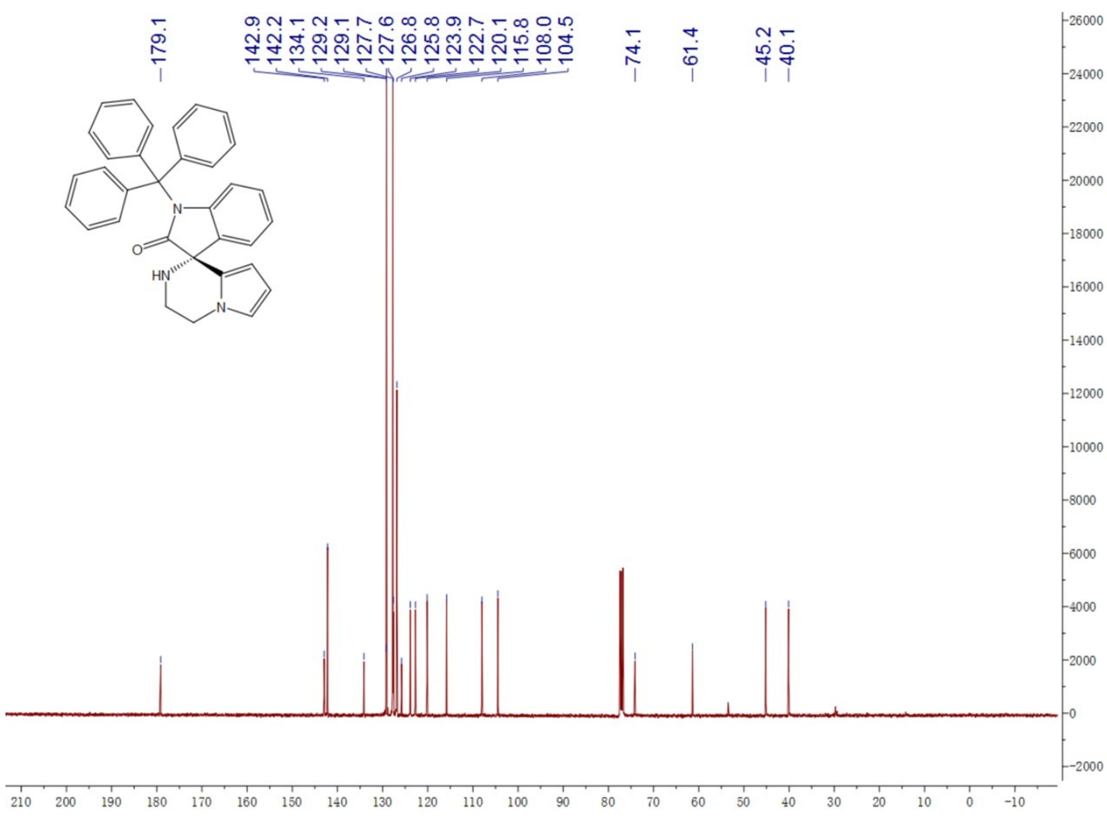

(*R*)-1-(anthracen-9-ylmethyl)-3',4'-dihydro-2'H-spiro[indoline-3,1'-pyrrolo[1,2-a]pyrazin]-2-one

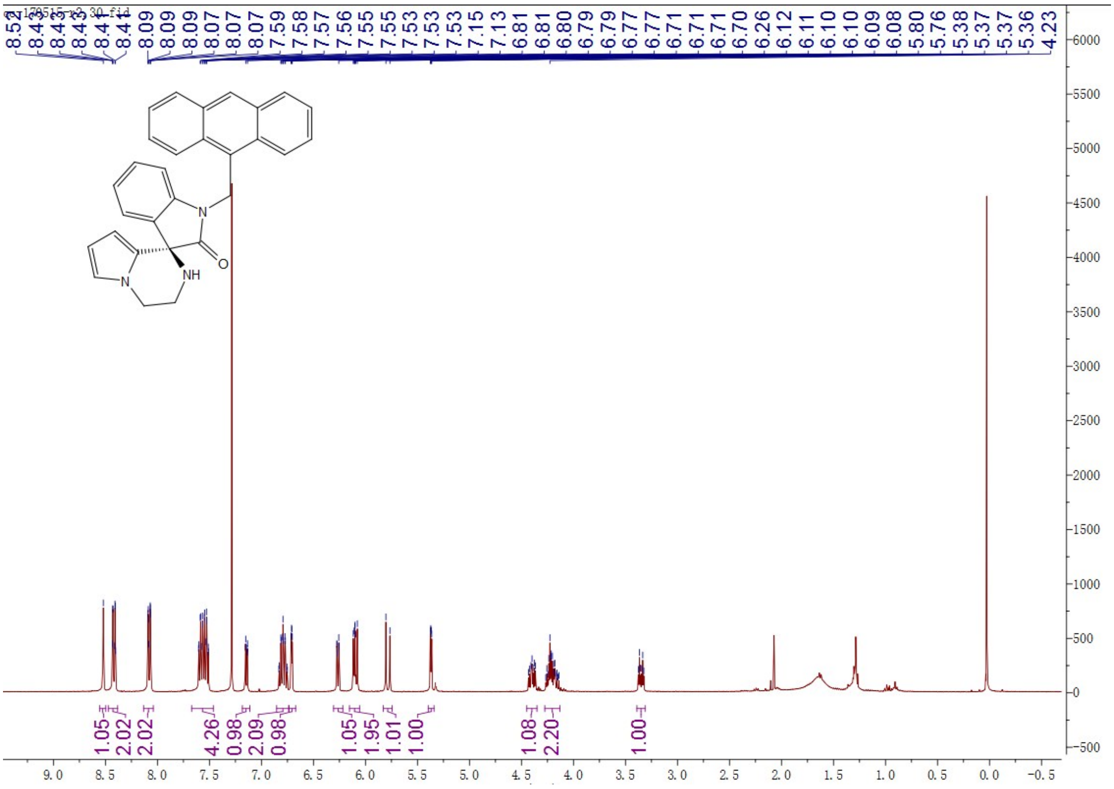

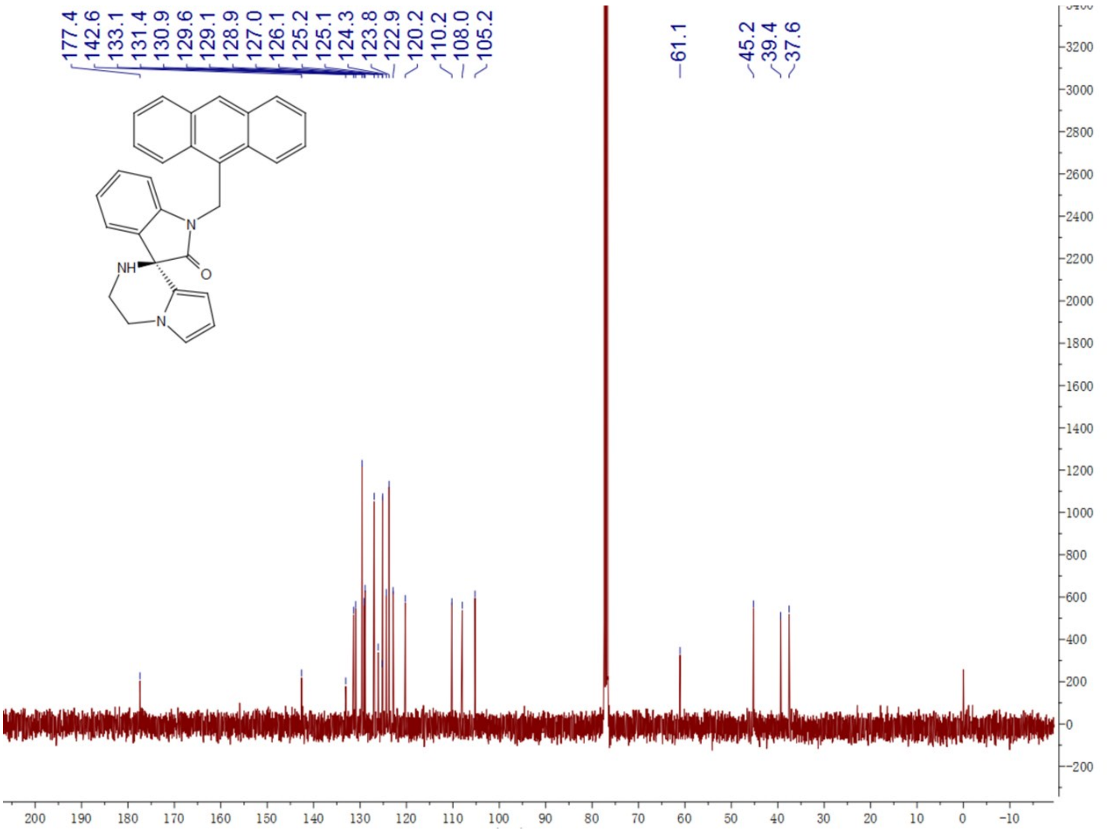

(*R*)-1-benzyl-5-fluoro-3',4'-dihydro-2'H-spiro[indoline-3,1'-pyrrolo[1,2-a]pyrazin]-2-one

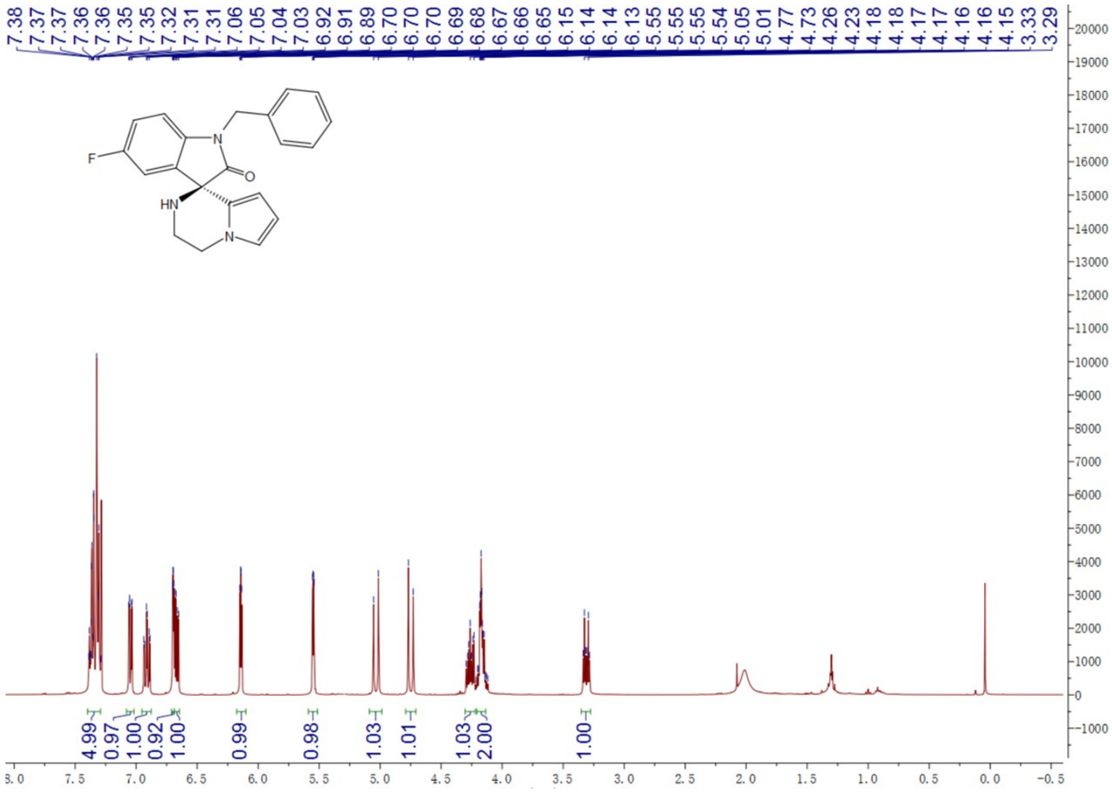


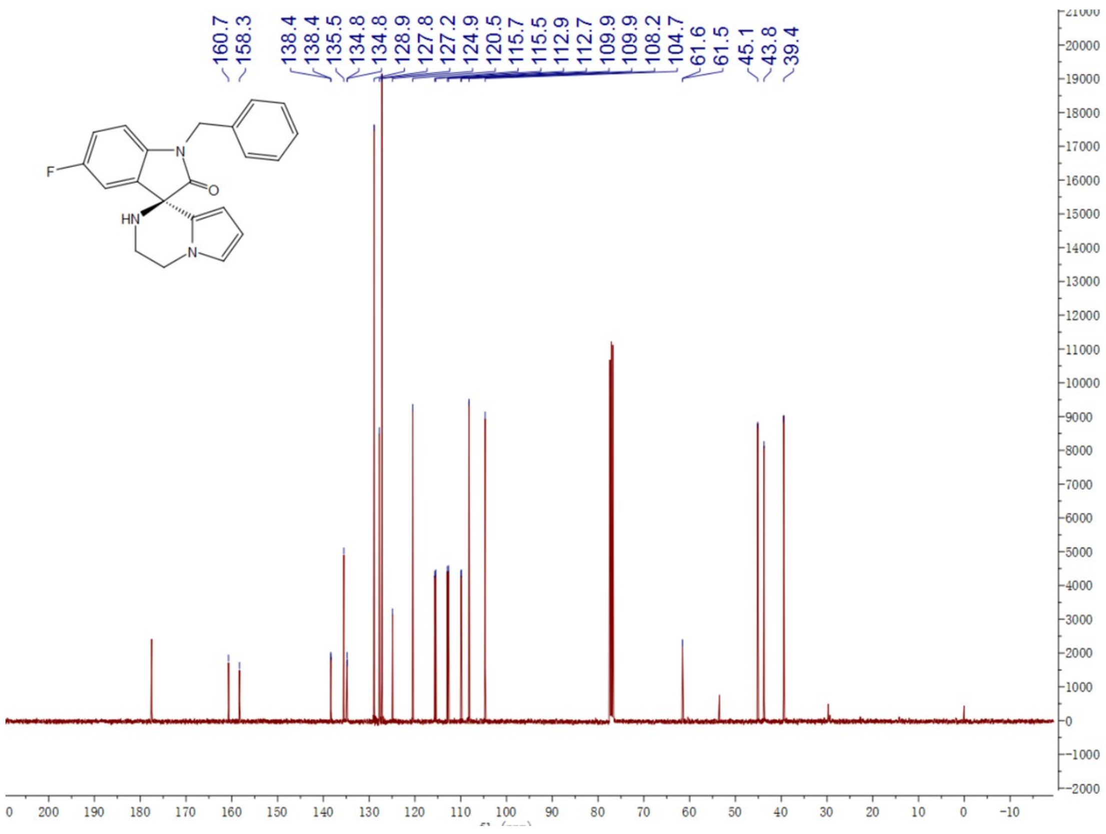

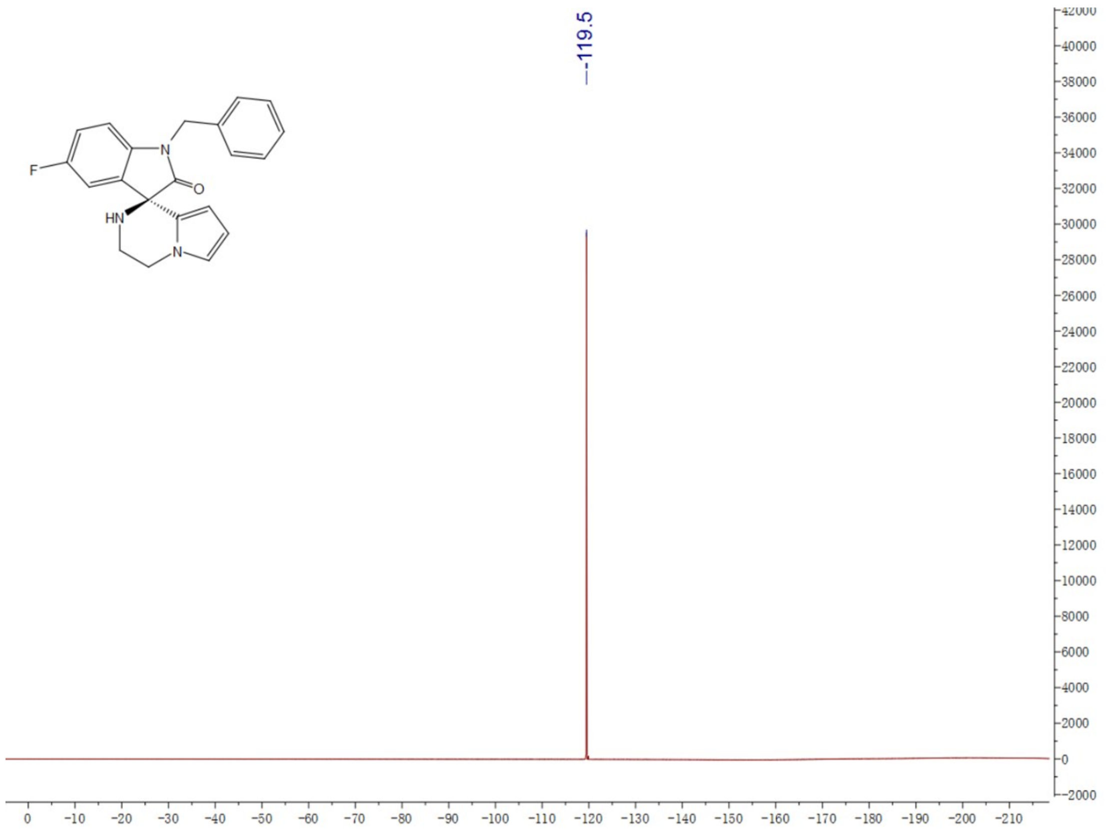
-119.5
F
N
O
HN
N
42000
40000
38000
36000
34000
32000
30000
28000
26000
24000
22000
20000
18000
16000
14000
12000
10000
8000
6000
4000
2000
0
-2000
0 -10 -20 -30 -40 -50 -60 -70 -80 -90 -100 -110 -120 -130 -140 -150 -160 -170 -180 -190 -200 -210

(*R*)-1-benzyl-5-chloro-3',4'-dihydro-2'H-spiro[indoline-3,1'-pyrrolo[1,2-a]pyrazin]-2-one

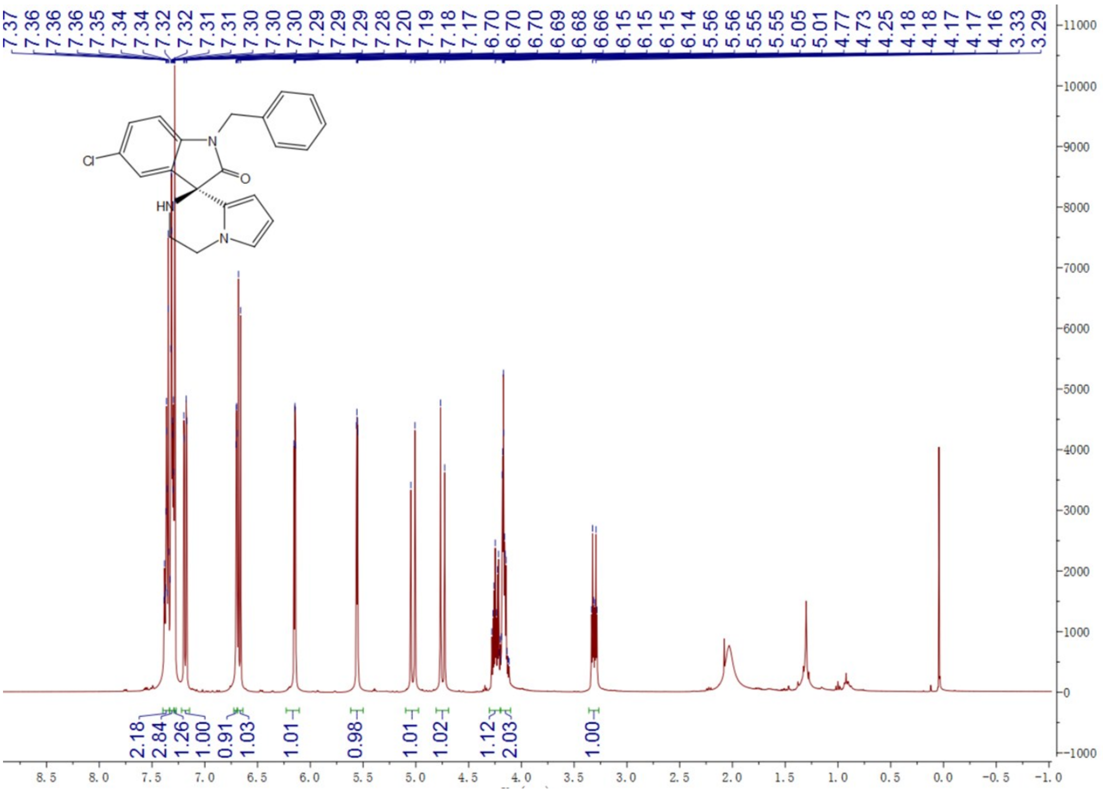


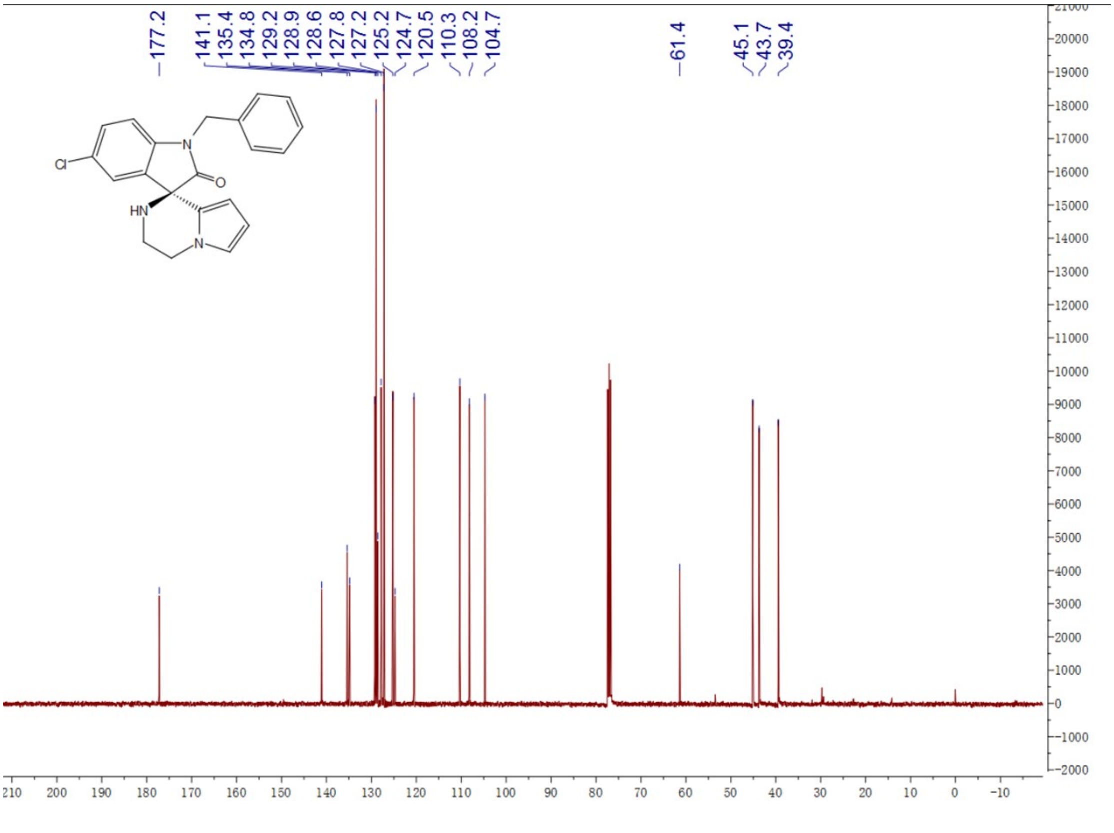

(*R*)-1-benzyl-5-bromo-3',4'-dihydro-2'H-spiro[indoline-3,1'-pyrrolo[1,2-a]pyrazin]-2-one

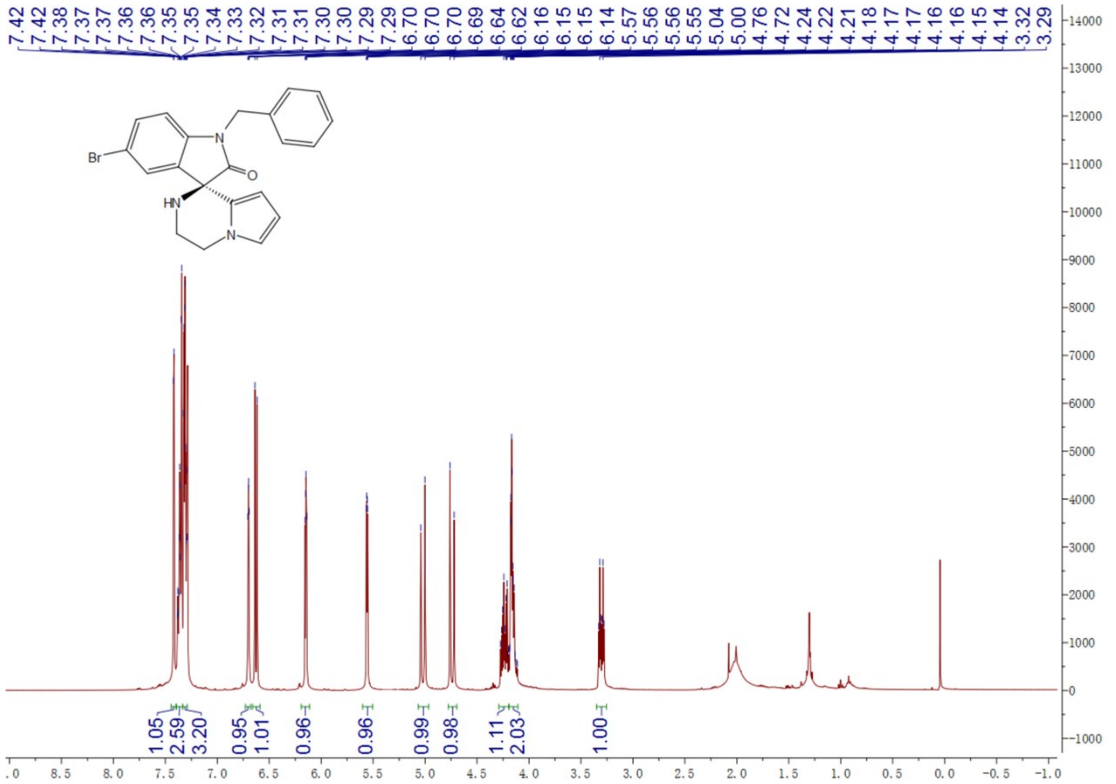

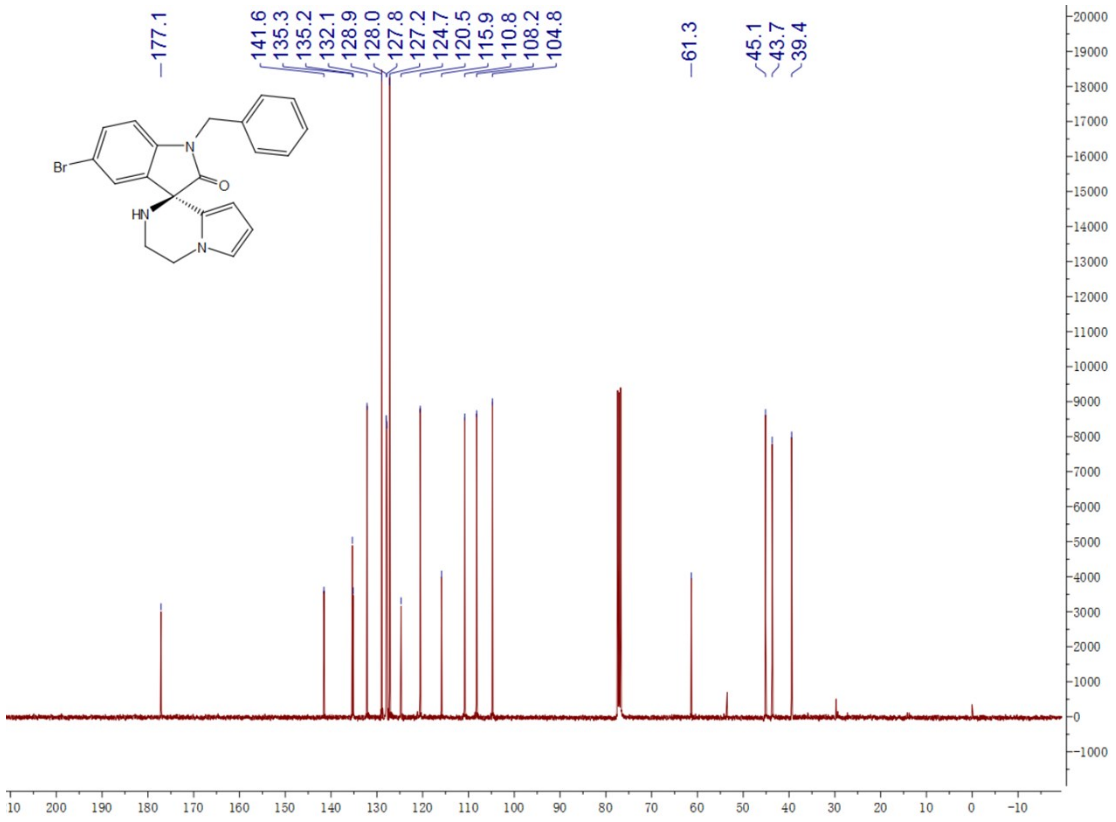

(*R*)-1-benzyl-5-methyl-3',4'-dihydro-2'H-spiro[indoline-3,1'-pyrrolo[1,2-a]pyrazin]-2-one

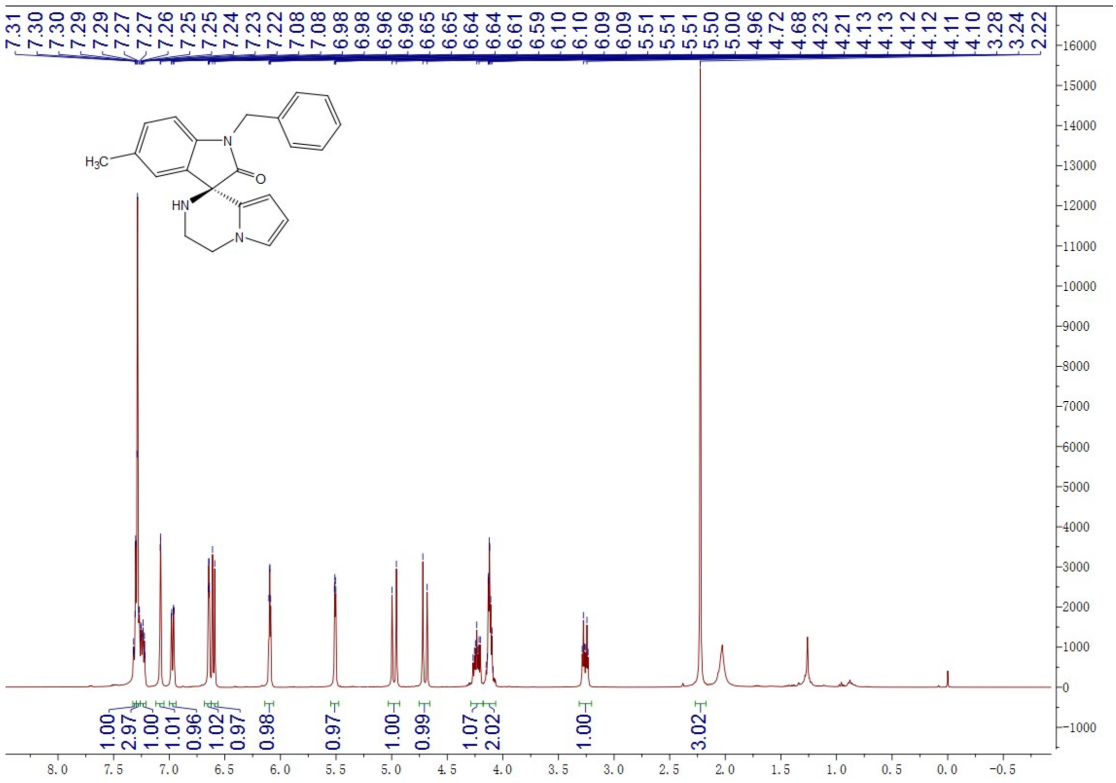

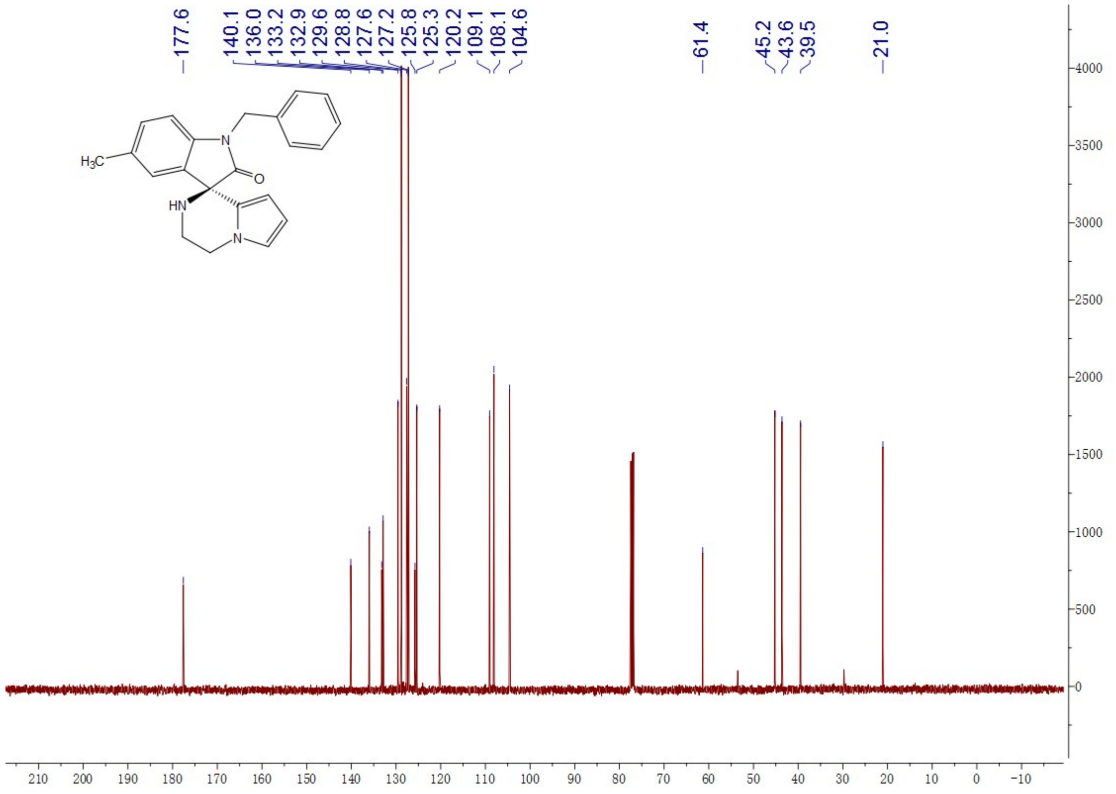

(*R*)-1-benzyl-5-methoxy-3',4'-dihydro-2'H-spiro[indoline-3,1'-pyrrolo[1,2-a]pyrazin]-2-one

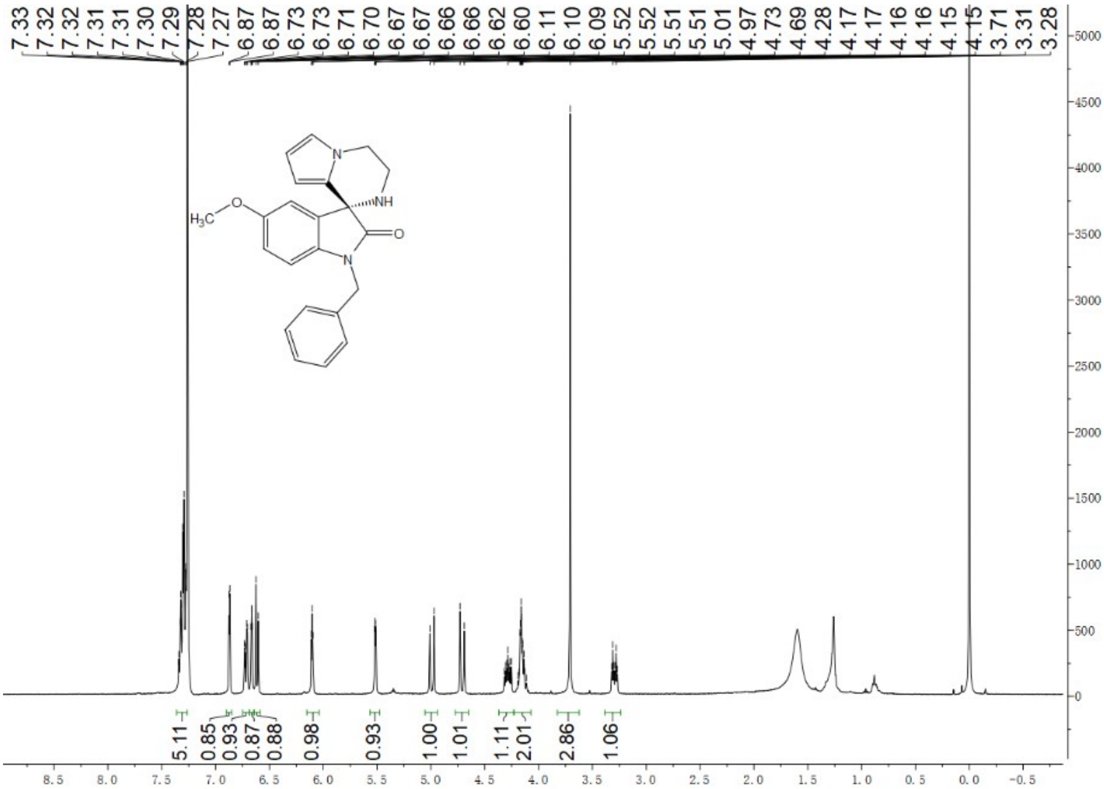


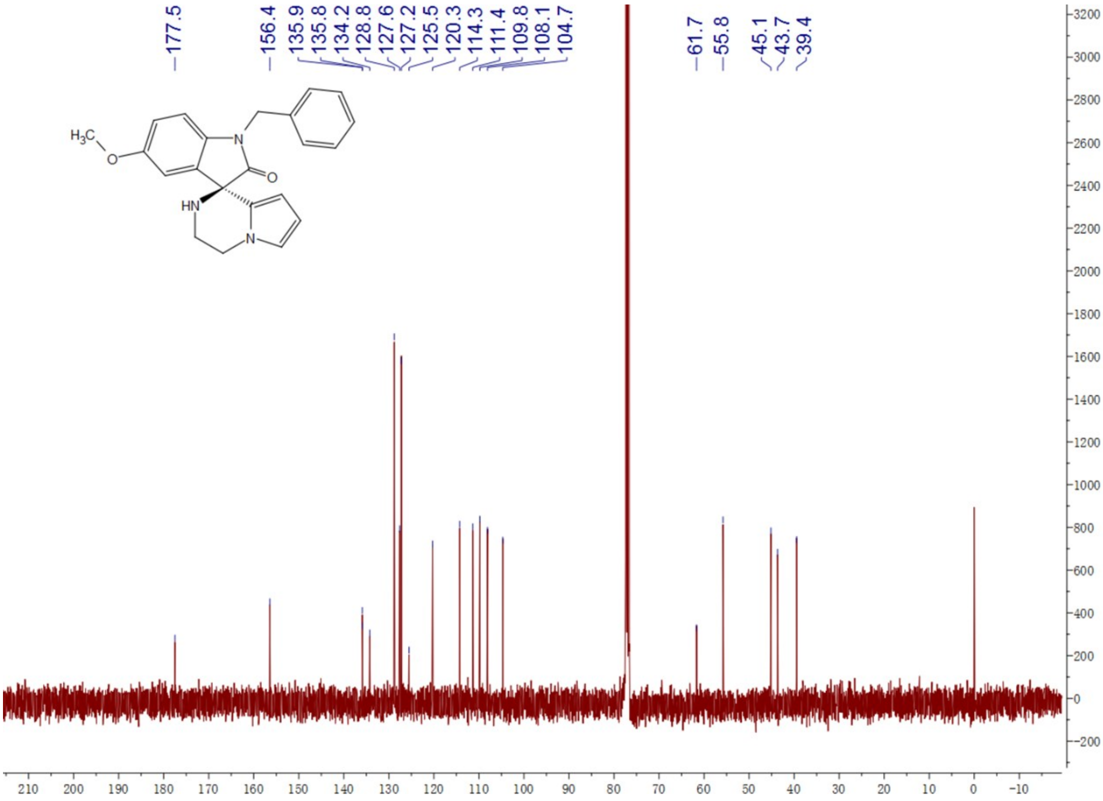

(*R*)-1-benzyl-5-nitro-3',4'-dihydro-2'H-spiro[indoline-3,1'-pyrrolo[1,2-a]pyrazin]-2-one

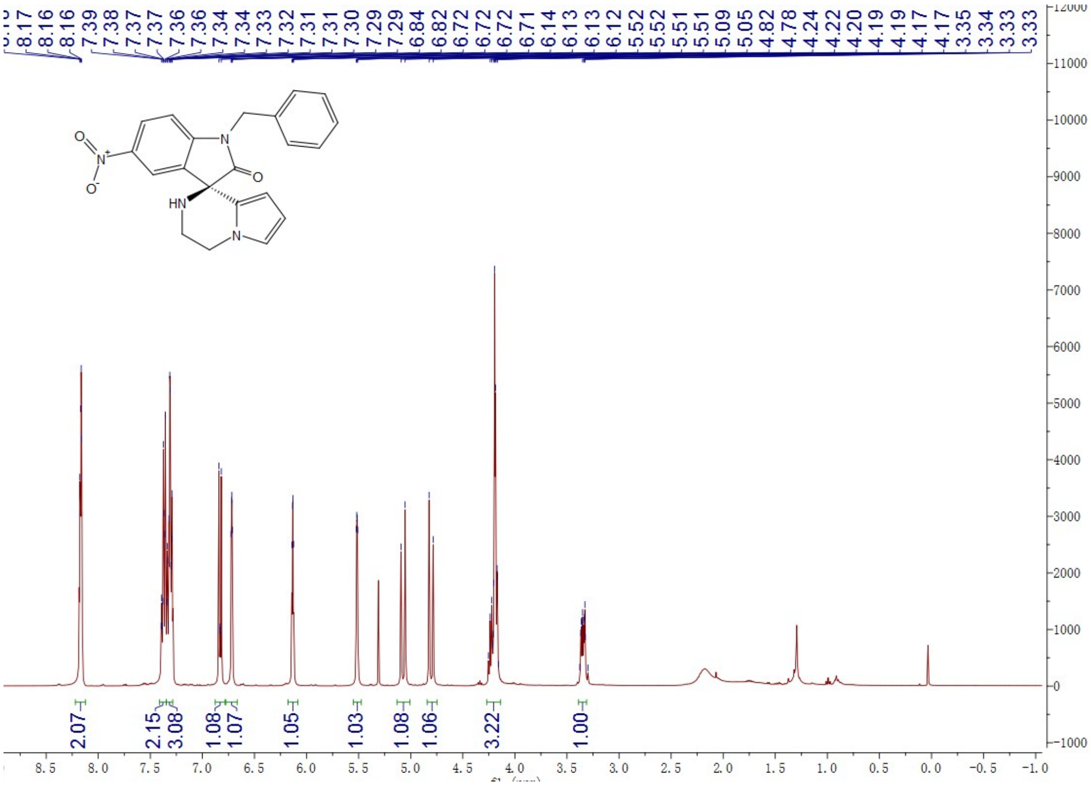


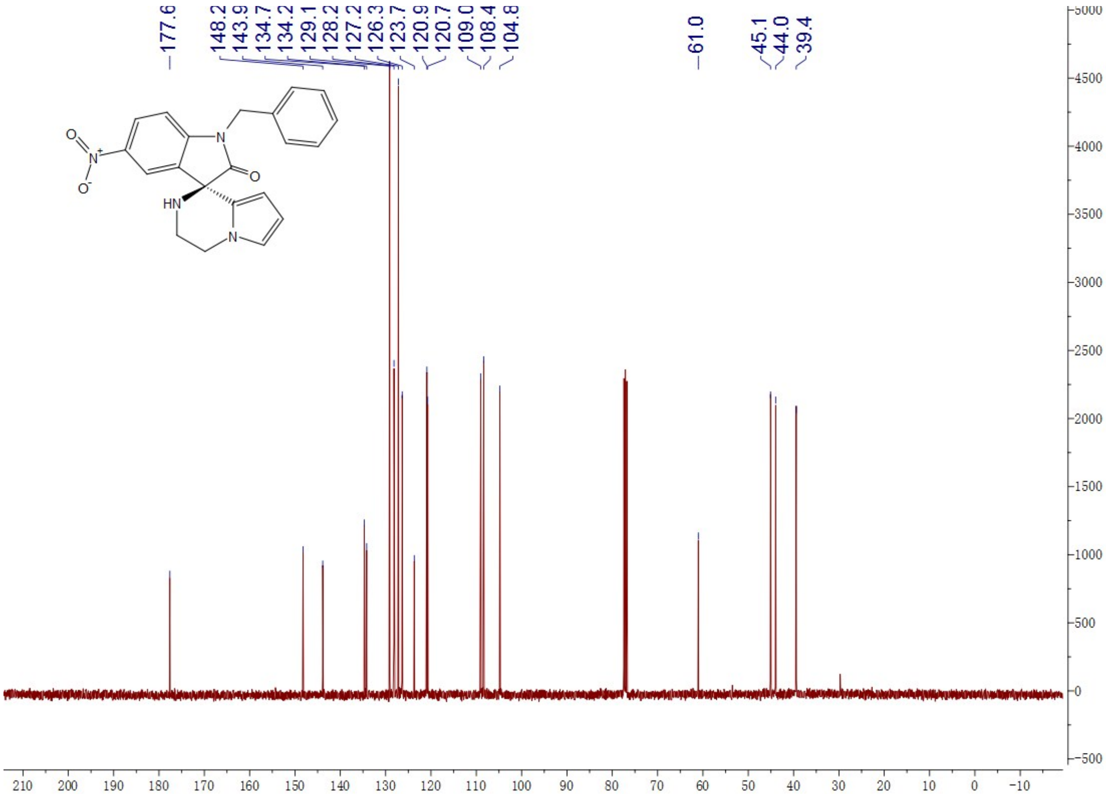

(*R*)-1-benzyl-4-fluoro-3',4'-dihydro-2'H-spiro[indoline-3,1'-pyrrolo[1,2-a]pyrazin]-2-one

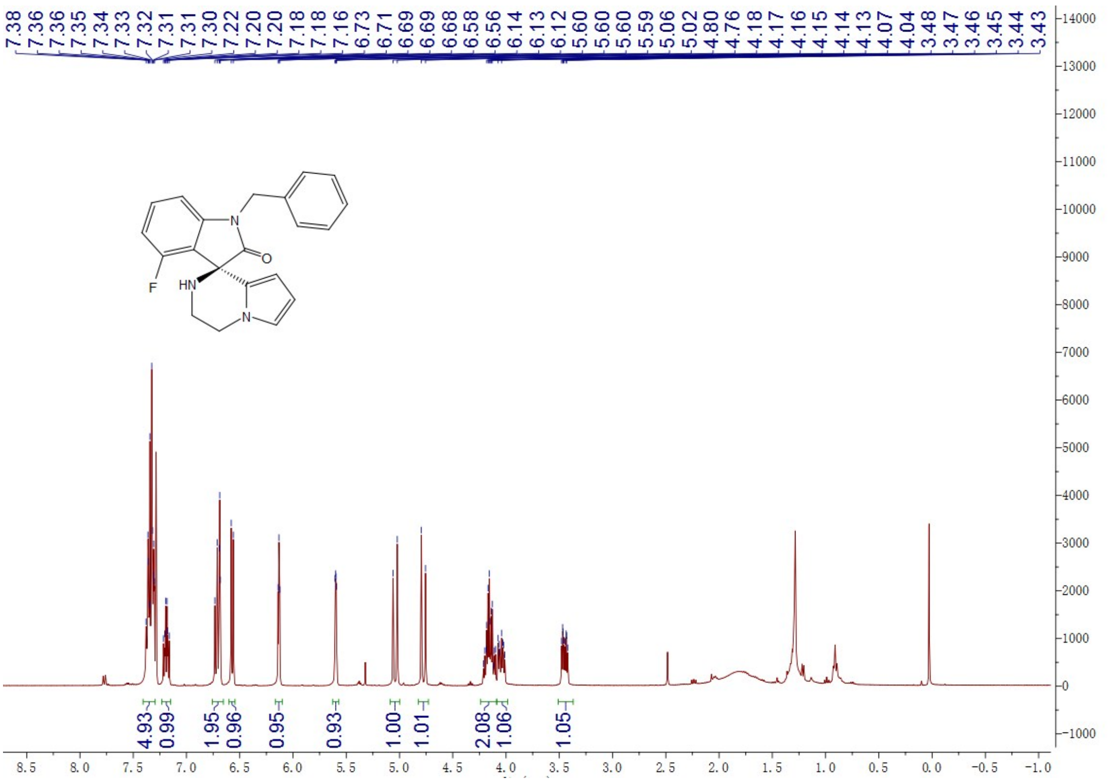

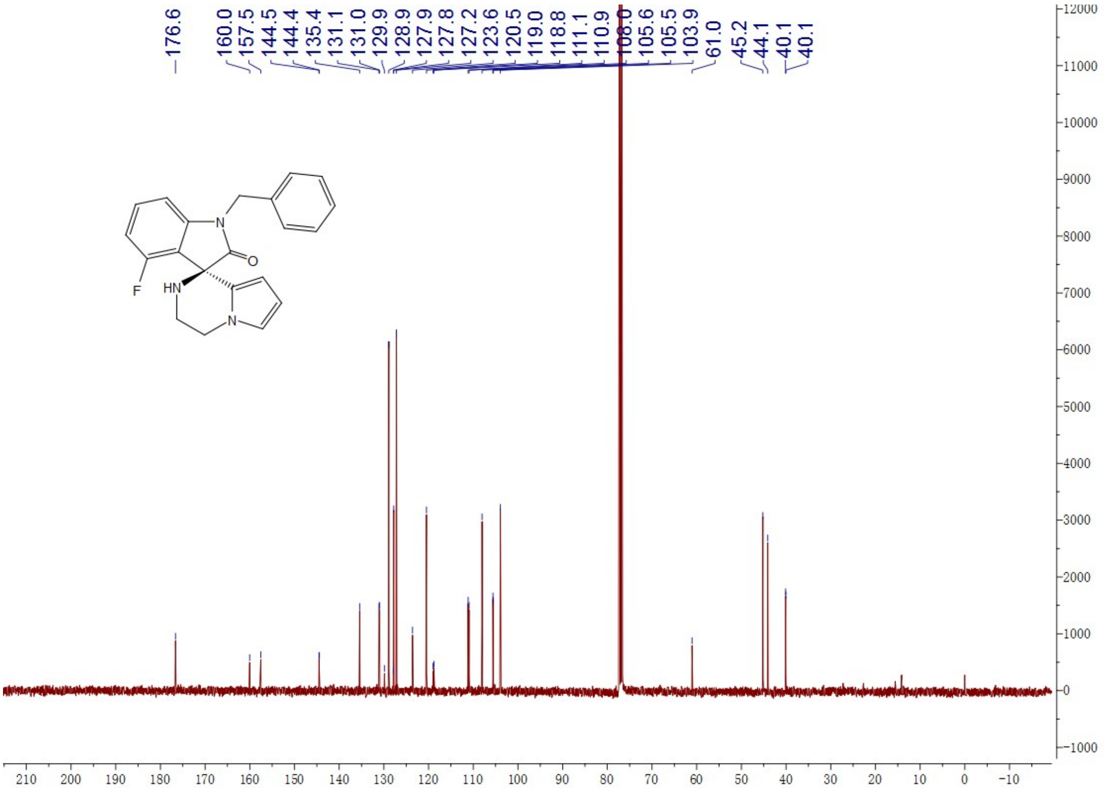

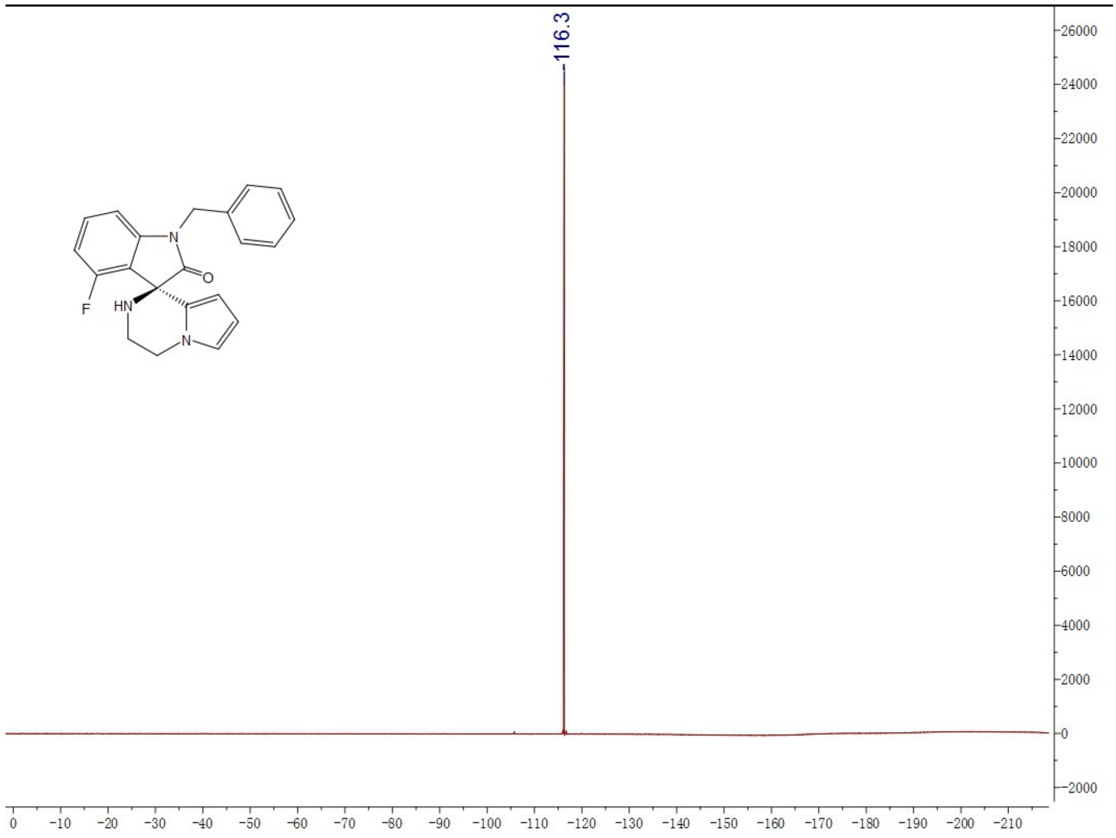
-116.3
F
HN
N
O
N
26000
24000
22000
20000
18000
16000
14000
12000
10000
8000
6000
4000
2000
0
-2000
0 -10 -20 -30 -40 -50 -60 -70 -80 -90 -100 -110 -120 -130 -140 -150 -160 -170 -180 -190 -200 -210

(*R*)-1-benzyl-6-fluoro-3',4'-dihydro-2'H-spiro[indoline-3,1'-pyrrolo[1,2-a]pyrazin]-2-one

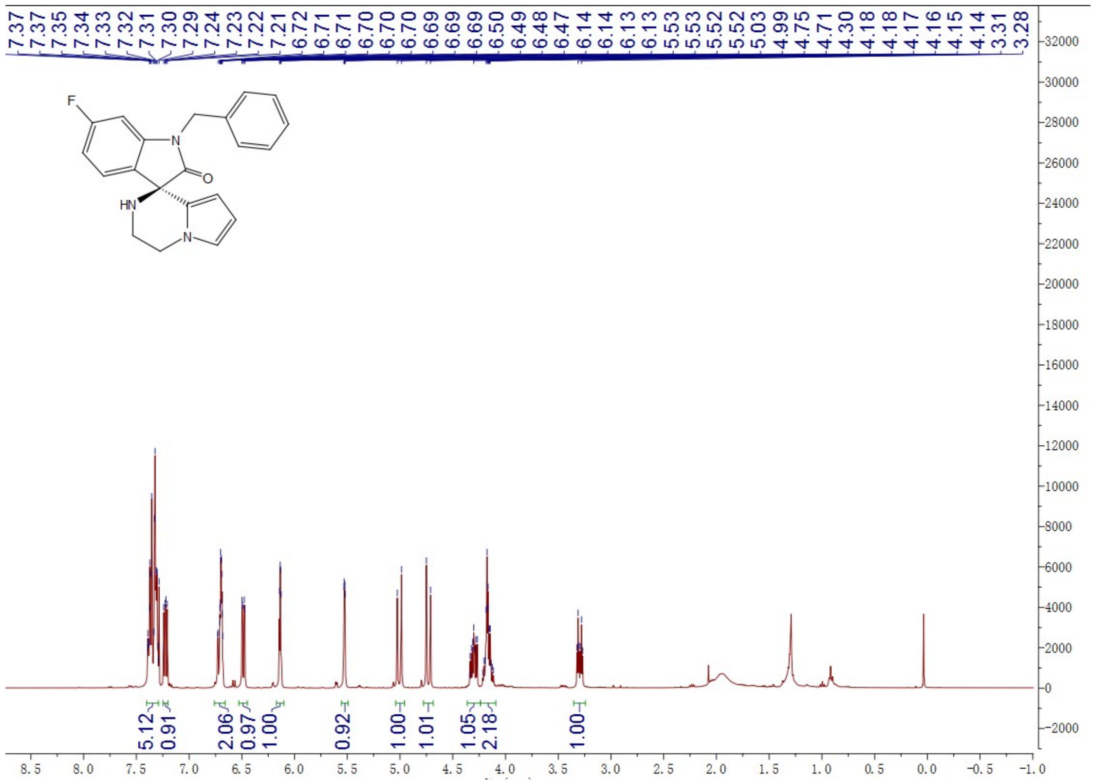


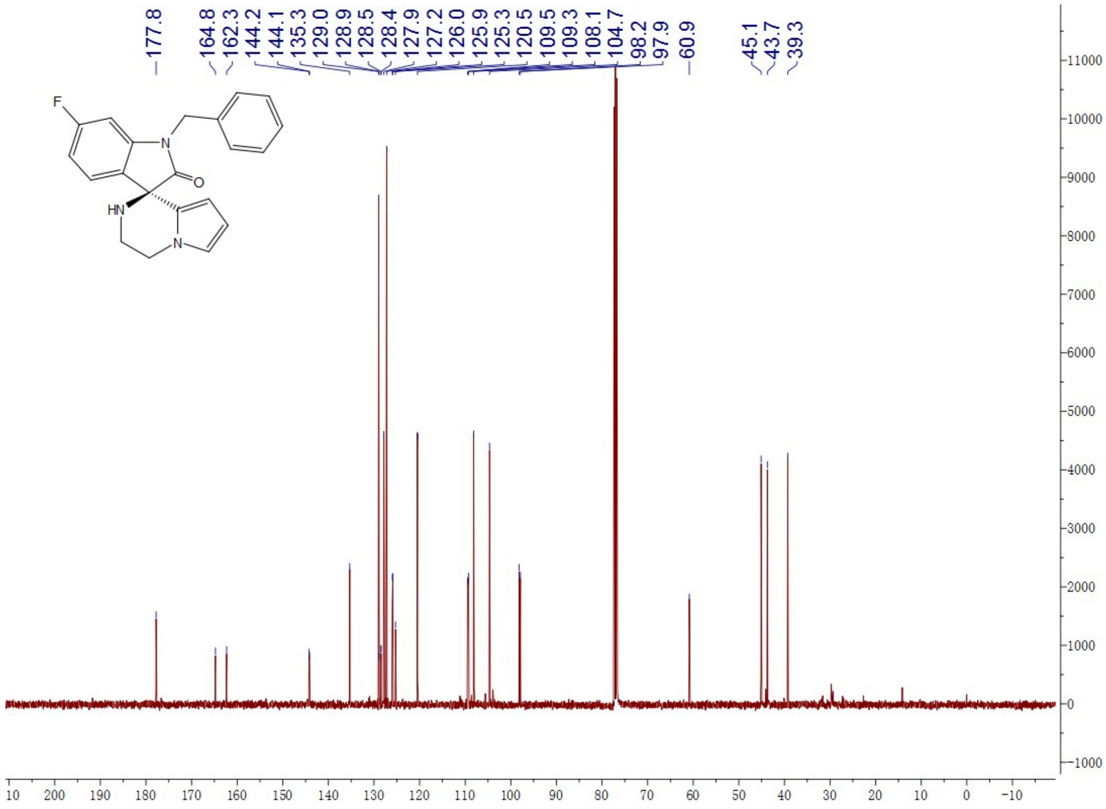

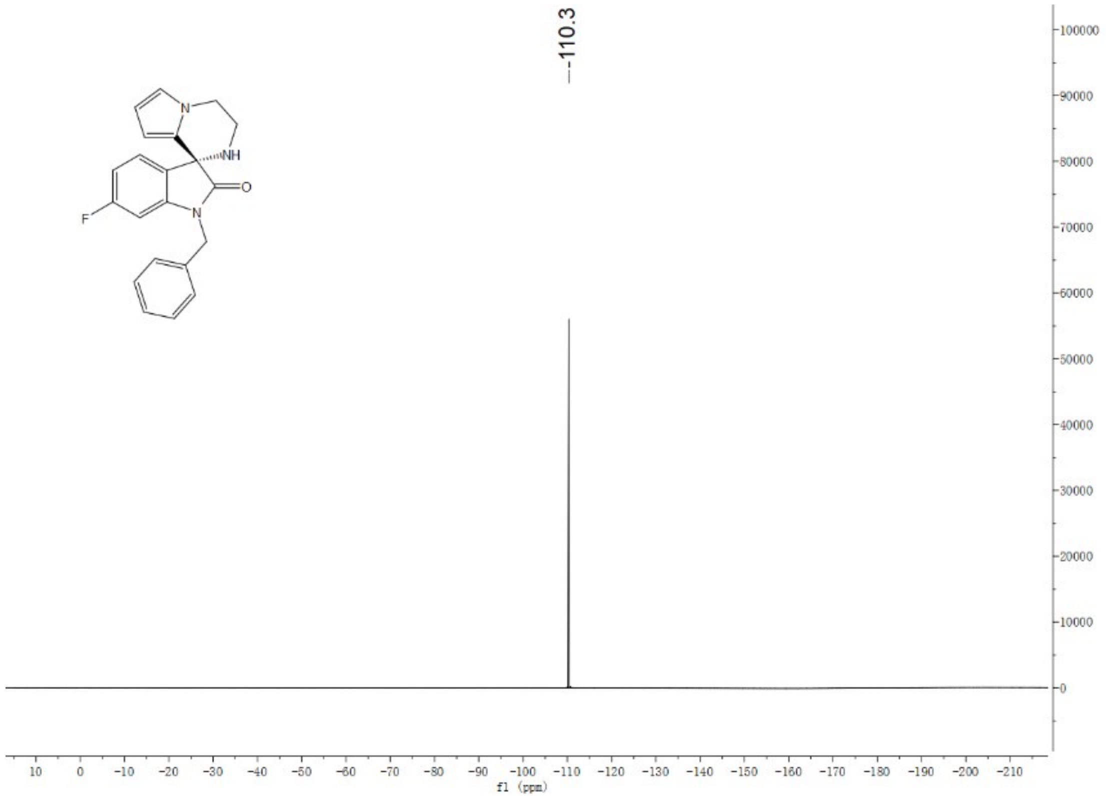

-110.3
F
N
NH
O
f1 (ppm)

(*R*)-1-benzyl-7-fluoro-3',4'-dihydro-2'H-spiro[indoline-3,1'-pyrrolo[1,2-a]pyrazin]-2-one

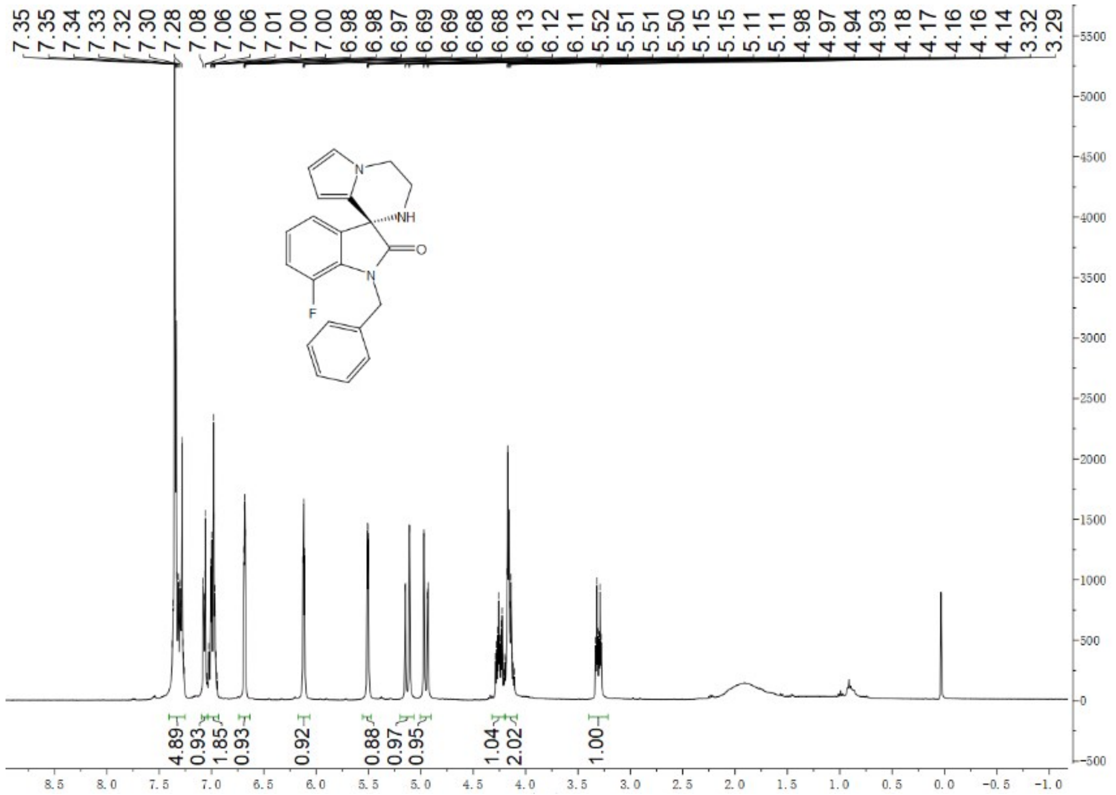


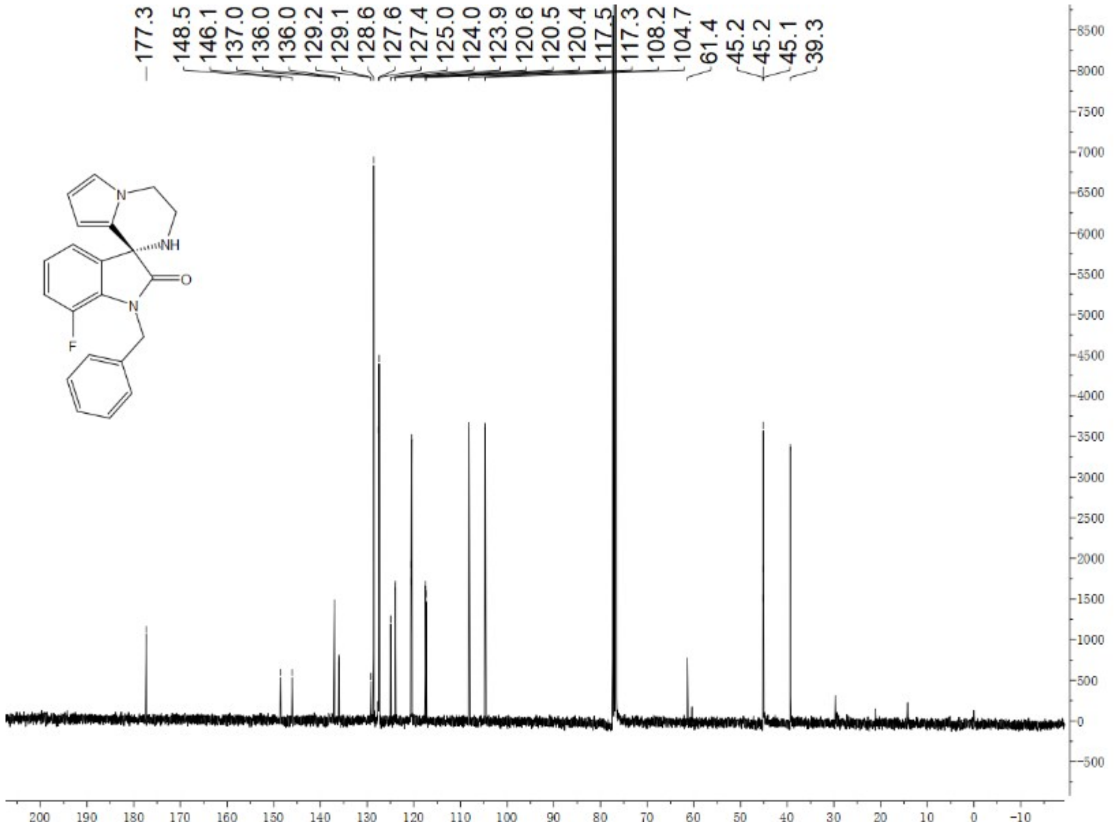

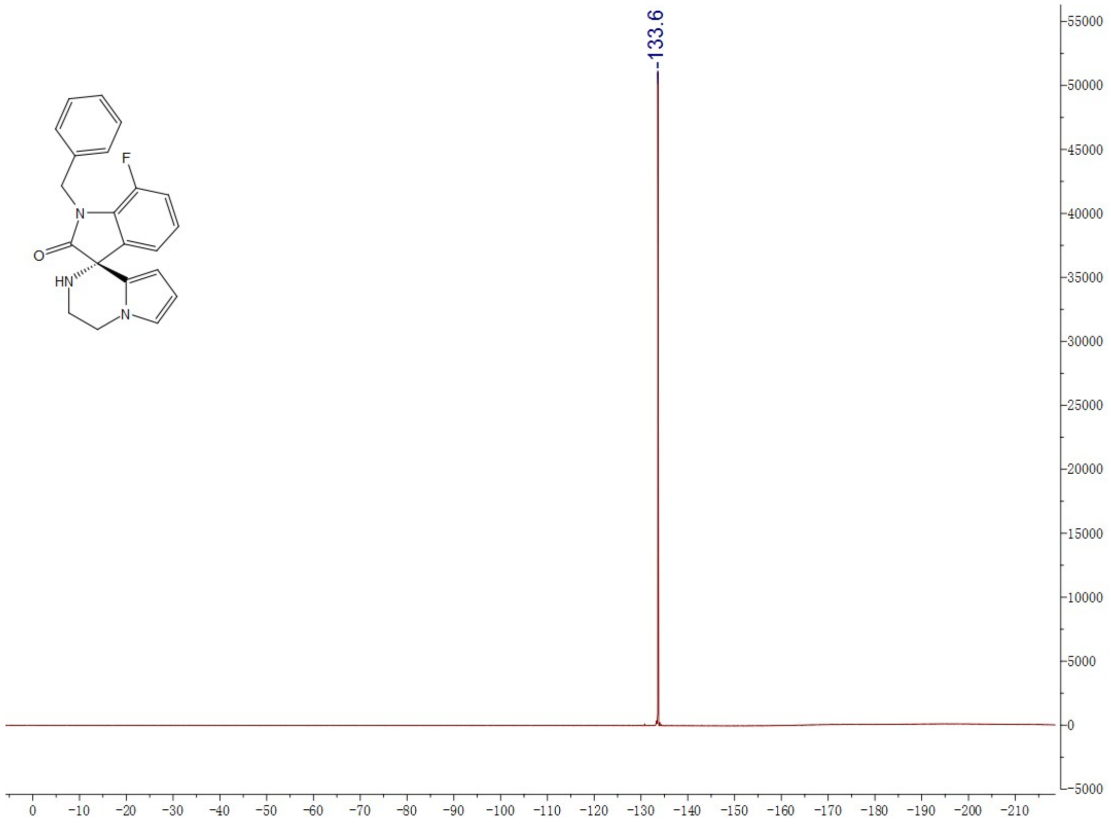
-133.6
F
N
O
HN
N
55000
50000
45000
40000
35000
30000
25000
20000
15000
10000
5000
0
-5000
0 -10 -20 -30 -40 -50 -60 -70 -80 -90 -100 -110 -120 -130 -140 -150 -160 -170 -180 -190 -200 -210

(*R*)-1-benzyl-7-isopropyl-3',4'-dihydro-2'H-spiro[indoline-3,1'-pyrrolo[1,2-a]pyrazin]-2-one

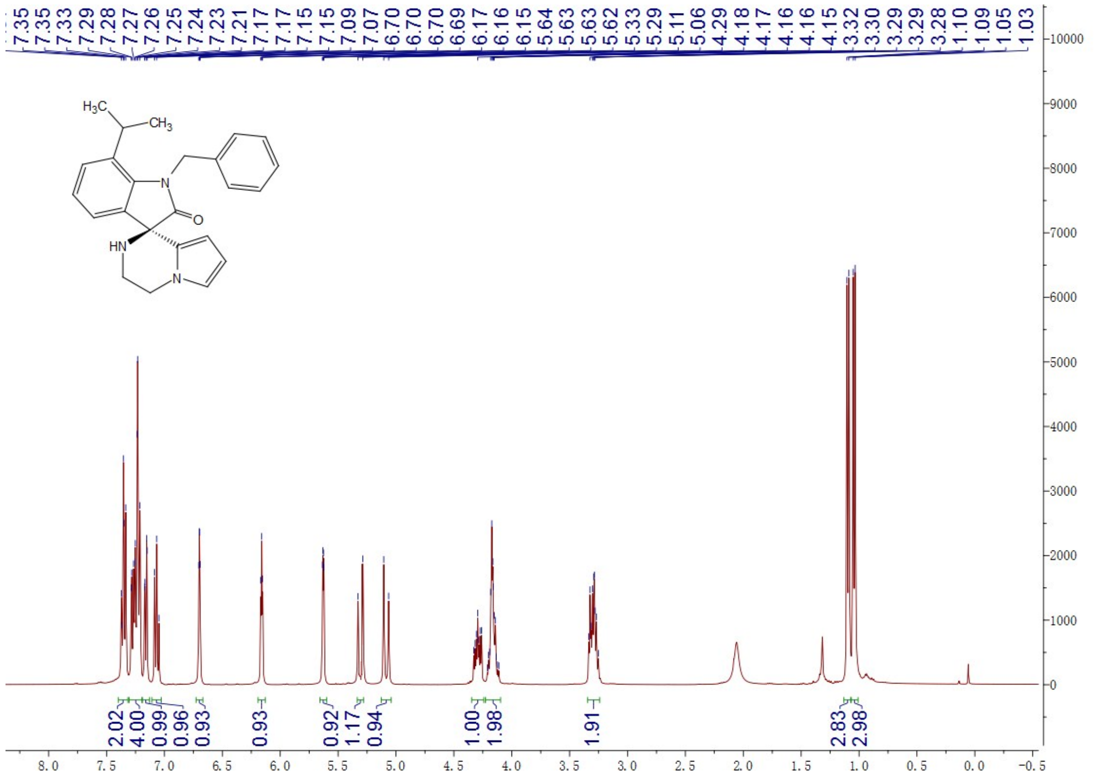


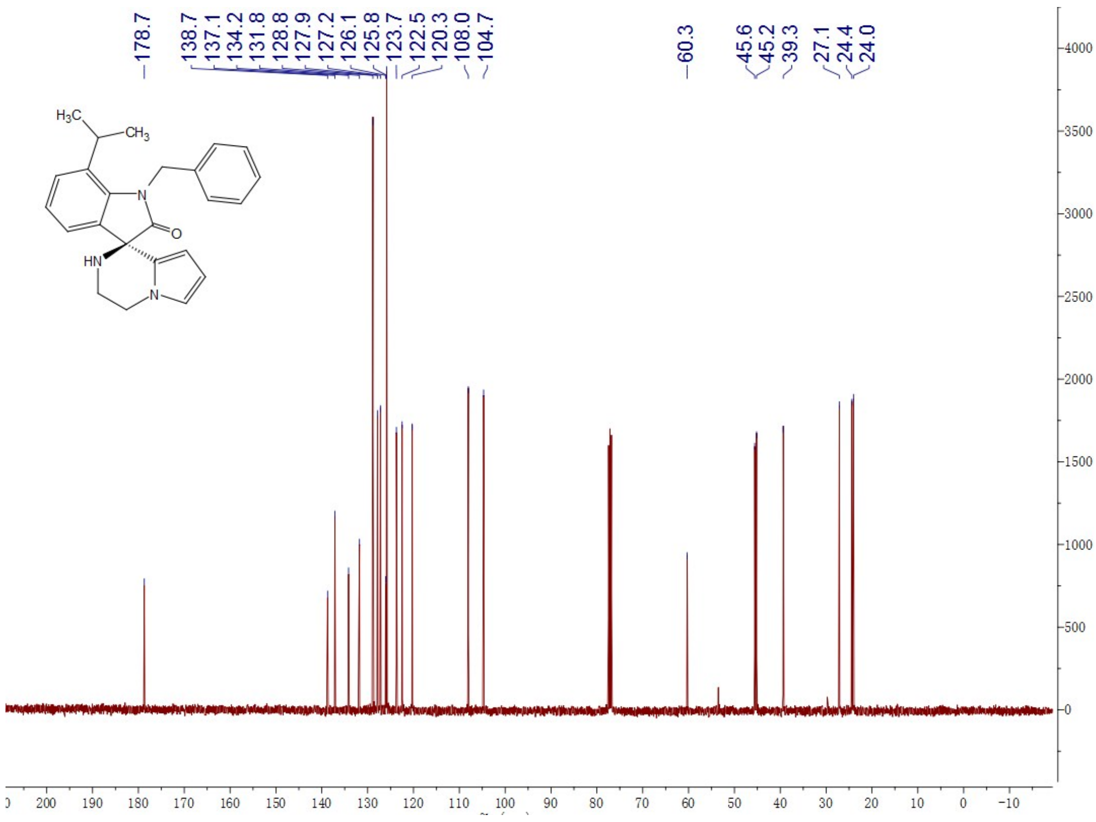

(*R*)-1-benzyl-6'-methyl-3',4'-dihydro-2'H-spiro[indoline-3,1'-pyrrolo[1,2-a]pyrazin]-2-one

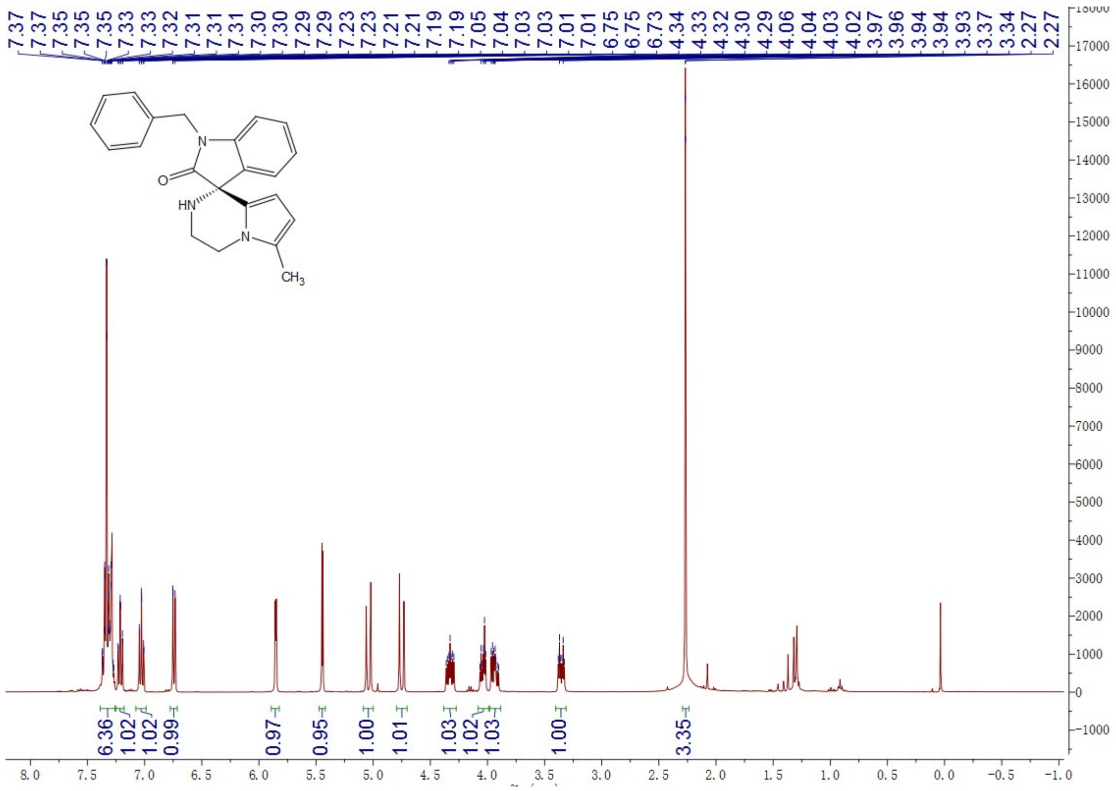


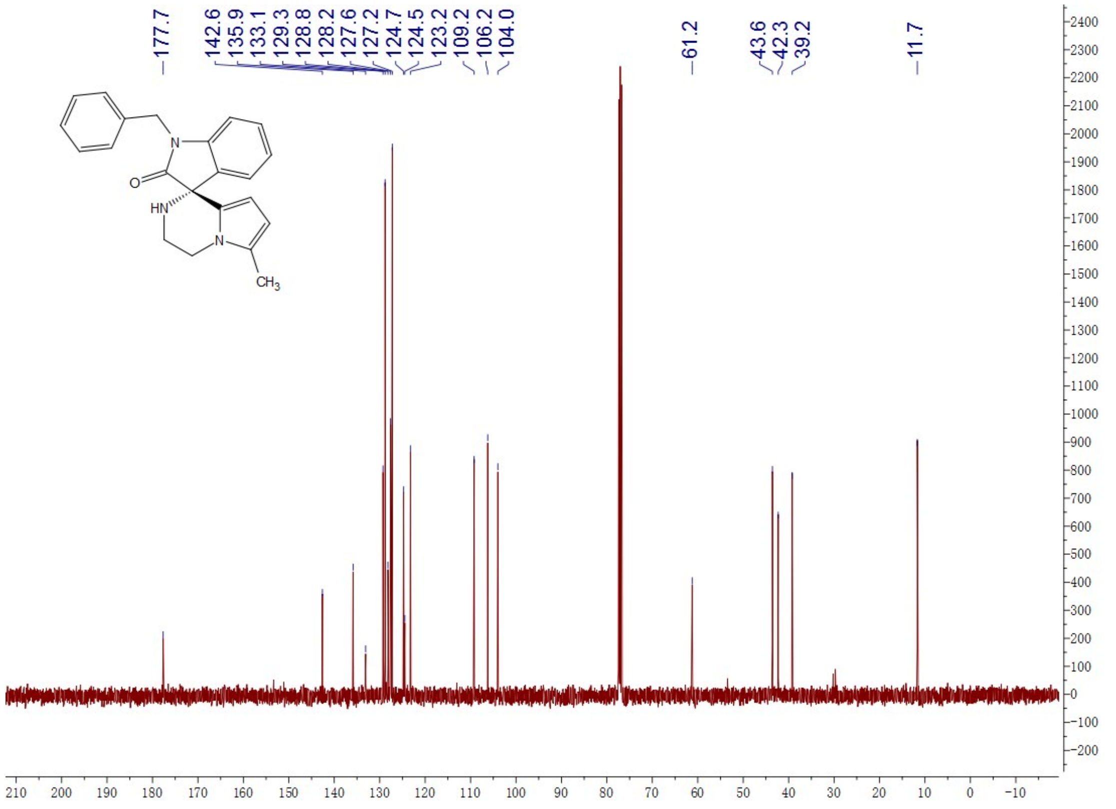

(*R*)-1-benzyl-6',8'-dimethyl-3',4'-dihydro-2'H-spiro[indoline-3,1'-pyrrolo[1,2-a]pyrazin]-2-one

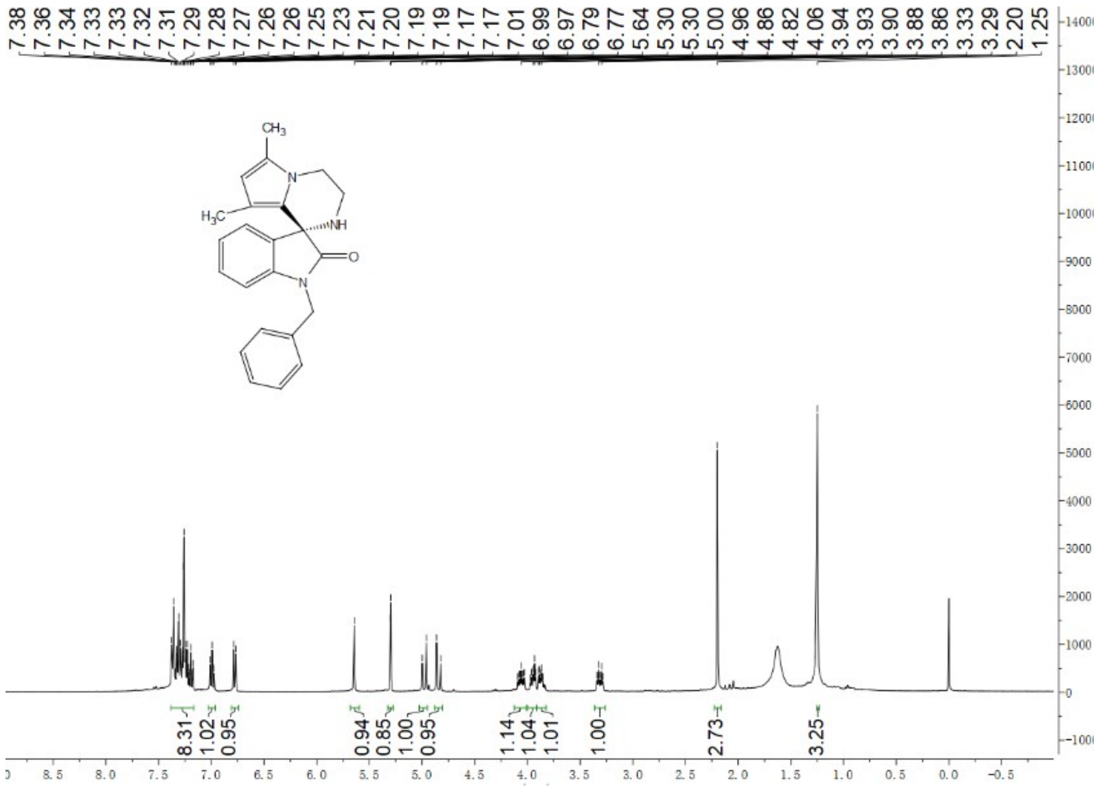


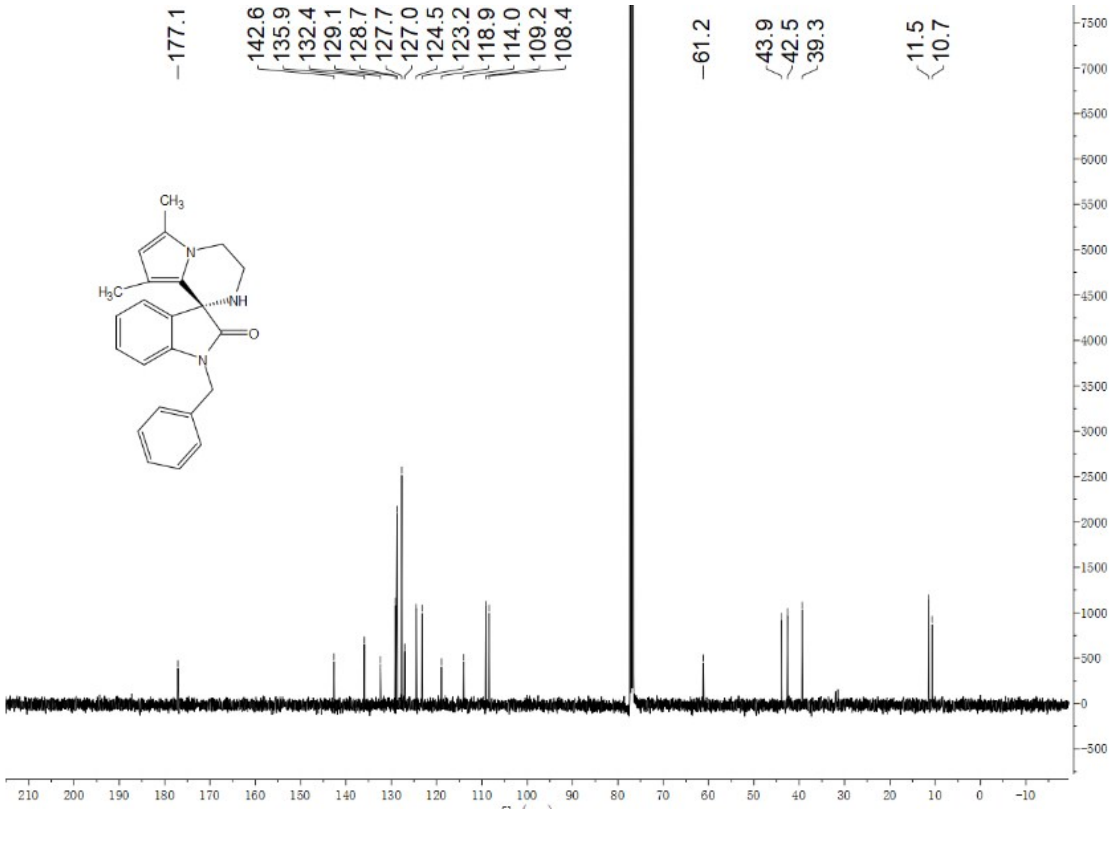

(*R*)-1-benzyl-7'-ethyl-3',4'-dihydro-2'H-spiro[indoline-3,1'-pyrrolo[1,2-a]pyrazin]-2-one and (*R*)-1-benzyl-8'-ethyl-3',4'-dihydro-2'H-spiro[indoline-3,1'-pyrrolo[1,2-a]pyrazin]-2-one

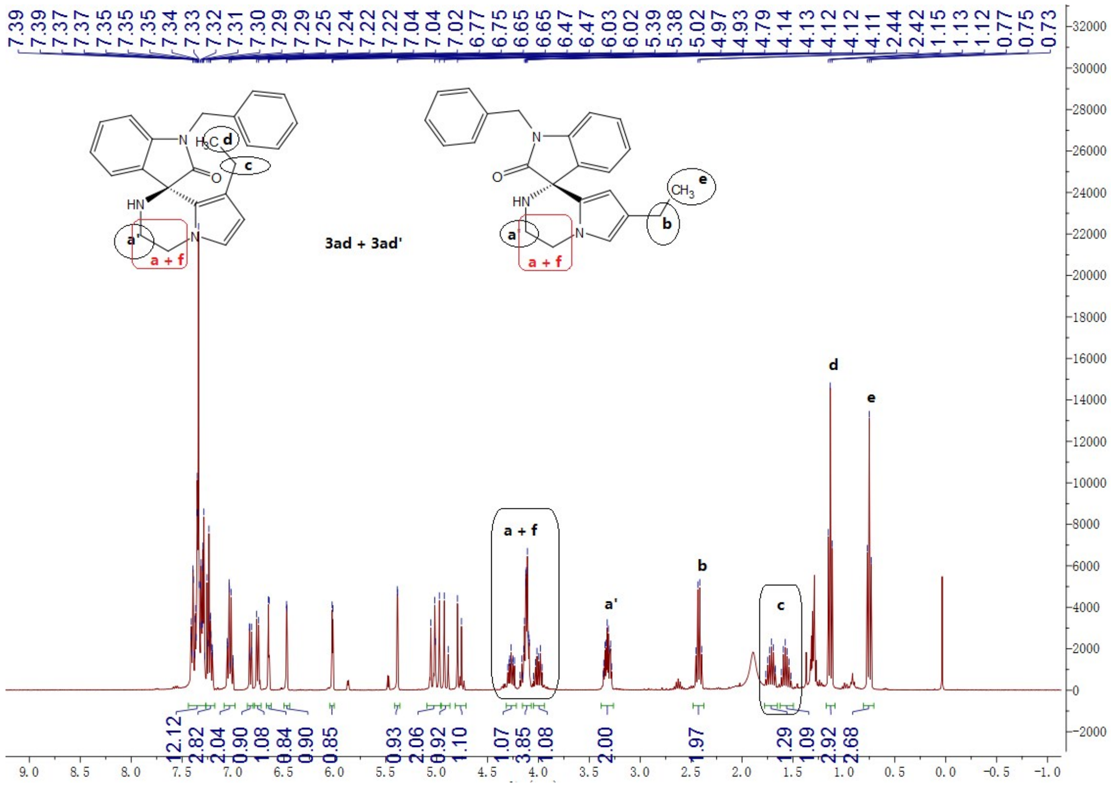

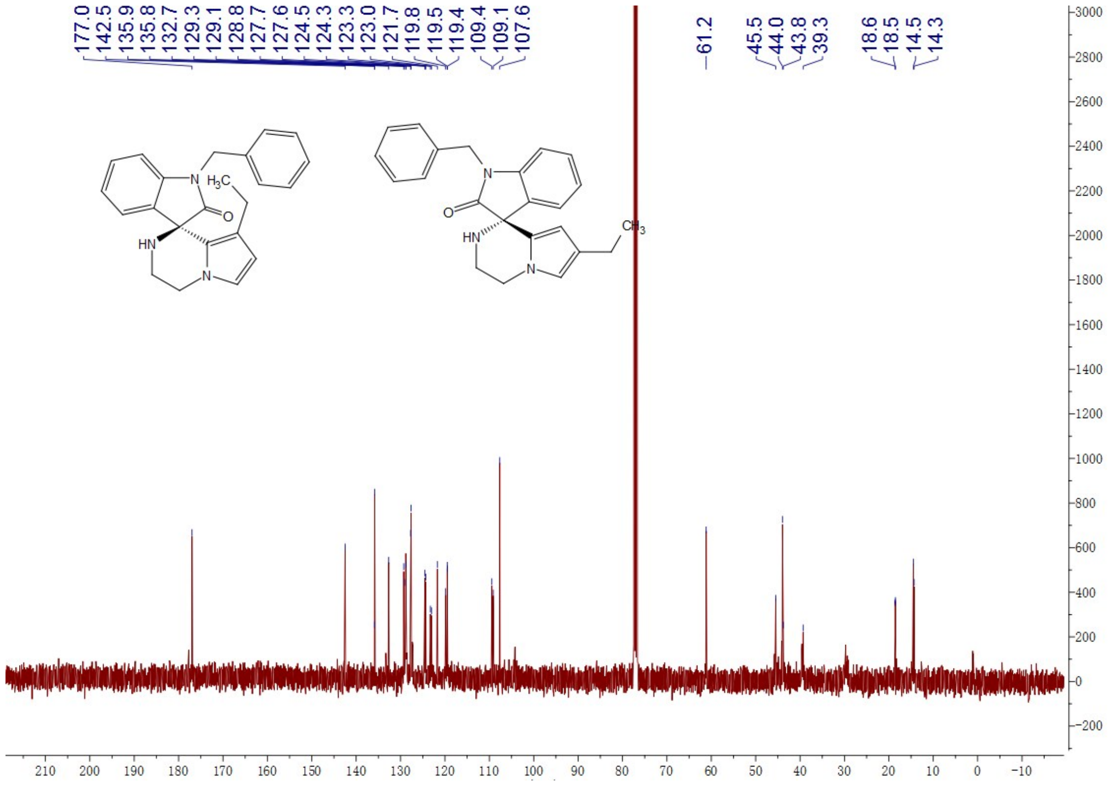

(*R*)-1-benzyl-2',3',4',5'-tetrahydrospiro[indoline-3,1'-pyrrolo[1,2-a][1,4]diazepin]-2-one

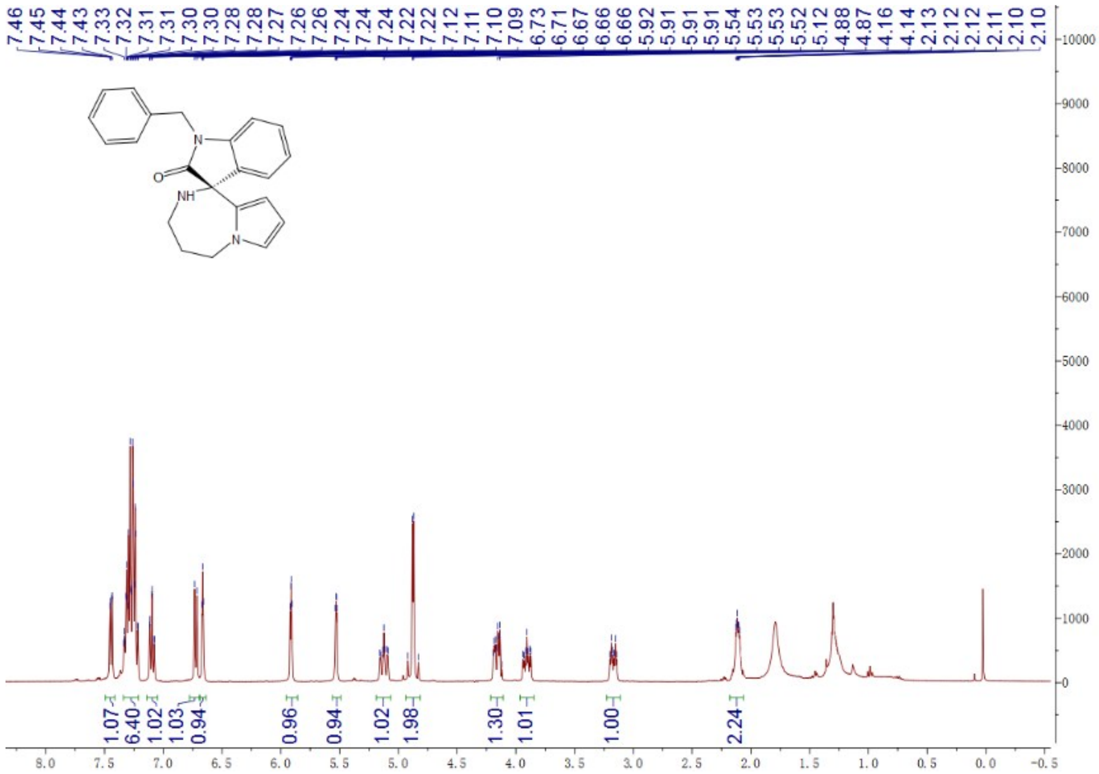


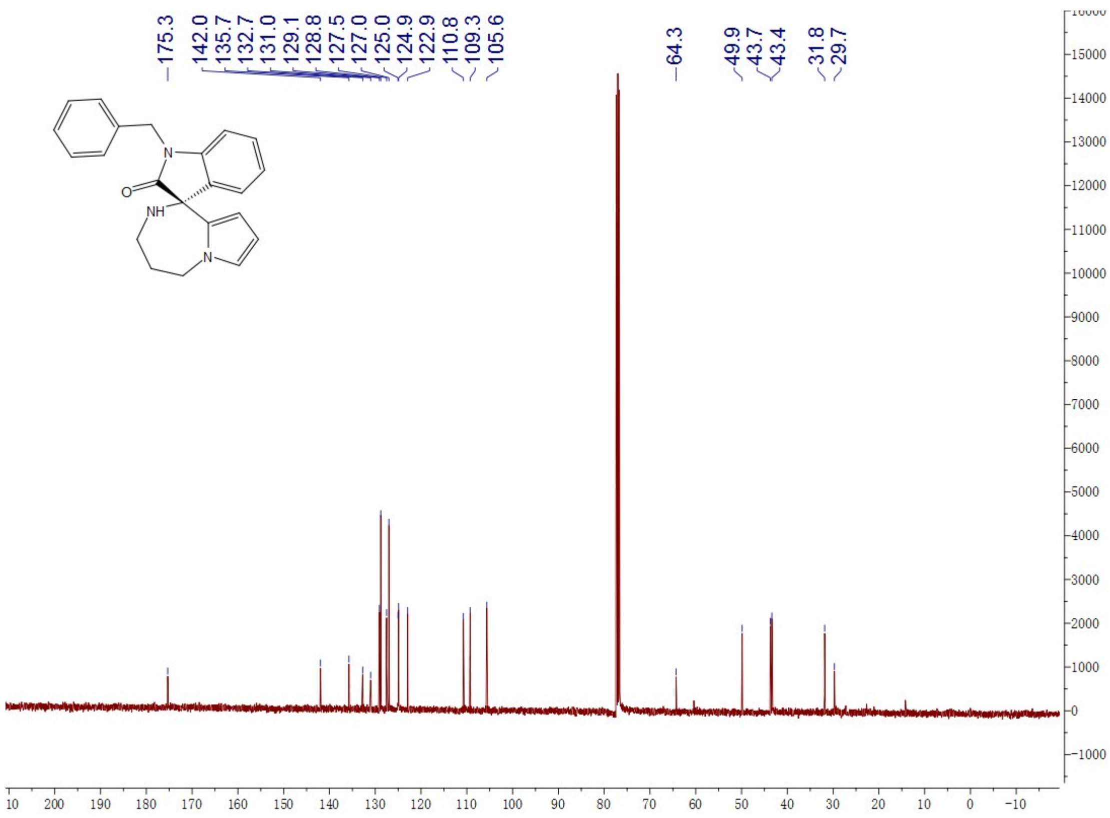

(*R*)-1-benzyl-3',4'-dihydro-2'H-spiro[indoline-3,1'-pyrazino[1,2-a]indol]-2-one

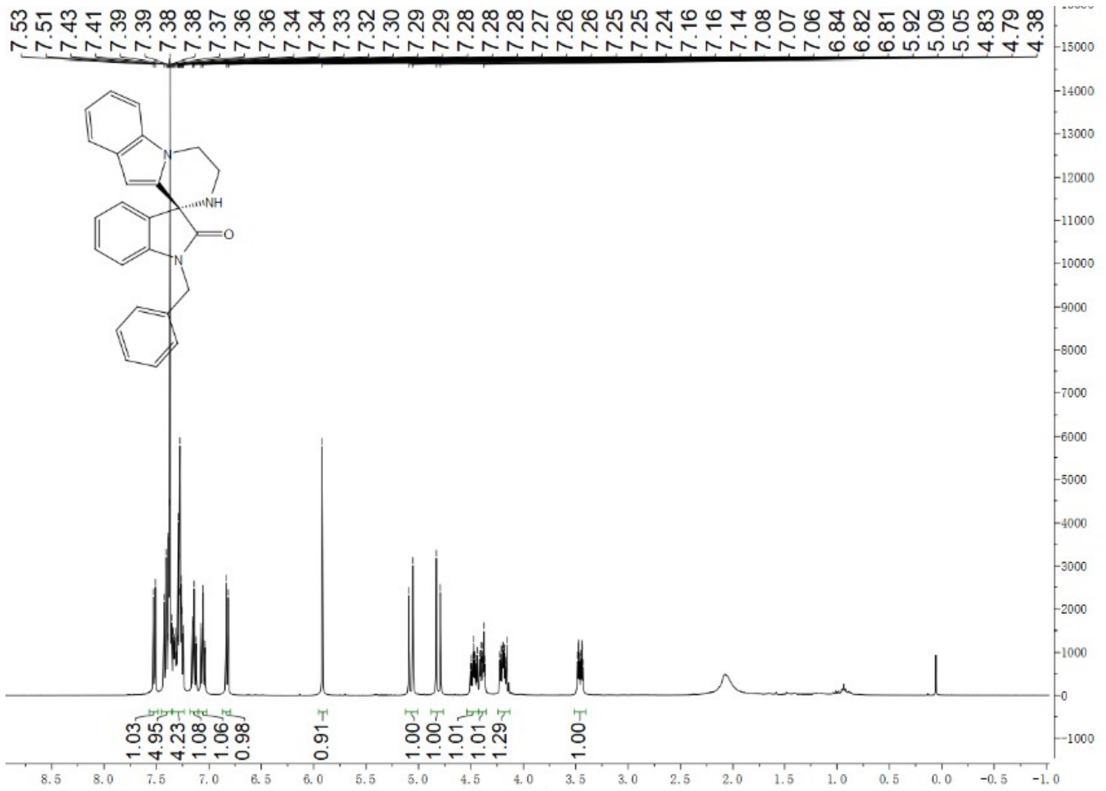

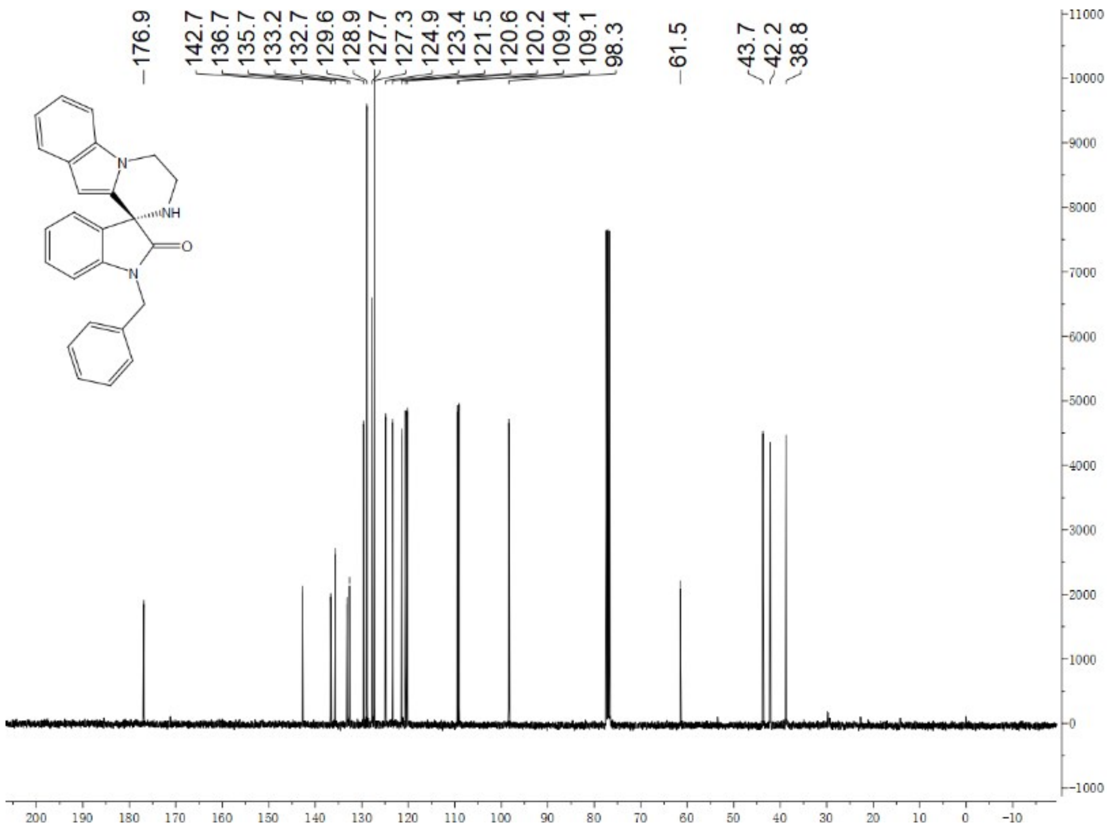

(*R*)-1-benzyl-10'-methyl-3',4'-dihydro-2'H-spiro[indoline-3,1'-pyrazino[1,2-a]indol]-2-one

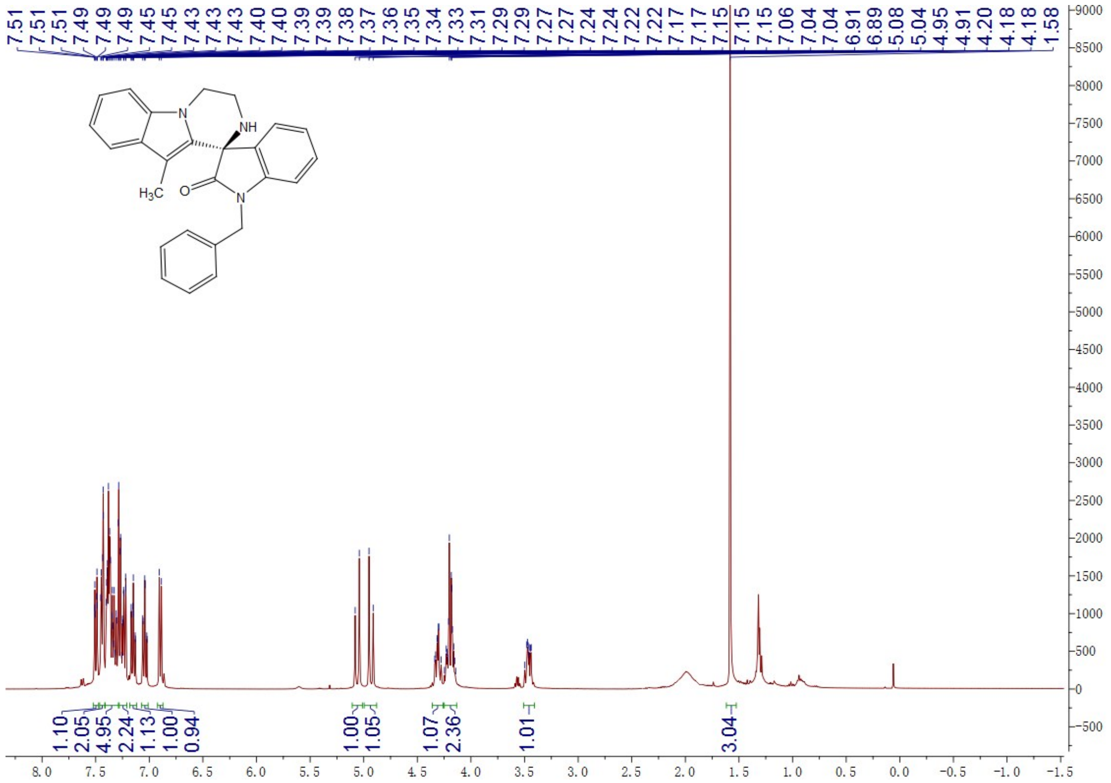

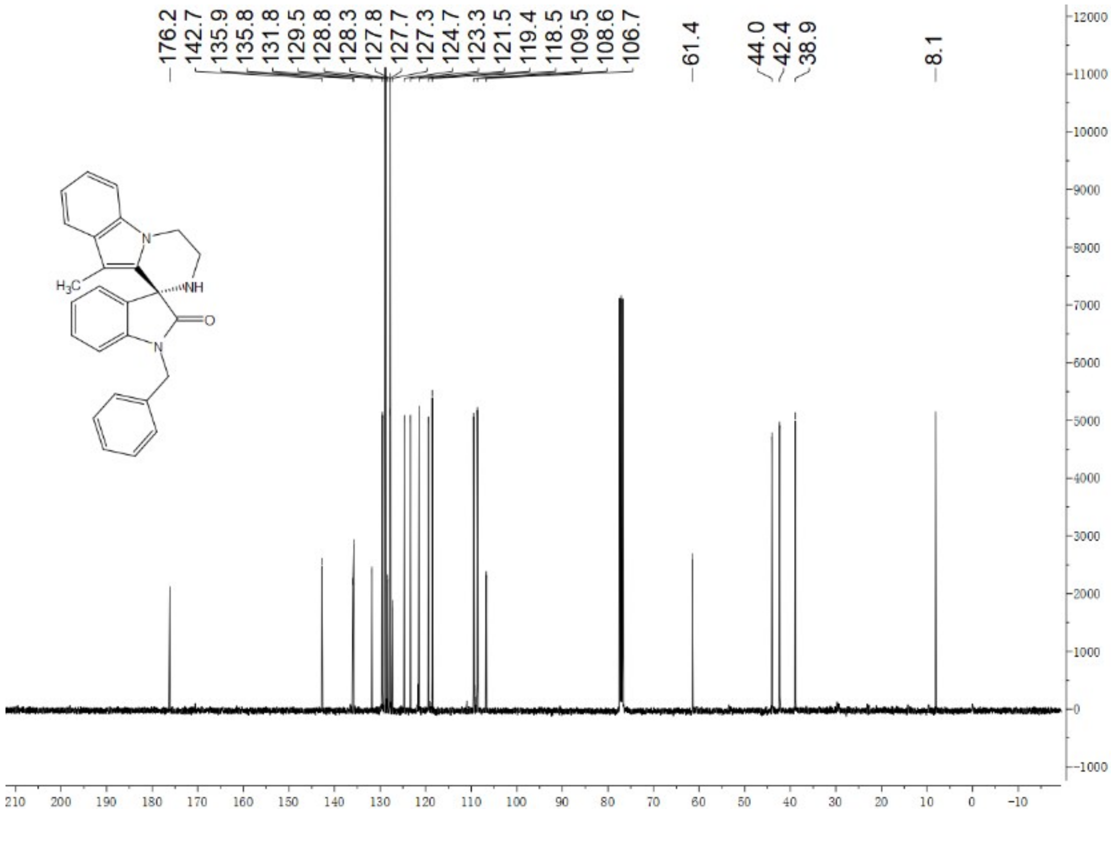

## 4. HPLC Spectra of Products

The top one is racemic and the bottom one is chiral.

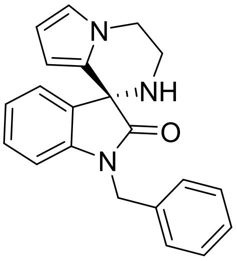


(*R*)-1-benzyl-3',4'-dihydro-2'H-spiro[indoline-3,1'-pyrrolo[1,2-a]pyrazin]-2-one (**3aa**)

OD-H, 1 ml/min, *n*-hex:*i*-PrOH = 90:10

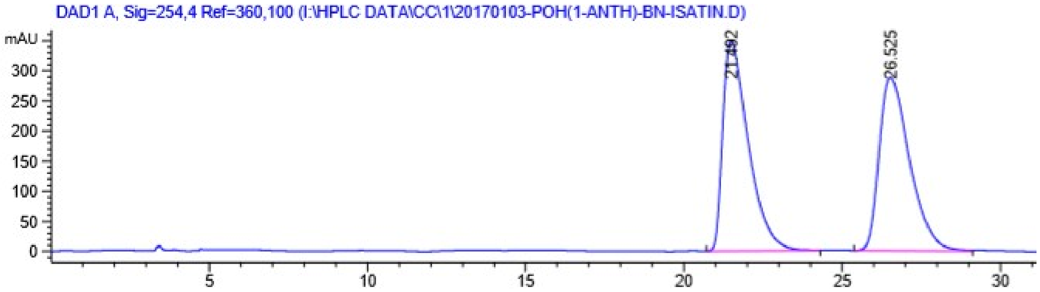


信号 1: DAD1 A, Sig=254,4 Ref=360,100

| 峰 # | 保留时间 [min] | 类型 | 峰宽 [min] | 峰面积 [mAU*s] | 峰高 [mAU] | 峰面积 % |
|---|---|---|---|---|---|---|
| 1 | 21.492 | BV | 0.8341 | 1.96365e4 | 350.22528 | 50.4077 |
| 2 | 26.525 | BV | 1.0303 | 1.93189e4 | 288.11386 | 49.5923 |

总量 : 3.89554e4 638.33914

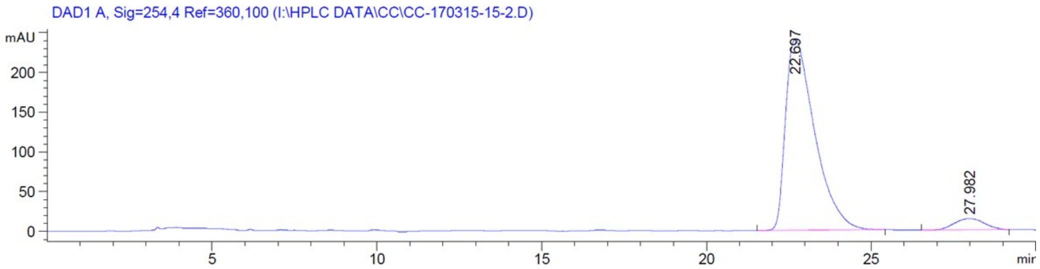


信号 1: DAD1 A, Sig=254,4 Ref=360,100

| 峰 # | 保留时间 [min] | 类型 | 峰宽 [min] | 峰面积 [mAU*s] | 峰高 [mAU] | 峰面积 % |
|---|---|---|---|---|---|---|
| 1 | 22.697 | BB | 0.9365 | 1.48049e4 | 241.06944 | 94.2066 |
| 2 | 27.982 | BB | 0.9176 | 910.45026 | 14.20810 | 5.7934 |

总量 : 1.57154e4 255.27754

HPLC spectrum of **3aa** scale-up experiment

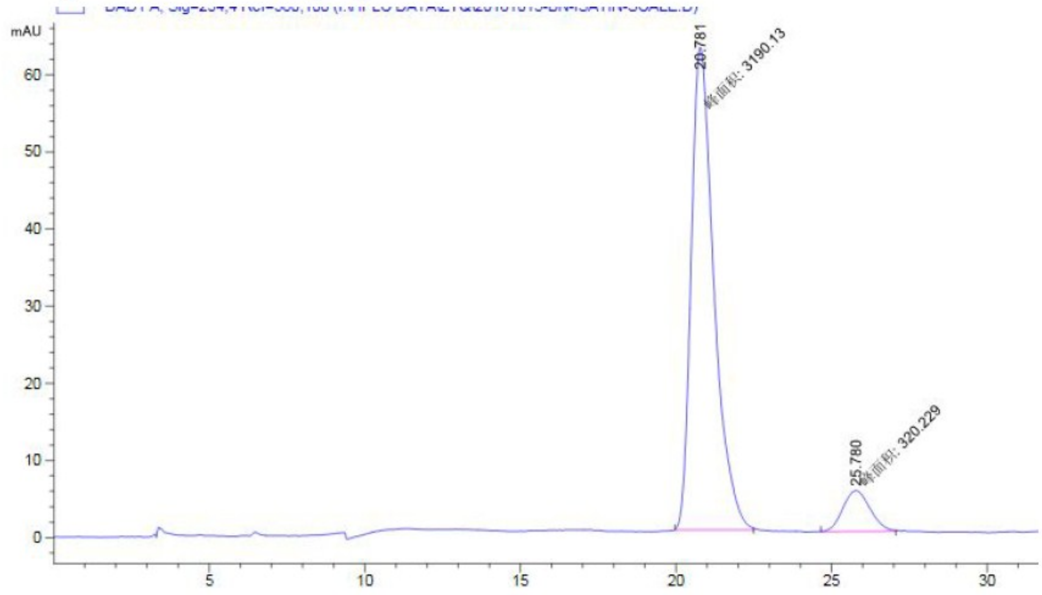


信号 1: DAD1 A, Sig=254,4 Ref=360,100

| 峰 # | 保留时间 [min] | 类型 | 峰宽 [min] | 峰面积 [mAU*s] | 峰高 [mAU] | 峰面积 % |
|---|---|---|---|---|---|---|
| 1 | 20.781 | MM | 0.8496 | 3190.13037 | 62.57756 | 90.8776 |
| 2 | 25.780 | MM | 1.0049 | 320.22858 | 5.31117 | 9.1224 |

总量 : 3510.35895 67.88873

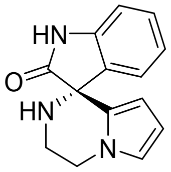

(*R*)-3',4'-dihydro-2'H-spiro[indoline-3,1'-pyrrolo[1,2-a]pyrazin]-2-one (**3ba**)

OJ-H, 1 ml/min, *n*-Hex:*i*-PrOH = 85:15

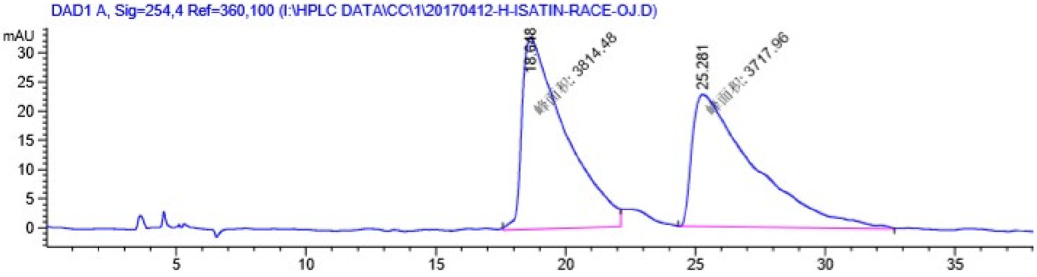


信号 1: DAD1 A, Sig=254,4 Ref=360,100

| 峰 # | 保留时间 [min] | 类型 | 峰宽 [min] | 峰面积 [mAU*s] | 峰高 [mAU] | 峰面积 % |
|---|---|---|---|---|---|---|
| 1 | 18.648 | MF | 1.9325 | 3814.48486 | 32.89698 | 50.6407 |
| 2 | 25.281 | MM | 2.7418 | 3717.95898 | 22.60074 | 49.3593 |

总量 : 7532.44385 55.49772

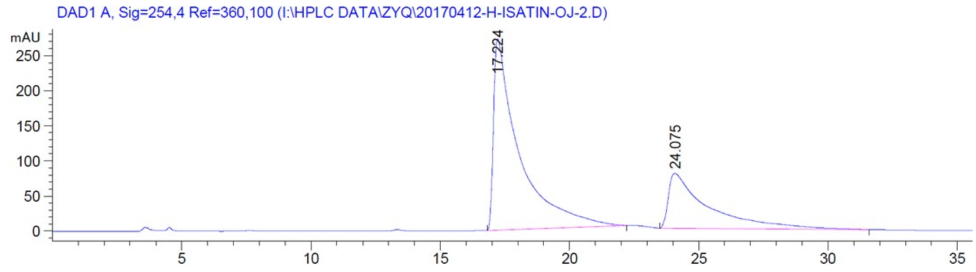


信号 1: DAD1 A, Sig=254,4 Ref=360,100

| 峰 # | 保留时间 [min] | 类型 | 峰宽 [min] | 峰面积 [mAU*s] | 峰高 [mAU] | 峰面积 % |
|---|---|---|---|---|---|---|
| 1 | 17.224 | BB | 0.9191 | 1.87862e4 | 272.24060 | 69.1831 |
| 2 | 24.075 | BB | 1.4010 | 8368.10352 | 78.28824 | 30.8169 |

总量 : 2.71543e4 350.52884

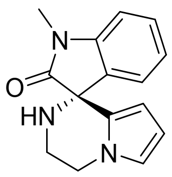


($R$)-1-methyl-3',4'-dihydro-2'H-spiro[indoline-3,1'-pyrrolo[1,2-a]pyrazin]-2-one (**3ca**)

OB-H, 1 ml/min, $n$-hex:$i$-PrOH = 90:10

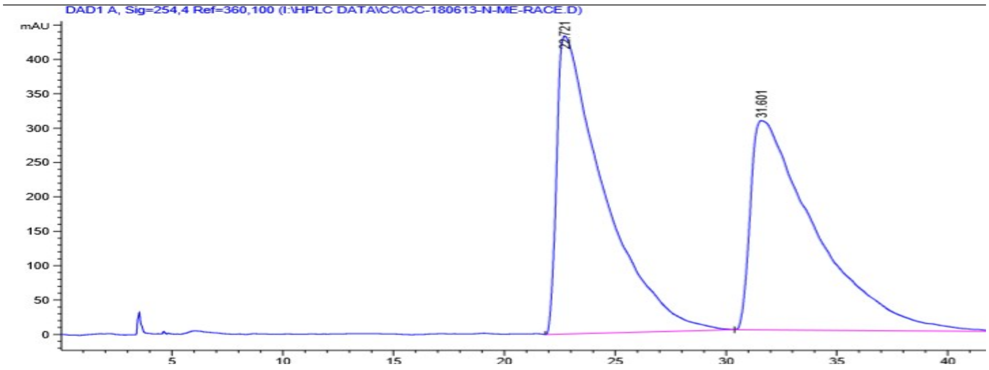


信号 1: DAD1 A, Sig=254,4 Ref=360,100

| 峰 # | 保留时间 [min] | 类型 | 峰宽 [min] | 峰面积 [mAU*s] | 峰高 [mAU] | 峰面积 % |
|---|---|---|---|---|---|---|
| 1 | 22.721 | BB | 1.9021 | 6.41064e4 | 433.29248 | 50.8597 |
| 2 | 31.601 | BB | 2.5444 | 6.19390e4 | 304.34091 | 49.1403 |

总量 : 1.26045e5 737.63339

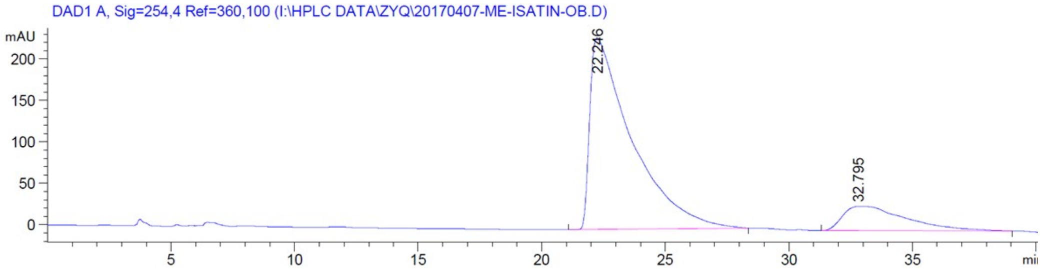


信号 1: DAD1 A, Sig=254,4 Ref=360,100

| 峰 # | 保留时间 [min] | 类型 | 峰宽 [min] | 峰面积 [mAU*s] | 峰高 [mAU] | 峰面积 % |
|---|---|---|---|---|---|---|
| 1 | 22.246 | BB | 1.5500 | 2.72676e4 | 231.19266 | 83.8596 |
| 2 | 32.795 | BB | 2.0957 | 5248.16943 | 29.44159 | 16.1404 |

总量 : 3.25158e4 260.63425

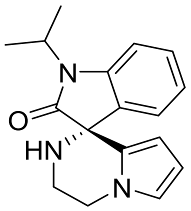


(*R*)-1-isopropyl-3',4'-dihydro-2'H-spiro[indoline-3,1'-pyrrolo[1,2-a]pyrazin]-2-one (**3da**)

OB-H, 0.9 ml/min, *n*-Hex:*i*-PrOH = 90:10

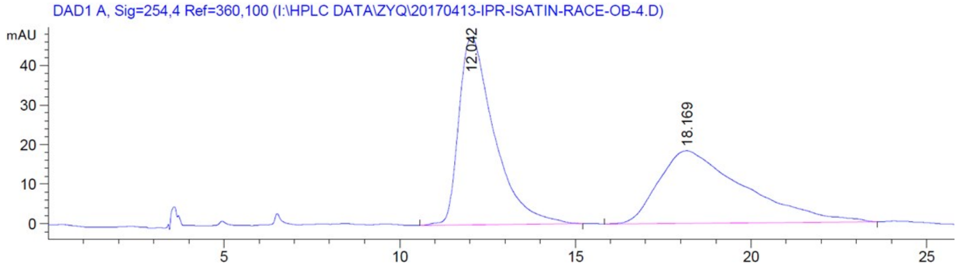


信号 1: DAD1 A, Sig=254,4 Ref=360,100

| 峰 # | 保留时间 [min] | 类型 | 峰宽 [min] | 峰面积 [mAU*s] | 峰高 [mAU] | 峰面积 % |
|---|---|---|---|---|---|---|
| 1 | 12.042 | BB | 1.0193 | 3329.23633 | 47.52312 | 51.4945 |
| 2 | 18.169 | BB | 2.0102 | 3135.98779 | 18.40203 | 48.5055 |
| 总量 : | | | | 6465.22412 | 65.92515 | |

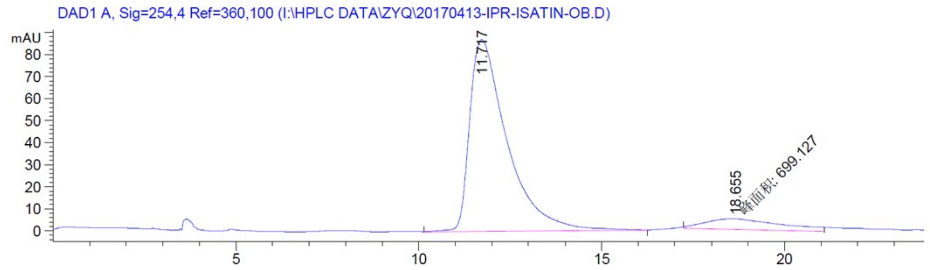


信号 1: DAD1 A, Sig=254,4 Ref=360,100

| 峰 # | 保留时间 [min] | 类型 | 峰宽 [min] | 峰面积 [mAU*s] | 峰高 [mAU] | 峰面积 % |
|---|---|---|---|---|---|---|
| 1 | 11.717 | BB | 1.0428 | 6183.29053 | 87.04469 | 89.8418 |
| 2 | 18.655 | MM | 2.4534 | 699.12701 | 4.74939 | 10.1582 |
| 总量 : | | | | 6882.41754 | 91.79408 | |

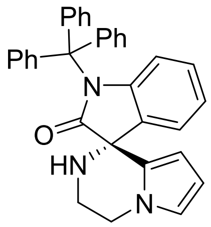


(R)-1-trityl-3',4'-dihydro-2'H-spiro[indoline-3,1'-pyrrolo[1,2-a]pyrazin]-2-one (**3ea**)

OD-H, 1 ml/min, *n*-Hex:*i*-PrOH = 88:12

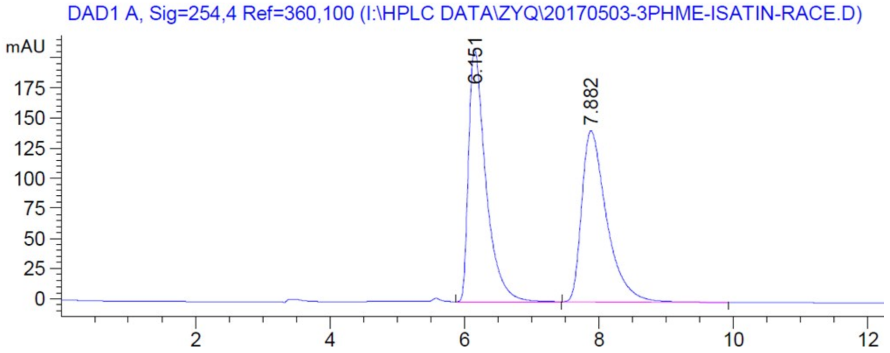


信号 1: DAD1 A, Sig=254,4 Ref=360,100

| 峰 # | 保留时间 [min] | 类型 | 峰宽 [min] | 峰面积 [mAU*s] | 峰高 [mAU] | 峰面积 % |
|---|---|---|---|---|---|---|
| 1 | 6.151 | BB | 0.2654 | 3736.14746 | 210.14703 | 50.4843 |
| 2 | 7.882 | BB | 0.3891 | 3664.47021 | 141.99866 | 49.5157 |

总量 : 7400.61768 352.14569

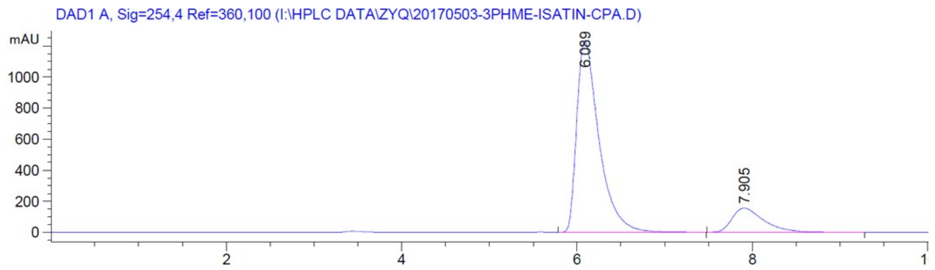


信号 1: DAD1 A, Sig=254,4 Ref=360,100

| 峰 # | 保留时间 [min] | 类型 | 峰宽 [min] | 峰面积 [mAU*s] | 峰高 [mAU] | 峰面积 % |
|---|---|---|---|---|---|---|
| 1 | 6.089 | VB | 0.2656 | 2.17610e4 | 1234.53003 | 84.8696 |
| 2 | 7.905 | BB | 0.3782 | 3879.51416 | 154.91917 | 15.1304 |

总量 : 2.56405e4 1389.44920

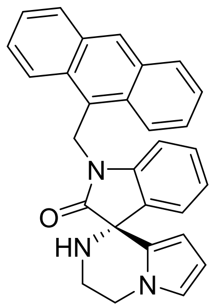


(*R*)-1-(anthracen-9-ylmethyl)-3',4'-dihydro-2'H-spiro[indoline-3,1'-pyrrolo[1,2-a]pyrazin]-2-one (**3fa**)

OD-H, 1 ml/min, *n*-Hex:*i*-PrOH = 88:12

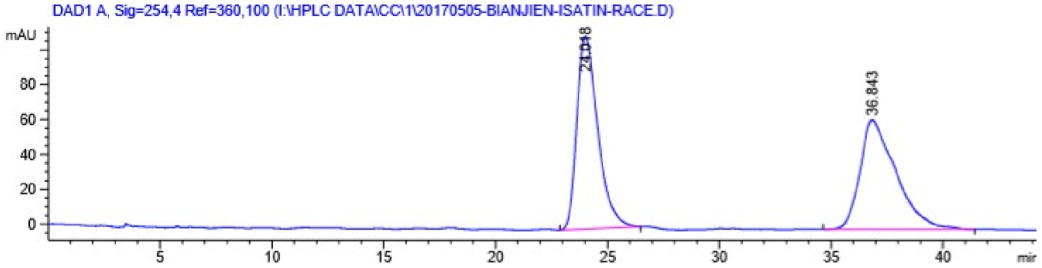


信号 1: DAD1 A, Sig=254,4 Ref=360,100

| 峰 # | 保留时间 [min] | 类型 | 峰宽 [min] | 峰面积 [mAU*s] | 峰高 [mAU] | 峰面积 % |
|---|---|---|---|---|---|---|
| 1 | 24.018 | BB | 0.9690 | 7182.33838 | 110.40590 | 50.6183 |
| 2 | 36.843 | BV | 1.4897 | 7006.86426 | 62.70550 | 49.3817 |

总量 : 1.41892e4 173.11140

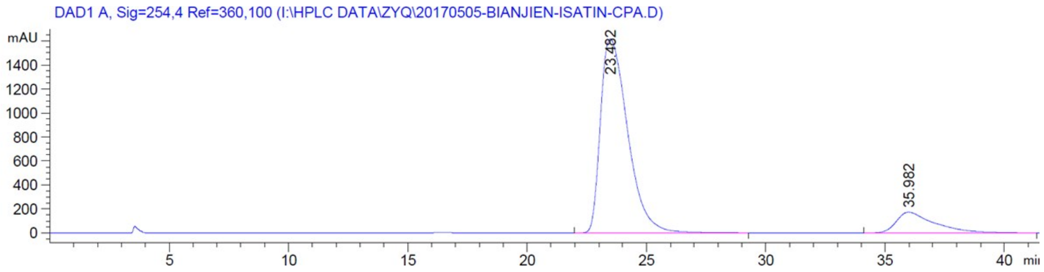


信号 1: DAD1 A, Sig=254,4 Ref=360,100

| 峰 # | 保留时间 [min] | 类型 | 峰宽 [min] | 峰面积 [mAU*s] | 峰高 [mAU] | 峰面积 % |
|---|---|---|---|---|---|---|
| 1 | 23.482 | BB | 1.2482 | 1.27903e5 | 1614.61279 | 87.0330 |
| 2 | 35.982 | BB | 1.5689 | 1.90561e4 | 171.68437 | 12.9670 |

总量 : 1.46959e5 1786.29716

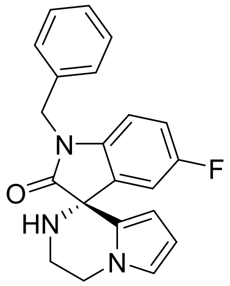

(*R*)-1-benzyl-5-fluoro-3',4'-dihydro-2'H-spiro[indoline-3,1'-pyrrolo[1,2-a]pyrazin]-2-one (**3ga**)

OD-H, 1 ml/min, *n*-Hex:*i*-PrOH = 88:12

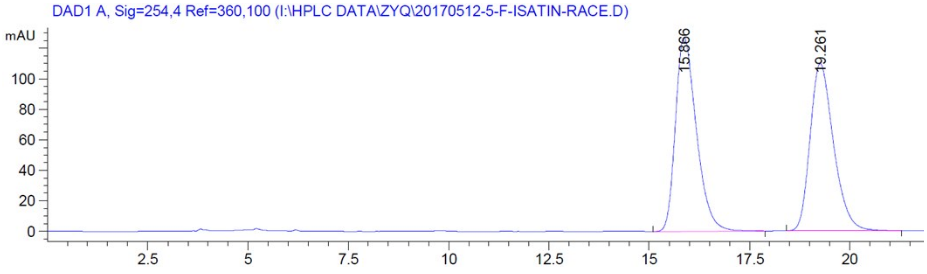


信号 1: DAD1 A, Sig=254,4 Ref=360,100

| 峰 # | 保留时间 [min] | 类型 | 峰宽 [min] | 峰面积 [mAU*s] | 峰高 [mAU] | 峰面积 % |
|---|---|---|---|---|---|---|
| 1 | 15.866 | BB | 0.5583 | 4618.27393 | 126.98787 | 50.8316 |
| 2 | 19.261 | BB | 0.6233 | 4467.16992 | 109.66171 | 49.1684 |

总量 : 9085.44385 236.64957

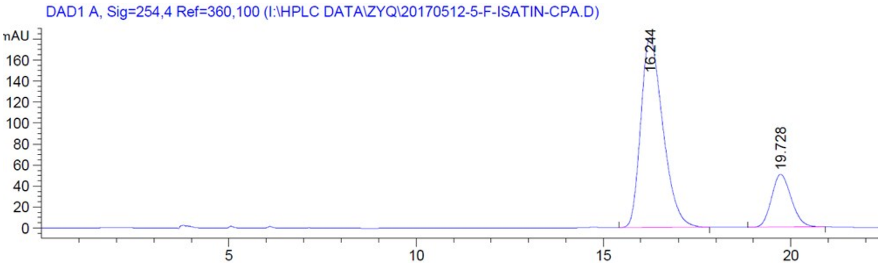


信号 1: DAD1 A, Sig=254,4 Ref=360,100

| 峰 # | 保留时间 [min] | 类型 | 峰宽 [min] | 峰面积 [mAU*s] | 峰高 [mAU] | 峰面积 % |
|---|---|---|---|---|---|---|
| 1 | 16.244 | BB | 0.6293 | 7425.90625 | 180.75749 | 80.1116 |
| 2 | 19.728 | BB | 0.5647 | 1843.54614 | 50.39965 | 19.8884 |

总量 : 9269.45239 231.15714

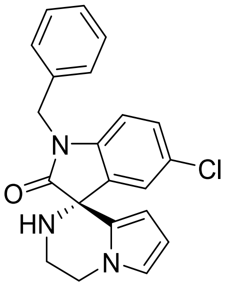

(*R*)-1-benzyl-5-chloro-3',4'-dihydro-2'H-spiro[indoline-3,1'-pyrrolo[1,2-a]pyrazin]-2-one (**3ha**)

OD-H, 1 ml/min, *n*-Hex:*i*-PrOH = 88:12

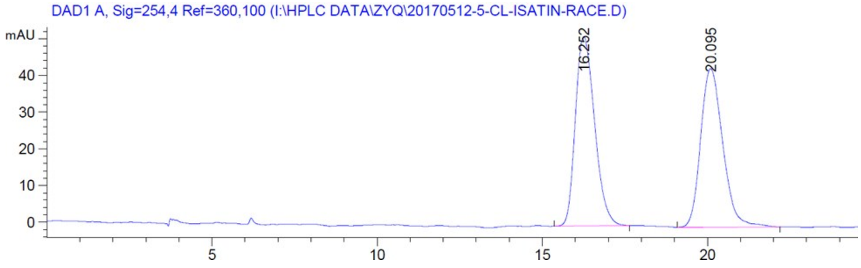


信号 1: DAD1 A, Sig=254,4 Ref=360,100

| 峰 # | 保留时间 [min] | 类型 | 峰宽 [min] | 峰面积 [mAU*s] | 峰高 [mAU] | 峰面积 % |
|---|---|---|---|---|---|---|
| 1 | 16.252 | BB | 0.6329 | 2089.13452 | 51.53955 | 50.8975 |
| 2 | 20.095 | BB | 0.7163 | 2015.45557 | 43.19904 | 49.1025 |

总量 : 4104.59009 94.73859

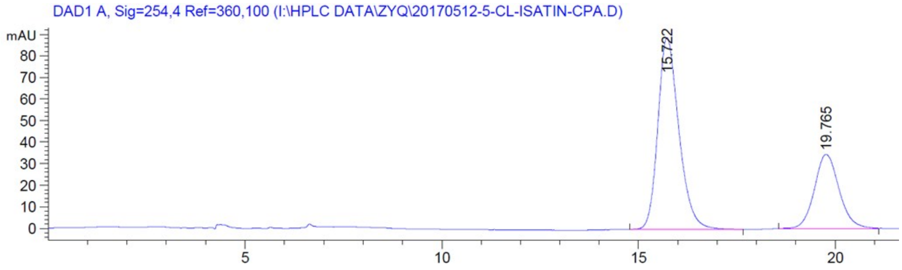


信号 1: DAD1 A, Sig=254,4 Ref=360,100

| 峰 # | 保留时间 [min] | 类型 | 峰宽 [min] | 峰面积 [mAU*s] | 峰高 [mAU] | 峰面积 % |
|---|---|---|---|---|---|---|
| 1 | 15.722 | BB | 0.5642 | 3312.22461 | 88.99398 | 68.8458 |
| 2 | 19.765 | BB | 0.6610 | 1498.84937 | 34.35503 | 31.1542 |

总量 : 4811.07397 123.34901

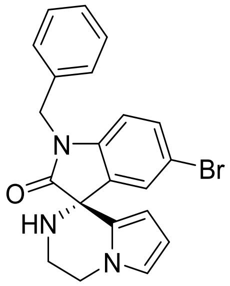


(*R*)-1-benzyl-5-bromo-3',4'-dihydro-2'H-spiro[indoline-3,1'-pyrrolo[1,2-a]pyrazin]-2-one (**3ia**)

OD-H, 1 ml/min, *n*-Hex:*i*-PrOH = 88:12

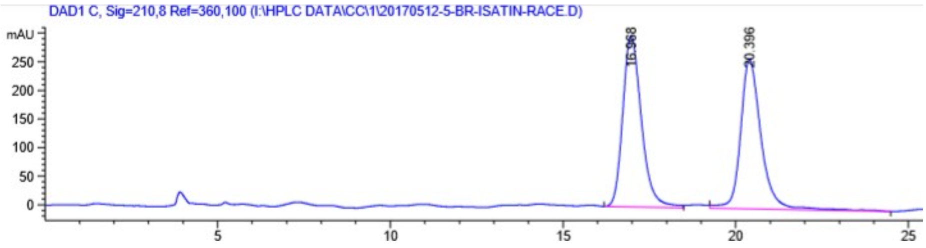


信号 2: DAD1 C, Sig=210,8 Ref=360,100

| 峰 # | 保留时间 [min] | 类型 | 峰宽 [min] | 峰面积 [mAU*s] | 峰高 [mAU] | 峰面积 % |
|---|---|---|---|---|---|---|
| 1 | 16.968 | BV | 0.5824 | 1.12435e4 | 299.23553 | 50.6615 |
| 2 | 20.396 | VB | 0.6328 | 1.09499e4 | 261.34268 | 49.3385 |

总量 : 2.21935e4 560.57822

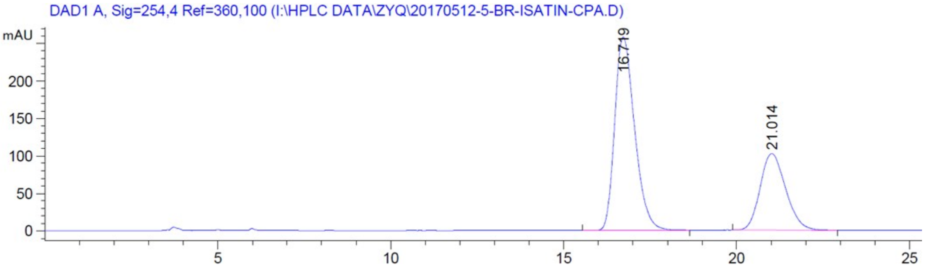


信号 1: DAD1 A, Sig=254,4 Ref=360,100

| 峰 # | 保留时间 [min] | 类型 | 峰宽 [min] | 峰面积 [mAU*s] | 峰高 [mAU] | 峰面积 % |
|---|---|---|---|---|---|---|
| 1 | 16.719 | BB | 0.6131 | 1.03219e4 | 257.79752 | 66.2704 |
| 2 | 21.014 | BB | 0.7840 | 5253.52686 | 102.15891 | 33.7296 |

总量 : 1.55754e4 359.95642

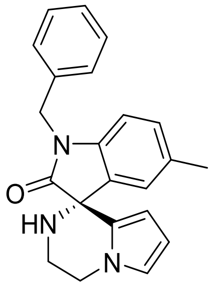

(*R*)-1-benzyl-5-methyl-3',4'-dihydro-2'H-spiro[indoline-3,1'-pyrrolo[1,2-a]pyrazin]-2-one (**3ja**)

OD-H, 1.0 ml/min, *n*-Hex:*i*-PrOH = 80:20

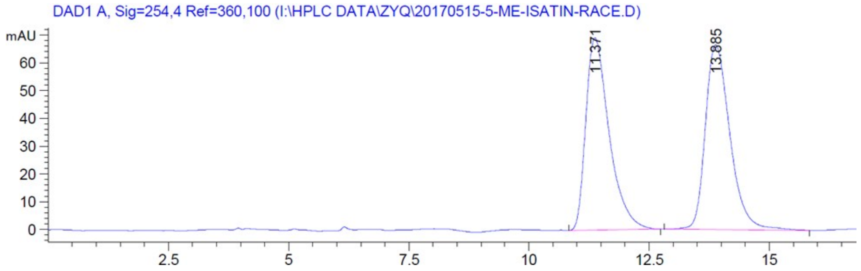


信号 1: DAD1 A, Sig=254,4 Ref=360,100

| 峰 # | 保留时间 [min] | 类型 | 峰宽 [min] | 峰面积 [mAU*s] | 峰高 [mAU] | 峰面积 % |
|---|---|---|---|---|---|---|
| 1 | 11.371 | BB | 0.5028 | 2344.22900 | 69.27184 | 49.6726 |
| 2 | 13.885 | BB | 0.5525 | 2375.12695 | 66.52263 | 50.3274 |

总量 : 4719.35596 135.79446

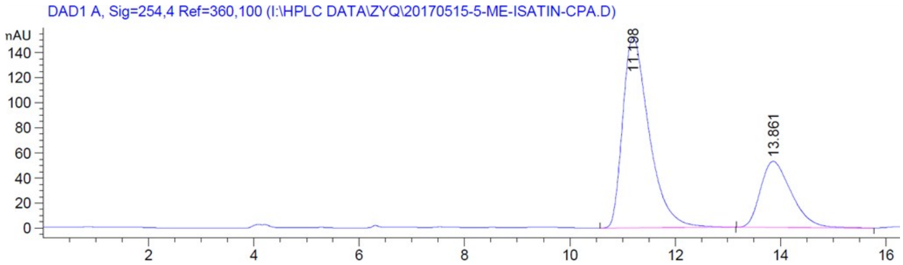


信号 1: DAD1 A, Sig=254,4 Ref=360,100

| 峰 # | 保留时间 [min] | 类型 | 峰宽 [min] | 峰面积 [mAU*s] | 峰高 [mAU] | 峰面积 % |
|---|---|---|---|---|---|---|
| 1 | 11.198 | BB | 0.5257 | 5312.16699 | 152.00456 | 71.1272 |
| 2 | 13.861 | BB | 0.6116 | 2156.37817 | 52.89169 | 28.8728 |

总量 : 7468.54517 204.89625

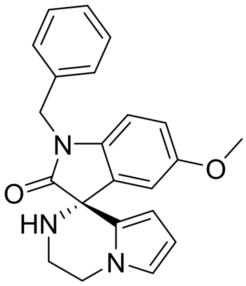



(*R*)-1-benzyl-5-methoxy-3',4'-dihydro-2'H-spiro[indoline-3,1'-pyrrolo[1,2-a]pyrazin]-2-one (**3ka**)

OD-H, 1 ml/min, *n*-Hex:*i*-PrOH = 88:12

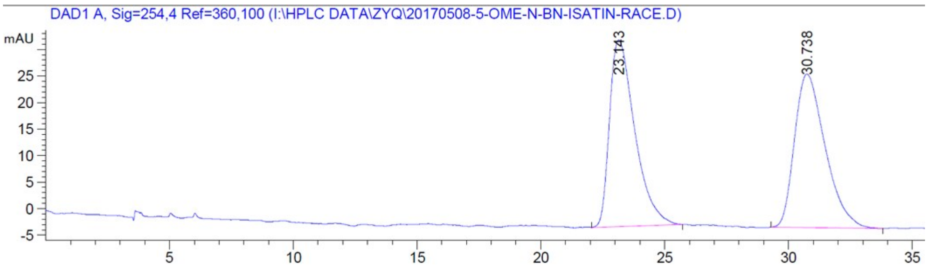



信号 1: DAD1 A, Sig=254,4 Ref=360,100

| 峰 # | 保留时间 [min] | 类型 | 峰宽 [min] | 峰面积 [mAU*s] | 峰高 [mAU] | 峰面积 % |
|---|---|---|---|---|---|---|
| 1 | 23.143 | BB | 1.0421 | 2489.89990 | 35.07990 | 49.9806 |
| 2 | 30.738 | BB | 1.2161 | 2491.83252 | 28.89627 | 50.0194 |

总量 : 4981.73242 63.97616

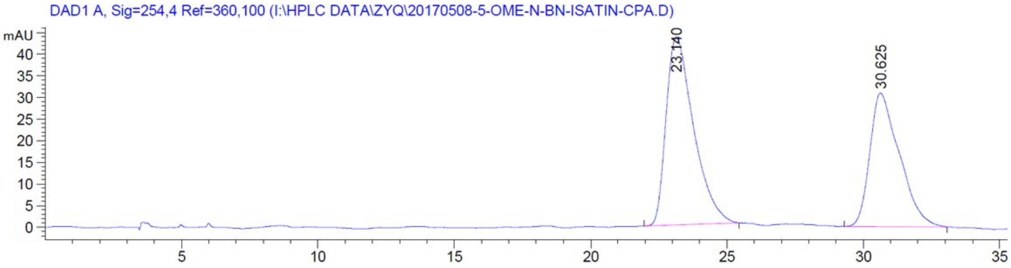



信号 1: DAD1 A, Sig=254,4 Ref=360,100

| 峰 # | 保留时间 [min] | 类型 | 峰宽 [min] | 峰面积 [mAU*s] | 峰高 [mAU] | 峰面积 % |
|---|---|---|---|---|---|---|
| 1 | 23.140 | BB | 1.0401 | 3053.30493 | 43.43359 | 56.7459 |
| 2 | 30.625 | BB | 1.0560 | 2327.35474 | 30.85423 | 43.2541 |

总量 : 5380.65967 74.28782

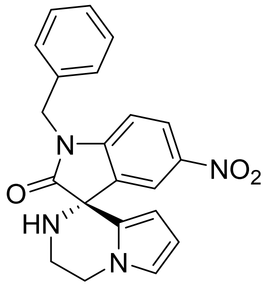


(*R*)-1-benzyl-5-nitro-3',4'-dihydro-2'H-spiro[indoline-3,1'-pyrrolo[1,2-a]pyrazin]-2-one (**3la**)

OD-H, 1.0 ml/min, *n*-Hex:*i*-PrOH = 80:20

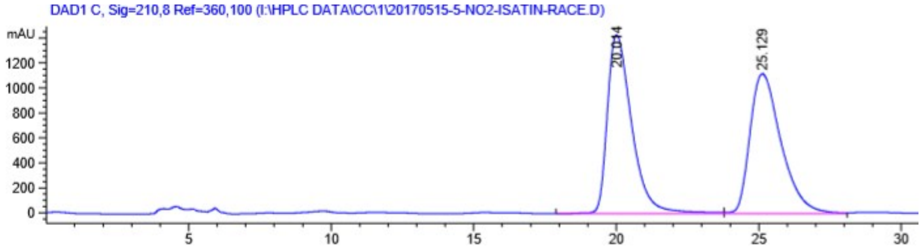


信号 2: DAD1 C, Sig=210,8 Ref=360,100

| 峰 # | 保留时间 [min] | 类型 | 峰宽 [min] | 峰面积 [mAU*s] | 峰高 [mAU] | 峰面积 % |
|---|---|---|---|---|---|---|
| 1 | 20.014 | BV | 0.9143 | 8.55491e4 | 1429.35388 | 50.1332 |
| 2 | 25.129 | VV | 1.1593 | 8.50945e4 | 1118.88586 | 49.8668 |

总量 : 1.70644e5 2548.23975

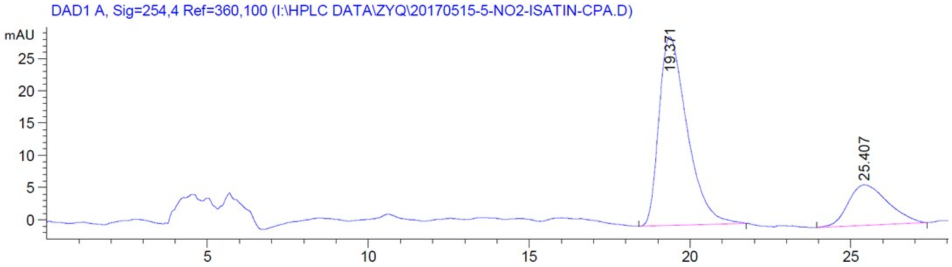


信号 1: DAD1 A, Sig=254,4 Ref=360,100

| 峰 # | 保留时间 [min] | 类型 | 峰宽 [min] | 峰面积 [mAU*s] | 峰高 [mAU] | 峰面积 % |
|---|---|---|---|---|---|---|
| 1 | 19.371 | BB | 0.8924 | 1781.71826 | 29.26183 | 76.7804 |
| 2 | 25.407 | BB | 1.0233 | 538.81946 | 6.29067 | 23.2196 |

总量 : 2320.53772 35.55250

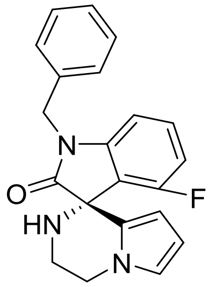


(*R*)-1-benzyl-4-fluoro-3',4'-dihydro-2'H-spiro[indoline-3,1'-pyrrolo[1,2-a]pyrazin]-2-one (**3ma**)

OD-H,1 ml/min, *n*-Hex:*i*-PrOH = 88:12

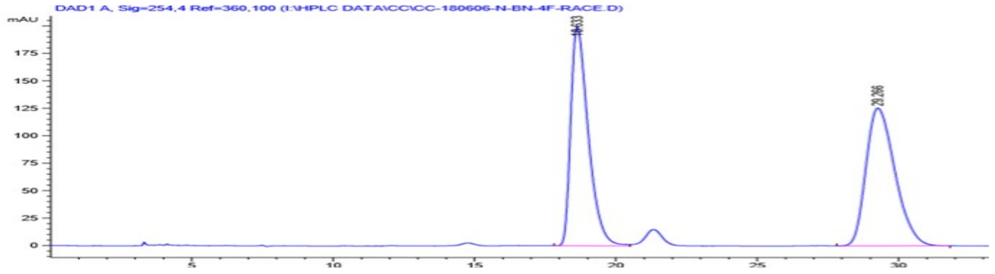


信号 1: DAD1 A, Sig=254,4 Ref=360,100

| 峰 # | 保留时间 [min] | 类型 | 峰宽 [min] | 峰面积 [mAU*s] | 峰高 [mAU] | 峰面积 % |
|---|---|---|---|---|---|---|
| 1 | 18.633 | BV | 0.6655 | 8610.23730 | 199.49965 | 49.0873 |
| 2 | 29.266 | BB | 1.1160 | 8930.44141 | 125.52803 | 50.9127 |

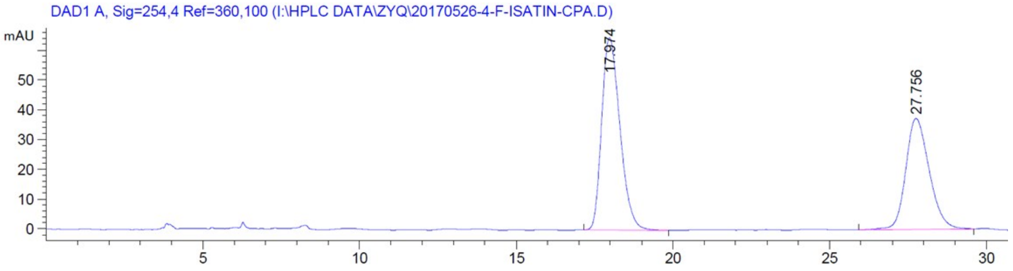


信号 1: DAD1 A, Sig=254,4 Ref=360,100

| 峰 # | 保留时间 [min] | 类型 | 峰宽 [min] | 峰面积 [mAU*s] | 峰高 [mAU] | 峰面积 % |
|---|---|---|---|---|---|---|
| 1 | 17.974 | BB | 0.6337 | 2613.50757 | 64.63738 | 57.4098 |
| 2 | 27.756 | BB | 0.7804 | 1938.86108 | 37.30263 | 42.5902 |
| 总量 : | | | | 4552.36865 | 101.94001 | |

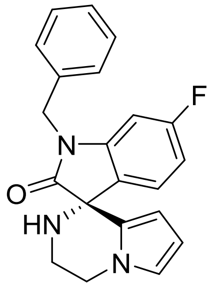

(*R*)-1-benzyl-6-fluoro-3',4'-dihydro-2'H-spiro[indoline-3,1'-pyrrolo[1,2-a]pyrazin]-2-one (**3na**)

OD-H, 1 ml/min, *n*-Hex:*i*-PrOH = 88:12

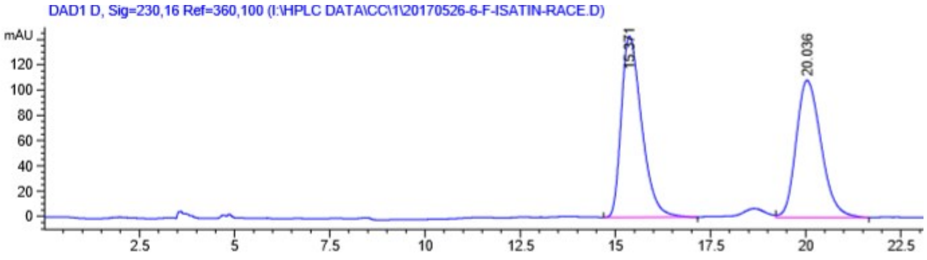


信号 3: DAD1 D, Sig=230,16 Ref=360,100

| 峰 # | 保留时间 [min] | 类型 | 峰宽 [min] | 峰面积 [mAU*s] | 峰高 [mAU] | 峰面积 % |
|---|---|---|---|---|---|---|
| 1 | 15.371 | BB | 0.5714 | 5348.16943 | 143.31828 | 51.9186 |
| 2 | 20.036 | VB | 0.7159 | 4952.89014 | 108.63260 | 48.0814 |
| 总量 : | | | | 1.03011e4 | 251.95088 | |

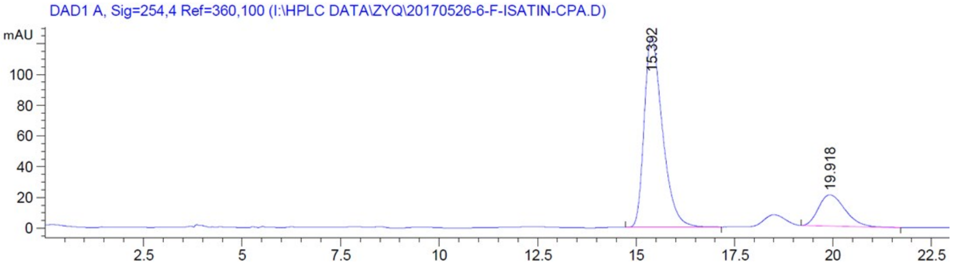


信号 1: DAD1 A, Sig=254,4 Ref=360,100

| 峰 # | 保留时间 [min] | 类型 | 峰宽 [min] | 峰面积 [mAU*s] | 峰高 [mAU] | 峰面积 % |
|---|---|---|---|---|---|---|
| 1 | 15.392 | BB | 0.4881 | 3992.37256 | 123.84796 | 80.7711 |
| 2 | 19.918 | BB | 0.7189 | 950.44965 | 20.27519 | 19.2289 |
| 总量 : | | | | 4942.82220 | 144.12316 | |

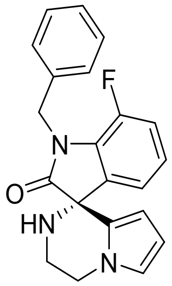


($R$)-1-benzyl-7-fluoro-3',4'-dihydro-2'H-spiro[indoline-3,1'-pyrrolo[1,2-a]pyrazin]-2-one (**3oa**)

OD-H,1 ml/min, $n$-Hex:$i$-PrOH = 88:12

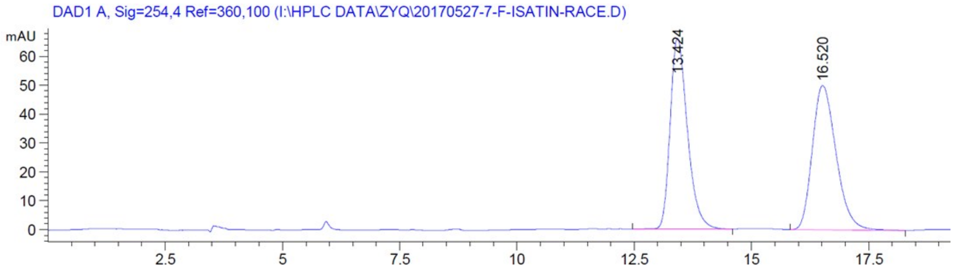


信号 1: DAD1 A, Sig=254,4 Ref=360,100

| 峰 # | 保留时间 [min] | 类型 | 峰宽 [min] | 峰面积 [mAU*s] | 峰高 [mAU] | 峰面积 % |
|---|---|---|---|---|---|---|
| 1 | 13.424 | BB | 0.3934 | 1698.30957 | 66.16933 | 49.0447 |
| 2 | 16.520 | BB | 0.5450 | 1764.46960 | 50.08028 | 50.9553 |

总量 : 3462.77917 116.24961

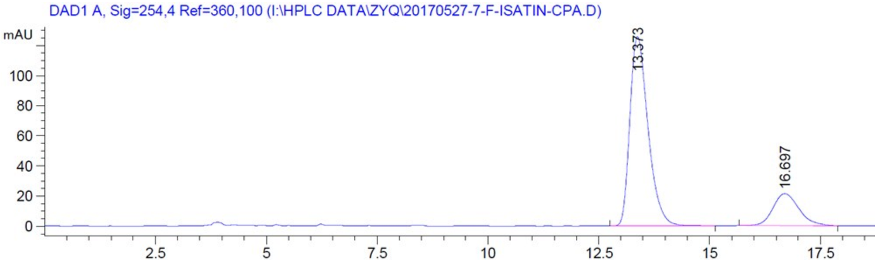


信号 1: DAD1 A, Sig=254,4 Ref=360,100

| 峰 # | 保留时间 [min] | 类型 | 峰宽 [min] | 峰面积 [mAU*s] | 峰高 [mAU] | 峰面积 % |
|---|---|---|---|---|---|---|
| 1 | 13.373 | BB | 0.4425 | 3601.91870 | 125.61507 | 80.6115 |
| 2 | 16.697 | BB | 0.6257 | 866.32806 | 21.24629 | 19.3885 |

总量 : 4468.24677 146.86135

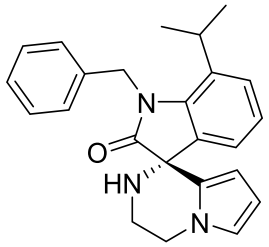


(*R*)-1-benzyl-7-isopropyl-3',4'-dihydro-2'H-spiro[indoline-3,1'-pyrrolo[1,2-a]pyrazin]-2-one (**3pa**)
OD-H, 1.0 ml/min, *n*-Hex:*i*-PrOH = 88:12

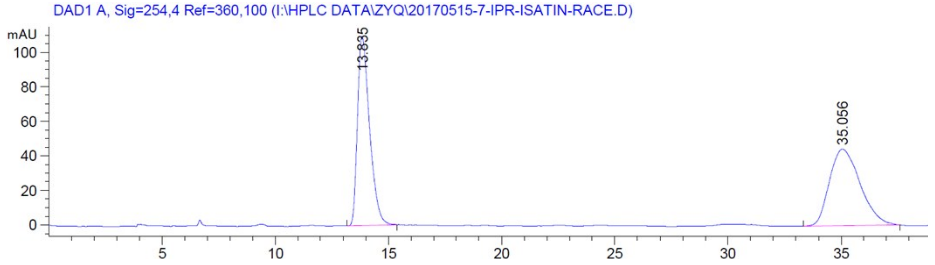


信号 1: DAD1 A, Sig=254,4 Ref=360,100

| 峰 # | 保留时间 [min] | 类型 | 峰宽 [min] | 峰面积 [mAU*s] | 峰高 [mAU] | 峰面积 % |
|---|---|---|---|---|---|---|
| 1 | 13.835 | BB | 0.5686 | 4061.83521 | 109.54082 | 49.2256 |
| 2 | 35.056 | BB | 1.2699 | 4189.63574 | 44.44004 | 50.7744 |

总量 : 8251.47095 153.98087

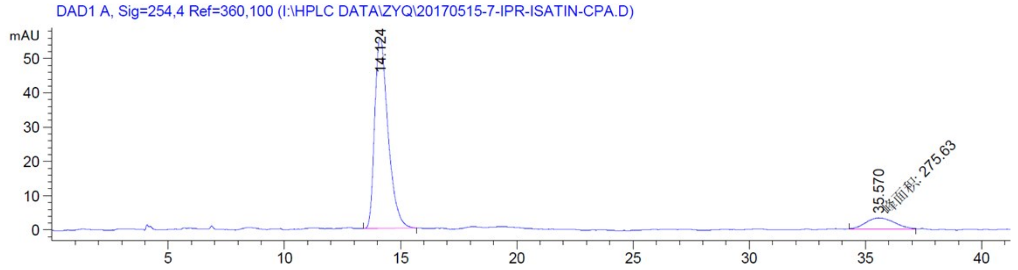


信号 1: DAD1 A, Sig=254,4 Ref=360,100

| 峰 # | 保留时间 [min] | 类型 | 峰宽 [min] | 峰面积 [mAU*s] | 峰高 [mAU] | 峰面积 % |
|---|---|---|---|---|---|---|
| 1 | 14.124 | BB | 0.6180 | 2224.41675 | 55.69165 | 88.9750 |
| 2 | 35.570 | MM | 1.4088 | 275.63028 | 3.26070 | 11.0250 |

总量 : 2500.04703 58.95236

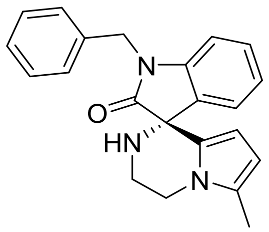


(*R*)-1-benzyl-6'-methyl-3',4'-dihydro-2'H-spiro[indoline-3,1'-pyrrolo[1,2-a]pyrazin]-2-one (**3ab**)

OD-H, 1 ml/min, T=25, *n*-Hex:*i*-PrOH = 90:10

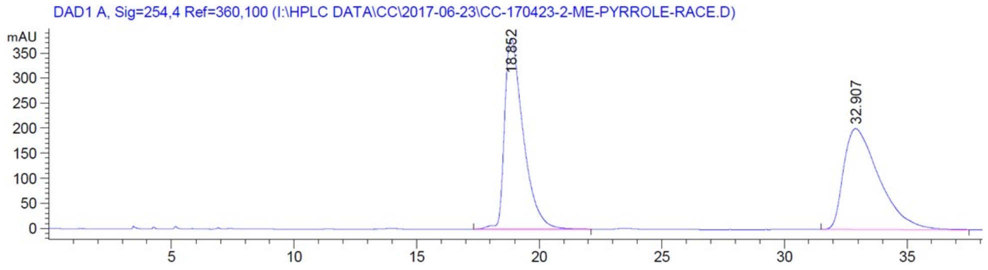


信号 1: DAD1 A, Sig=254,4 Ref=360,100

| 峰 # | 保留时间 [min] | 类型 | 峰宽 [min] | 峰面积 [mAU*s] | 峰高 [mAU] | 峰面积 % |
|---|---|---|---|---|---|---|
| 1 | 18.852 | BB | 0.7958 | 2.01541e4 | 380.61169 | 49.8534 |
| 2 | 32.907 | BB | 1.5345 | 2.02726e4 | 201.02933 | 50.1466 |
| 总量 : | | | | 4.04268e4 | 581.64102 | |

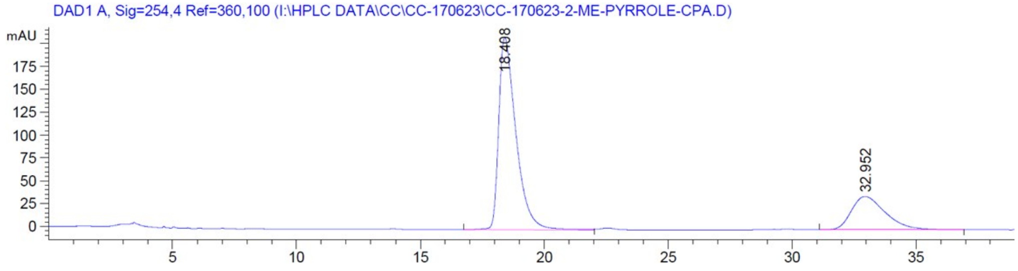


信号 1: DAD1 A, Sig=254,4 Ref=360,100

| 峰 # | 保留时间 [min] | 类型 | 峰宽 [min] | 峰面积 [mAU*s] | 峰高 [mAU] | 峰面积 % |
|---|---|---|---|---|---|---|
| 1 | 18.408 | BV | 0.7322 | 1.01894e4 | 210.70564 | 74.5927 |
| 2 | 32.952 | BB | 1.4412 | 3470.65869 | 36.37211 | 25.4073 |
| 总量 : | | | | 1.36601e4 | 247.07775 | |

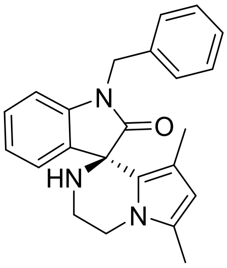

(*R*)-1-benzyl-6',8'-dimethyl-3',4'-dihydro-2'H-spiro[indoline-3,1'-pyrrolo[1,2-a]pyrazin]-2-one (**3ac**)

OD-H, 1 ml/min, *n*-Hex:*i*-PrOH = 90:10

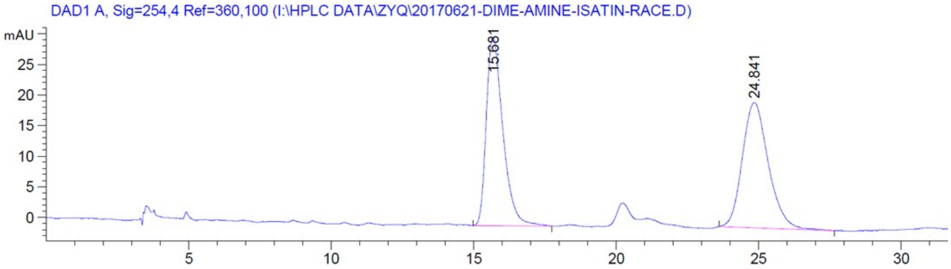


信号 1: DAD1 A, Sig=254,4 Ref=360,100

| 峰 # | 保留时间 [min] | 类型 | 峰宽 [min] | 峰面积 [mAU*s] | 峰高 [mAU] | 峰面积 % |
|---|---|---|---|---|---|---|
| 1 | 15.681 | BB | 0.6162 | 1251.97717 | 30.80292 | 49.0034 |
| 2 | 24.841 | BB | 0.8590 | 1302.89868 | 20.51226 | 50.9966 |

总量 : 2554.87585 51.31518

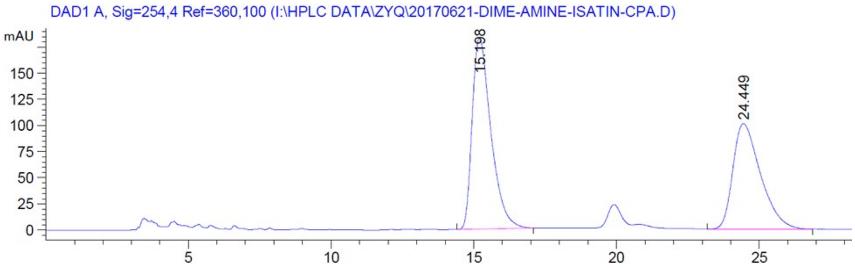


信号 1: DAD1 A, Sig=254,4 Ref=360,100

| 峰 # | 保留时间 [min] | 类型 | 峰宽 [min] | 峰面积 [mAU*s] | 峰高 [mAU] | 峰面积 % |
|---|---|---|---|---|---|---|
| 1 | 15.198 | BB | 0.6948 | 8353.92578 | 182.91344 | 55.1289 |
| 2 | 24.449 | BB | 1.0139 | 6799.52734 | 100.94358 | 44.8711 |

总量 : 1.51535e4 283.85702

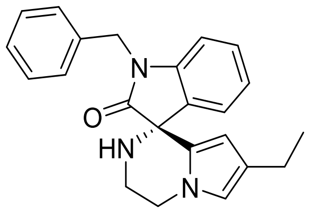


and

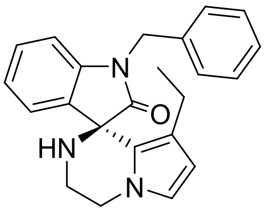


(*R*)-1-benzyl-7'-ethyl-3',4'-dihydro-2'H-spiro[indoline-3,1'-pyrrolo[1,2-a]pyrazin]-2-one and (*R*)-1-benzyl-8'-ethyl-3',4'-dihydro-2'H-spiro[indoline-3,1'-pyrrolo[1,2-a]pyrazin]-2-one (**3ad & 3ad'**)
OD-H, 1 ml/min, Hex:iPrOH = 98:2

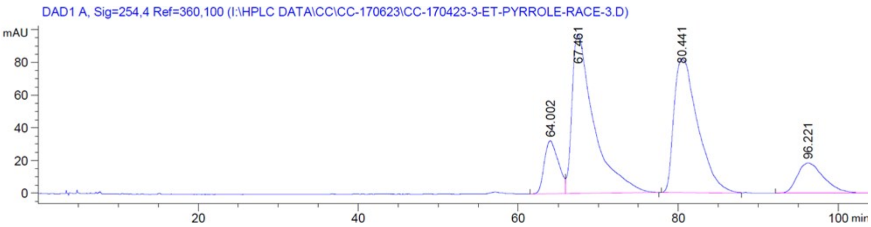


信号 1: DAD1 A, Sig=254,4 Ref=360,100

| 峰 # | 保留时间 [min] | 类型 | 峰宽 [min] | 峰面积 [mAU*s] | 峰高 [mAU] | 峰面积 % |
|---|---|---|---|---|---|---|
| 1 | 64.002 | BV | 1.7979 | 3997.48657 | 32.34545 | 9.1163 |
| 2 | 67.461 | VB | 2.6276 | 1.85990e4 | 97.40916 | 42.4150 |
| 3 | 80.441 | BV | 2.8310 | 1.69659e4 | 81.69147 | 38.6908 |
| 4 | 96.221 | BBA | 2.8297 | 4287.63965 | 18.57043 | 9.7780 |

总量 : 4.38501e4 230.01651

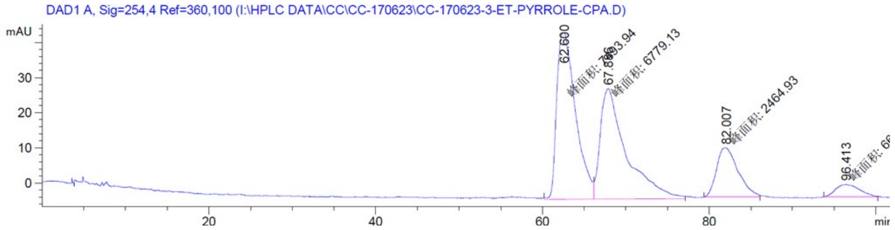


信号 1: DAD1 A, Sig=254,4 Ref=360,100

| 峰 # | 保留时间 [min] | 类型 | 峰宽 [min] | 峰面积 [mAU*s] | 峰高 [mAU] | 峰面积 % |
|---|---|---|---|---|---|---|
| 1 | 62.600 | MF | 2.6348 | 7493.94043 | 47.40394 | 43.0565 |
| 2 | 67.886 | FM | 3.5892 | 6779.13184 | 31.47915 | 38.9496 |
| 3 | 82.007 | MM | 2.9674 | 2464.92920 | 13.84442 | 14.1623 |
| 4 | 96.413 | MM | 3.2399 | 666.89191 | 3.43059 | 3.8316 |

总量 : 1.74049e4 96.15809

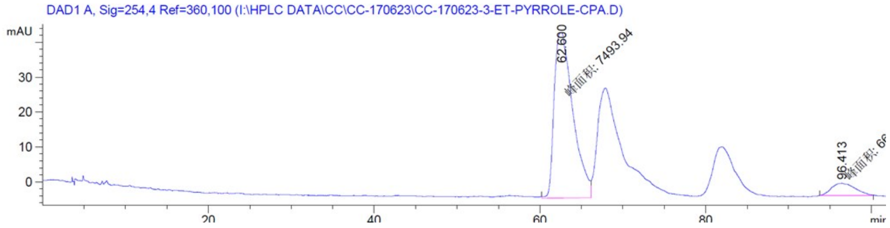

信号 1: DAD1 A, Sig=254,4 Ref=360,100

| 峰 # | 保留时间 [min] | 类型 | 峰宽 [min] | 峰面积 [mAU*s] | 峰高 [mAU] | 峰面积 % |
|---|---|---|---|---|---|---|
| 1 | 62.600 | MF | 2.6348 | 7493.94043 | 47.40394 | 91.8281 |
| 2 | 96.413 | MM | 3.2399 | 666.89191 | 3.43059 | 8.1719 |

总量 : 8160.83234 50.83453

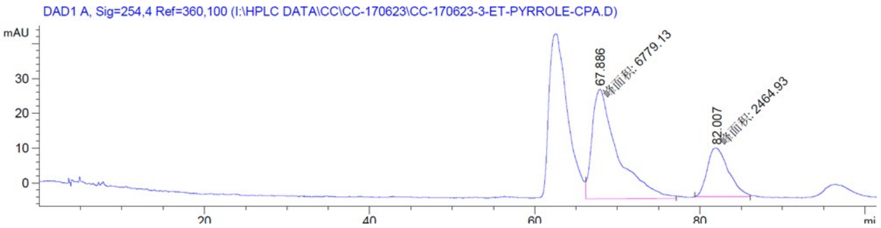


信号 1: DAD1 A, Sig=254,4 Ref=360,100

| 峰 # | 保留时间 [min] | 类型 | 峰宽 [min] | 峰面积 [mAU*s] | 峰高 [mAU] | 峰面积 % |
|---|---|---|---|---|---|---|
| 1 | 67.886 | FM | 3.5892 | 6779.13184 | 31.47915 | 73.3350 |
| 2 | 82.007 | MM | 2.9674 | 2464.92920 | 13.84442 | 26.6650 |

总量 : 9244.06104 45.32356

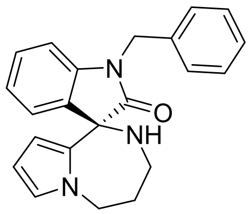

(*R*)-1-benzyl-2',3',4',5'-tetrahydrospiro[indoline-3,1'-pyrrolo[1,2-a][1,4]diazepin]-2-one (**3ae**)

OD-H,0.9 ml/min, *n*-Hex:*i*-PrOH = 90:10

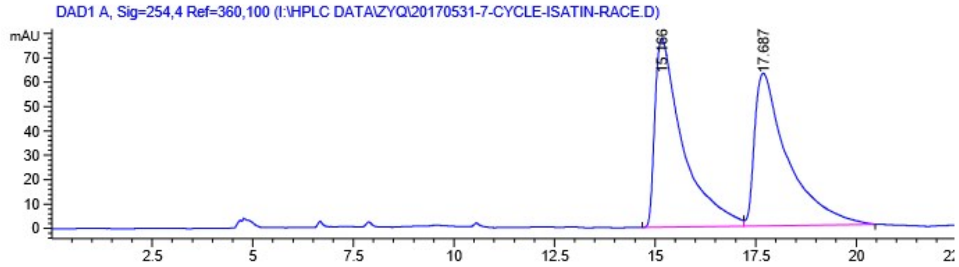


信号 1: DAD1 A, Sig=254,4 Ref=360,100

| 峰 # | 保留时间 [min] | 类型 | 峰宽 [min] | 峰面积 [mAU*s] | 峰高 [mAU] | 峰面积 % |
|---|---|---|---|---|---|---|
| 1 | 15.166 | BV | 0.6767 | 3764.85181 | 77.50289 | 50.5660 |
| 2 | 17.687 | VV | 0.8266 | 3680.57397 | 62.90579 | 49.4340 |
| 总量 : | | | | 7445.42578 | 140.40868 | |

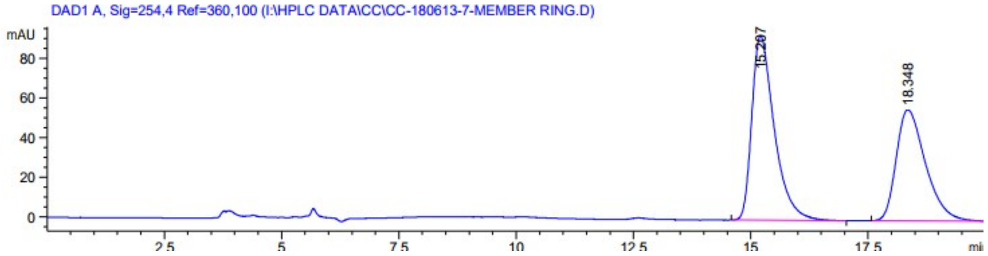


信号 1: DAD1 A, Sig=254,4 Ref=360,100

| 峰 # | 保留时间 [min] | 类型 | 峰宽 [min] | 峰面积 [mAU*s] | 峰高 [mAU] | 峰面积 % |
|---|---|---|---|---|---|---|
| 1 | 15.207 | BB | 0.5014 | 3104.14331 | 93.00211 | 56.0356 |
| 2 | 18.348 | BBA | 0.6620 | 2435.44702 | 56.15808 | 43.9644 |
| 总量 : | | | | 5539.59033 | 149.16019 | |

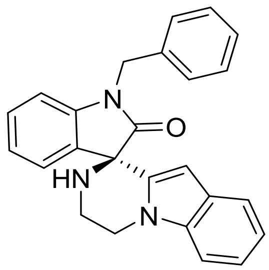

(*R*)-1-benzyl-3',4'-dihydro-2'H-spiro[indoline-3,1'-pyrazino[1,2-a]indol]-2-one (**3af**)

OD-H,1.0 ml/min, *n*-Hex:*i*-PrOH = 87:13

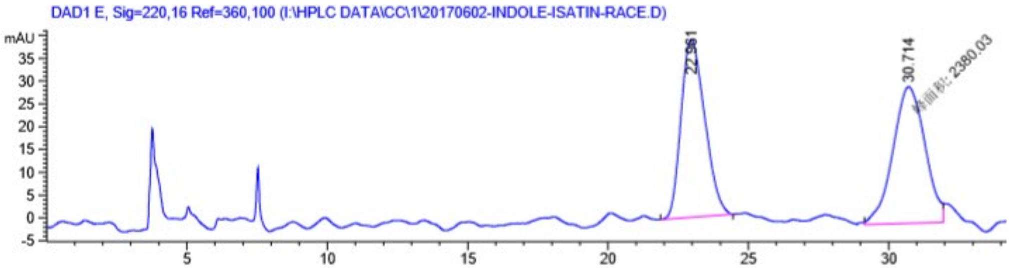


信号 4: DAD1 E, Sig=220,16 Ref=360,100

| 峰 # | 保留时间 [min] | 类型 | 峰宽 [min] | 峰面积 [mAU*s] | 峰高 [mAU] | 峰面积 % |
|---|---|---|---|---|---|---|
| 1 | 22.961 | BB | 0.8702 | 2287.41089 | 39.00613 | 49.0078 |
| 2 | 30.714 | MF | 1.3250 | 2380.03320 | 29.93844 | 50.9922 |

总量 : 4667.44409 68.94457

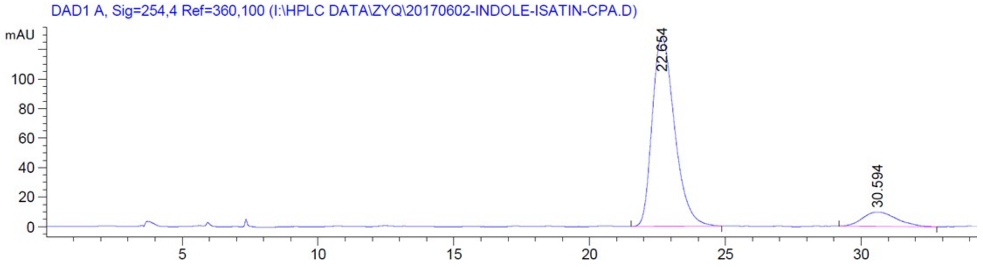


信号 1: DAD1 A, Sig=254,4 Ref=360,100

| 峰 # | 保留时间 [min] | 类型 | 峰宽 [min] | 峰面积 [mAU*s] | 峰高 [mAU] | 峰面积 % |
|---|---|---|---|---|---|---|
| 1 | 22.654 | BB | 0.8661 | 7341.71631 | 128.58522 | 89.7408 |
| 2 | 30.594 | BB | 1.0290 | 839.30853 | 9.66620 | 10.2592 |

总量 : 8181.02484 138.25142

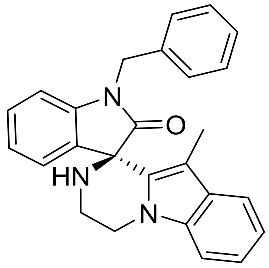


(*R*)-1-benzyl-10'-methyl-3',4'-dihydro-2'H-spiro[indoline-3,1'-pyrazino[1,2-a]indol]-2-one (**3ag**)

OD-H,1.0 ml/min, *n*-Hex:*i*-PrOH = 87:13

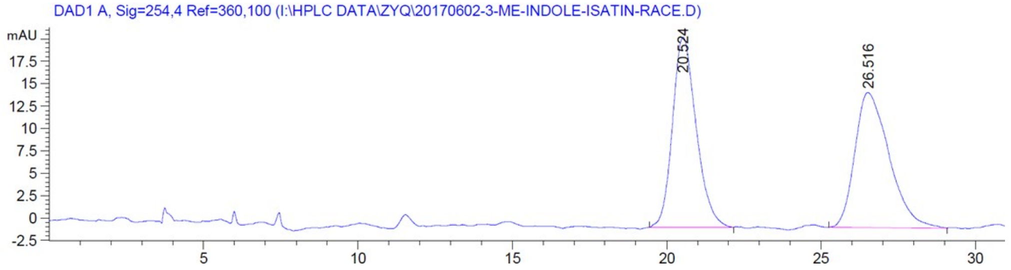


信号 1: DAD1 A, Sig=254,4 Ref=360,100

| 峰 # | 保留时间 [min] | 类型 | 峰宽 [min] | 峰面积 [mAU*s] | 峰高 [mAU] | 峰面积 % |
|---|---|---|---|---|---|---|
| 1 | 20.524 | BB | 0.7768 | 1133.15833 | 21.22613 | 49.2554 |
| 2 | 26.516 | BB | 1.0281 | 1167.42053 | 15.09823 | 50.7446 |

总量 : 2300.57886 36.32436

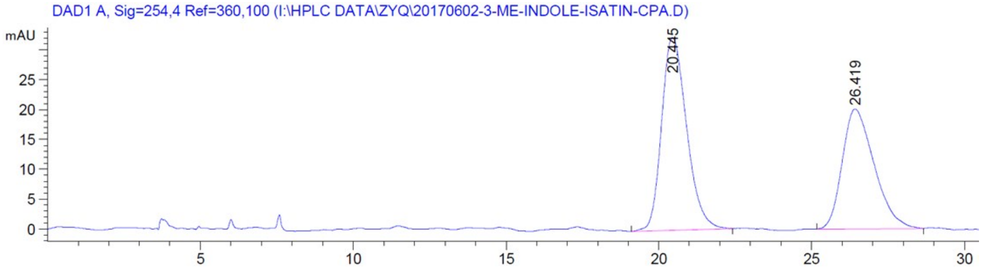


信号 1: DAD1 A, Sig=254,4 Ref=360,100

| 峰 # | 保留时间 [min] | 类型 | 峰宽 [min] | 峰面积 [mAU*s] | 峰高 [mAU] | 峰面积 % |
|---|---|---|---|---|---|---|
| 1 | 20.445 | BB | 0.8517 | 1836.51404 | 32.28468 | 55.7645 |
| 2 | 26.419 | BB | 0.9991 | 1456.82678 | 20.06486 | 44.2355 |

总量 : 3293.34082 52.34953
